# Telescope: Characterization of the retrotranscriptome by accurate estimation of transposable element expression

**DOI:** 10.1371/journal.pcbi.1006453

**Published:** 2019-09-30

**Authors:** Matthew L. Bendall, Miguel de Mulder, Luis Pedro Iñiguez, Aarón Lecanda-Sánchez, Marcos Pérez-Losada, Mario A. Ostrowski, R. Brad Jones, Lubbertus C. F. Mulder, Gustavo Reyes-Terán, Keith A. Crandall, Christopher E. Ormsby, Douglas F. Nixon

**Affiliations:** 1 Computational Biology Institute, Milken Institute School of Public Health, George Washington University, Washington, D.C., United States of America; 2 Division of Infectious Diseases, Department of Medicine, Weill Cornell Medicine, New York, N.Y., United States of America; 3 Center for Research in Infectious Diseases (CIENI), Instituto Nacional de Enfermedades Respiratorias, Mexico City, Mexico; 4 Department of Biostatistics and Bioinformatics, Milken Institute School of Public Health, George Washington University, Washington, D.C., United States of America; 5 CIBIO-InBIO, Centro de Investigação em Biodiversidade e Recursos Genéticos, Universidade do Porto, Campus Agrário de Vairão, Vairão, Portugal; 6 Department of Immunology, University of Toronto, Toronto, Ontario, Canada; 7 Keenan Research Centre for Biomedical Science of St. Michael's Hospital, Toronto, Ontario, Canada; 8 Department of Microbiology, Icahn School of Medicine at Mount Sinai, New York, New York, United States of America; 9 The Global Health and Emerging Pathogens Institute, Icahn School of Medicine at Mount Sinai, New York, New York, United States of America; Stony Brook University, UNITED STATES

## Abstract

Characterization of Human Endogenous Retrovirus (HERV) expression within the transcriptomic landscape using RNA-seq is complicated by uncertainty in fragment assignment because of sequence similarity. We present Telescope, a computational software tool that provides accurate estimation of transposable element expression (retrotranscriptome) resolved to specific genomic locations. Telescope directly addresses uncertainty in fragment assignment by reassigning ambiguously mapped fragments to the most probable source transcript as determined within a Bayesian statistical model. We demonstrate the utility of our approach through single locus analysis of HERV expression in 13 ENCODE cell types. When examined at this resolution, we find that the magnitude and breadth of the retrotranscriptome can be vastly different among cell types. Furthermore, our approach is robust to differences in sequencing technology and demonstrates that the retrotranscriptome has potential to be used for cell type identification. We compared our tool with other approaches for quantifying transposable element (TE) expression, and found that Telescope has the greatest resolution, as it estimates expression at specific TE insertions rather than at the TE subfamily level. Telescope performs highly accurate quantification of the retrotranscriptomic landscape in RNA-seq experiments, revealing a differential complexity in the transposable element biology of complex systems not previously observed. Telescope is available at https://github.com/mlbendall/telescope.

## Author summary

Almost half of the human genome is composed of transposable elements (TEs), but their contribution to the transcriptome, their cell-type specific expression patterns, and their role in disease remains poorly understood. Recent studies have found many elements to be actively expressed and involved in key cellular processes. For example, human endogenous retroviruses (HERVs) are reported to be involved in human embryonic stem cell differentiation. Discovering which exact HERVs are differentially expressed in RNA-seq data would be a major advance in understanding such processes. However, because HERVs have a high level of sequence similarity it is hard to identify which exact HERV is differentially expressed. To solve this problem, we developed a computer program which addressed uncertainty in fragment assignment by reassigning ambiguously mapped fragments to the most probable source transcript as determined within a Bayesian statistical model. We call this program, “Telescope”. We then used Telescope to identify HERV expression in 13 well-studied cell types from the ENCODE consortium and found that different cell types could be characterized by enrichment for different HERV families, and for locus specific expression. We also showed that Telescope performed better than other methods currently used to determine TE expression. The use of this computational tool to examine new and existing RNA-seq data sets may lead to new understanding of the roles of TEs in health and disease.

This is a *PLOS Computational Biology* Methods paper.

## Introduction

Transposable elements (TEs) represent the largest class of biochemically functional DNA elements in mammalian genomes[1,2] comprising nearly 50% of the human genome. As many of these transcriptionally active elements originated as retroelements, we refer to the set of RNA molecules transcribed from these elements in a population of cells as the retrotranscriptome. The contribution of the retrotranscriptome to the total transcriptome, cell-type specific expression patterns, and the role of retroelement transcripts in disease remain poorly understood[3]. Although most TEs are hypothesized to be transcriptionally silent (due to accumulated mutations), recent studies have found many elements to be actively expressed and involved in key cellular processes. For example, aberrant expression of LINE-1 (L1) elements, the most expansive group of TEs, has been implicated in the pathogenesis of cancer[4–7], while human endogenous retroviruses (HERVs) are reported to be involved in human embryonic stem cell differentiation[8,9] and in the pathogenesis of amyotrophic lateral sclerosis[10]. We, and others, have shown that HIV-1 infection increases HERV transcription[11–15]. These lines of evidence therefore indicate that TEs have important roles in the regulation of human health and disease.

The ability to observe and quantify TE expression, especially the specific genomic locations of active elements, is crucial for understanding the molecular basis underlying a wide range of conditions and diseases[16]. Traditional techniques for interrogating the TE transcriptome include quantitative PCR[17,18] and RNA expression microarrays[19–23]. However, these techniques are unable to discover elements not specifically targeted by the assay, and may fail to detect rare, previously unknown, or weakly expressed transcripts. High-throughput RNA sequencing (RNA-seq) promises to overcome many of these shortcomings, enabling highly sensitive detection of transcripts across a wide dynamic range. Mathematical and computational approaches for transcriptome quantification using RNA-seq are well established[24,25] (reviewed by Garber et al.[26]) and provide researchers with reproducible analytical pipelines[27,28]. Such approaches are highly effective at quantifying transcripts when sequenced fragments can be uniquely aligned to the reference genome, since the original genomic template for each transcript can be unambiguously identified[29,30]. In contrast, sequencing fragments that originate from repetitive sequences often have high scoring alignments to many genomic locations, leading to uncertainty in fragment mapping and the derived transcript counts. Issues arising from these “multimapping” or “ambiguous” fragments are well known and are often addressed by masking repetitive sequences or otherwise discarding ambiguous fragments[31–33]. The disadvantage of ignoring repeats is that interesting biological phenomena, including those involving TEs, are missed[31]. Several approaches have been proposed that account for read mapping uncertainty using statistical models. The most common approach, described by Li et al.[34,35], involves modeling read assignments using a mixture model, with expression levels as mixture weights and fragment assignments as latent variables; model parameters are then estimated using an expectation-maximization algorithm. Several variations on this model have been proposed, such as modeling read counts instead of individual reads (MMSEQ[36]) or using Markov chain Monte Carlo (MCMC) to sample model parameters (BitSeq[37]). A few approaches deviate from the mixture model approach; notably, MMR instead evaluates alignments by minimizing a loss function[38]. To our knowledge, none of these packages have been adapted specifically for quantifying TE expression.

A growing field of study is now interested in using high-throughput sequencing to characterize the retrotranscriptome[8,9,39–41]. Instead of considering repetitive sequences as a source of noise that interferes with gene expression analysis, the TEs themselves are the features of interest. Three general approaches are used to deal with challenges of aligning short sequencing reads to repetitive elements. i) “Family-level” approaches combine read counts across multiple instances of a TE subfamily, since fragments mapping to multiple genomic locations can often be uniquely assigned to a single repeat subfamily. This approach provides valuable information about which TE subfamilies may be differentially regulated, but lacks the resolution needed to identify specific expressed elements. ii) “Heuristic” approaches simplify the problem of multi-mapped fragments by examining alignments and using filtering criteria to resolve ambiguity. Examples of heuristic approaches include discarding ambiguous reads (unique counts), randomly assigning ambiguous reads to one of its best scoring alignments (best counts), or dividing counts among possible alignments (fractional counts). Finally, iii) “statistical” approaches implement a statistical model that estimates the most probable assignment of fragments given underlying assumptions about the generating process and the observed data.

Several existing software packages have implemented these approaches specifically for TE quantification. RepEnrich[42,43] maps reads to “pseudogenomes” composed of multiple loci belonging to the same subfamily, then uses a fractional counts heuristic to resolve any remaining ambiguous fragments. TEtranscripts[44] and SalmonTE[45] are both statistical approaches that use mixture models estimated by expectation-maximization. The main difference between these approaches is that TEtranscripts begins with genome alignment, while SalmonTE adapts the Salmon[46] approach of quasi-alignment to transcriptome sequences. Like MMSEQ, SalmonTE also uses equivalence classes to reduce the effort needed for parameter optimization. By default, all three TE quantification approaches summarize estimates by subfamily.

Here, we introduce Telescope, a tool which provides accurate estimation of TE expression resolved to specific genomic locations. Our approach directly addresses uncertainty in fragment assignment by reassigning ambiguously mapped fragments to the most probable source transcript as determined within a Bayesian statistical model. We implement our approach using a descriptive statistical model of the RNA-seq process and use an iterative algorithm to optimize model parameters. We use Telescope to investigate the expression of HERVs in cell types from the ENCODE consortium.

## Results

### Telescope: Single locus resolution of transposable element expression

Resolution of transposable element (including those of human endogenous retroviruses, HERVs) expression from RNA-seq data sets has been complicated by the many similarities of these repetitive elements. Telescope is a computational pipeline program that solves the problem of ambiguously aligned fragments by assigning each sequenced fragment to its most likely transcript of origin. We assume that the number of fragments generated by a transcript is proportional to the amount of transcript present in the sample; thus, the most likely source template for a randomly selected fragment is a function of its alignment uncertainty and the relative transcript abundances. Telescope describes this relationship using a Bayesian mixture model where the estimated parameters include the relative transcript abundances and the latent variables define the possible source templates for each fragment[47].

**Fig 1:**
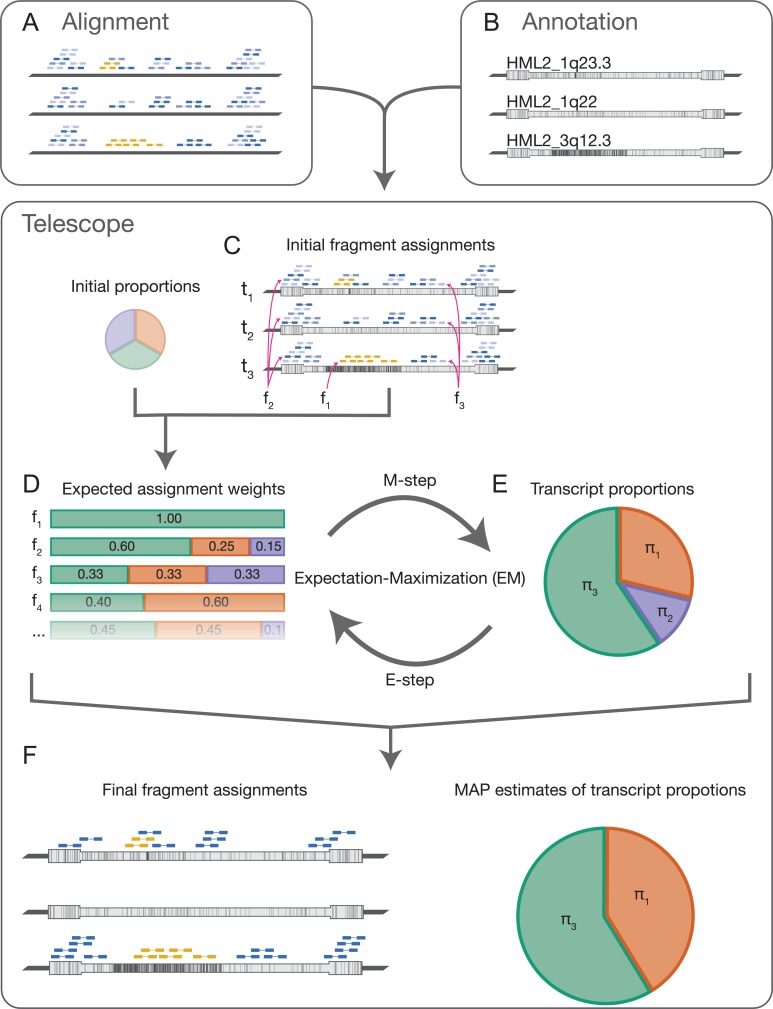
Telescope conceptual overview. Telescope requires as input an alignment to the reference genome (A) and an annotation of transposable element locations (B). Alignments should identify many possible high-scoring mappings for each fragment. Fragments shown in gold represent unique mapping locations, dark blue fragments represent a best alignment out of several possible alignments, and light blue fragments represent alignments with suboptimal alignment scores (A). Annotations describe the locations of TE transcripts to be quantified. Three representative HML-2 loci are shown; vertical lines represent differences from the HML-2 consensus sequence (B). Telescope intersects the aligned fragments with annotated TE loci; fragments with no alignments intersecting the annotation are discarded (C). The set of alignments and corresponding alignment scores for each fragment are used to calculate the expected assignment weights, initially assuming equal expression for all elements (D). For example, fragment f_1_ aligns uniquely to locus t_3_, and has an expected assignment weight of 1; the best alignment for f_2_ is to t_3_ and has a weight of 0.6; f_3_ aligns equally well to t_1_, t_2_, and t_3_ (C,D). The assignment weights estimated in (D) are used to find the maximum likelihood estimate (MLE) for the proportion of each transcript (E). Next, we update the expected assignment weights, now assuming that the MLE represents our best estimate of transcript expression (D,E). The steps in panels (D) and (E) describe an expectation-maximization procedure, and we further refine the assignment weights and MLE by iterating until parameter estimates converge. Telescope produces a report that includes the maximum a posteriori estimate of the transcript proportions and the final number of fragments assigned to each transcript, as well as an updated alignment including the final fragment assignments (F).

The first step in this approach is to independently align each fragment to the reference genome; the alignment method should search for multiple valid alignments for each fragment and report all alignments that meet or exceed a minimum score threshold (Fig 1A). Next, alignments are tested for overlap with known TE transcripts; transcript assignments for each fragment are weighted by the score of the corresponding alignment (Fig 1B and 1C). In our test cases, we typically find that less than 50% of the fragments aligning to TEs can be uniquely assigned to a single genomic location and many fragments have more than 20 possible originating transcripts.

Telescope uses a Bayesian mixture model to represent transcript proportions and unobserved source templates and estimates model parameters using an expectation-maximization algorithm. In the expectation step (E-step), the expected value of the source template for each fragment is calculated under current estimates of transcript abundance (Fig 1D). The maximization step (M-step) finds maximum *a posteriori* estimates of the transcript abundance dependent on the expected values from the E-step (Fig 1E). These steps are repeated until parameter estimates converge (Fig 1D and 1E). Telescope reports the proportion of fragments generated by each transcript and the expected transcript of origin for each fragment (Fig 1F). The final counts estimated by Telescope correspond to actual observations of sequenced fragments and are suitable for normalization and differential analysis by a variety of methods. The software also provides an updated alignment with final fragment assignments that can be examined using common genome visualization tools.

The core statistical model implemented in Telescope is based on the read reassignment model described by Francis et al.[47] and is similar to existing models for resolving mapping uncertainty[34,35,44,45]. Three main differences distinguish our model from existing models. First, our model includes a reassignment parameter, theta, that is absent in other models. This parameter effectively penalizes ambiguous alignments and may be important in cases where many highly similar transcripts are present. Second, our model includes an additional mixture component for fragments that map outside of the known transcriptome, accounting for missing transcripts in the annotation. Finally, our model does not use equivalence classes; reassignment occurs at the fragment level.

To demonstrate that our algorithm can truly resolve repetitive element expression to precise genomic locations, we generated sequencing fragments from a single genomic locus in silico and used Telescope to resolve alignment ambiguity and quantify expression. The locus selected was HML2_1q22 (HERV-K102), an HML-2 provirus that is highly similar to several other HML-2 loci[48] and should thus generate many ambiguously mapping fragments. All of the simulated fragments aligned to multiple genomic locations, and most of these (68.4%) had multiple distinct alignments sharing the same “best” alignment score (S1 Fig). Fragments mapped to 71 different HERV proviruses, including 58 HML-2 loci. After using our model to identify the most probable source locus for each fragment, we found that all fragments could be confidently assigned to HML2_1q22 with greater than 99% posterior probability (S1 Fig). This is possible because our model effectively reweights ambiguous alignments by borrowing strength from nearby alignments that are unique or high-scoring. In this case, there were no uniquely aligned fragments within HML2_1q22, but many fragments had best-scoring alignments to this locus. This result demonstrates that our approach can accurately reassign ambiguously mapping fragments and thus enables accurate expression quantification at single-locus resolution.

### Determination of HERV expression in major cell types from the ENCODE consortium

To investigate HERV expression in a robust way across a diverse platform of cell types we relied on publicly available RNA-seq data. The ENCODE data project is an invaluable source of genomic data from disparate sources and provides the opportunity to mine the transposable element expression in a setting of maximum genomic information. We profiled 13 human cell types, including common lines designated by the ENCODE consortium, as well as primary cell types, and applied our approach to determine HERV expression across the spectrum of human cell types, including normal or transformed, and contrasting cell lines with primary cells (Table 1, S1 Table).

**Table 1:** ENCODE cell types used in this study.

Cell Type	Description	Karyotype	Lineage	Tissue	Replicates
H1-hESC	Embryonic stem cell	Normal	ICM	ESC	4
GM12878	B-lymphocyte	Normal	mesoderm	blood	4
K562	Leukemia	Cancer	mesoderm	blood	3
HeLa-S3	Cervical carcinoma	Cancer	ectoderm	cervix	3
HepG2	Hepatocellular carcinoma	Cancer	endoderm	liver	3
HUVEC	Umbilical vein endothelial cells	Normal	mesoderm	vessel	3
SK-N-SH	Neuroblastoma	Cancer	ectoderm	brain	1
IMR90	Fetal lung fibroblasts	Normal	endoderm	lung	1
A549	Lung carcinoma	Cancer	endoderm	lung	1
MCF-7	Mammary gland adenocarcinoma	Cancer	ectoderm	breast	2
CD20+	CD20+ B cells	Normal	mesoderm	blood	1
CD14+	CD14+ Monocytes	Normal	mesoderm	blood	1
NHEK	Epidermal keratinocytes	Normal	ectoderm	skin	3

Over 2.7 billion sequenced fragments aligned to human reference hg38 with between 23.6% and 46.1% of the fragments in each sample aligning ambiguously to multiple genomic locations. Telescope intersected the aligned fragments with a set of 14,968 manually curated HERV loci belonging to 60 families (see methods) and identified over 27 million fragments that appear to originate from HERV proviruses. Most (80.1%) of these fragments aligned to multiple genomic locations; we used Telescope to reassign ambiguous fragments to the most likely transcript of origin and estimate expression at specific HERV loci.

**Fig 2:**
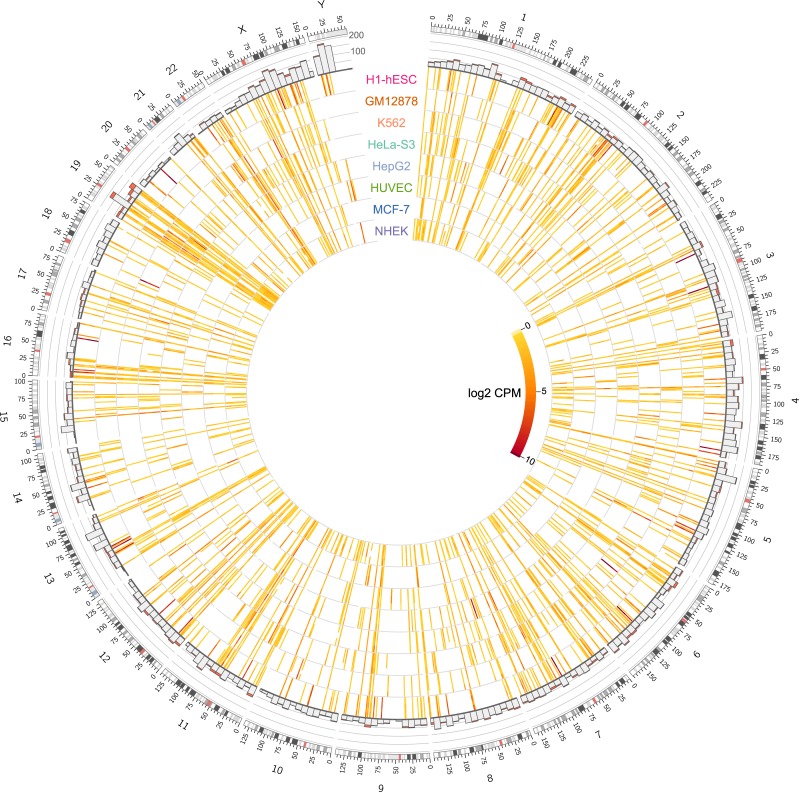
Genome-wide maps of locus-specific HERV expression for 8 ENCODE tier 1 and 2 cell types. The outer track is a bar chart showing the number of HERV loci in 10 Mbp windows, ranging from 0 to 200, with the red part of the bar representing the number of loci that are expressed in one or more cell types. The 8 inner rings show the expression levels (log2 counts per million (CPM)) of 1365 HERV loci that were expressed in at least one of the cell types examined. Moving from the outer ring to the inner ring are replicates for each of the 8 cell types with duplicates: H1-hESC, GM12878, K562, HeLa-S3, HepG2, HUVEC, MCF-7, and NHEK.

We developed genome-wide maps of HERV expression for 8 of the analyzed cell types that had replicates (Table 1, S1 Table), and used CIRCOS[49] to visualize the data (Fig 2). The outer track is a bar chart showing the number of HERV loci in 10 Mbp windows, with the red part of the bar representing the number of loci that are expressed in one or more cell types. The 8 inner rings show the expression levels (log2 counts per million (CPM)) of 1365 HERV loci that were expressed at least one of the cell types examined. Moving from the outer ring to the inner ring are replicates for each of the 8 cell types with replicates: H1-hESC, GM12878, K562, HeLa-S3, HepG2, HUVEC, MCF-7, and NHEK.

We found 1365 HERV loci that were expressed in at least one of the cell types (CPM > 0.5). Not all HERVs were expressed in all cell types, some were widely expressed in all cells, whereas others were only expressed in one or more cell type (Fig 2). There is also a spectrum of differential HERV expression, with some HERVs having significantly higher expression than others. Visual inspection of HERV expression maps suggest that there are certain regions of the genome that have minimal HERV expression, while other regions appear dense in HERV expression (Fig 2). The genomic context of HERV expression can also be inspected more closely in areas of interest, i.e. chromosome 19 (S2 Fig) and chromosome 6 (S3 Fig).

### HERV locus-specific analysis

**Fig 3:**
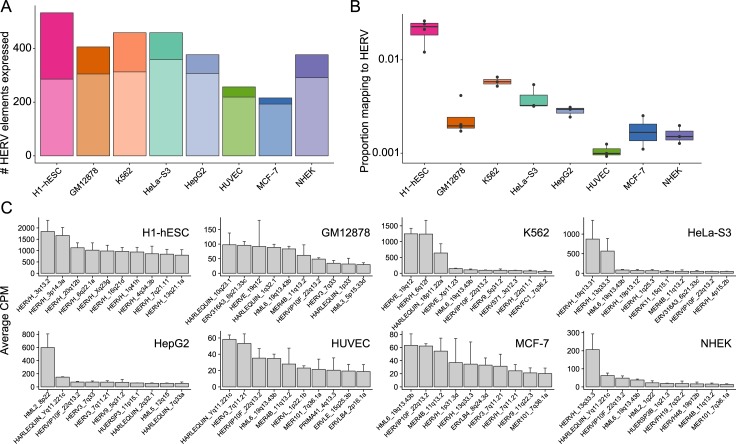
Overall HERV expression patterns. (A) Number of HERV elements that are expressed for each cell type; expressed loci have CPM > 0.5 in the majority of replicates. The darker section of the bar corresponds to expressed loci that are unique to cell type, while the lighter part is expressed in other cell types. (B) The proportion of mapped RNA-seq fragments that are generated from HERV transcripts in each of eight replicated cell types. Each point is one replicate; boxplot shows the median and first and third quartiles. (C) Top 10 most highly expressed loci for each cell type. Height of the bar is average CPM of all replicates with error bars representing the standard error calculated from replicates CPM values.

**Fig 4:**
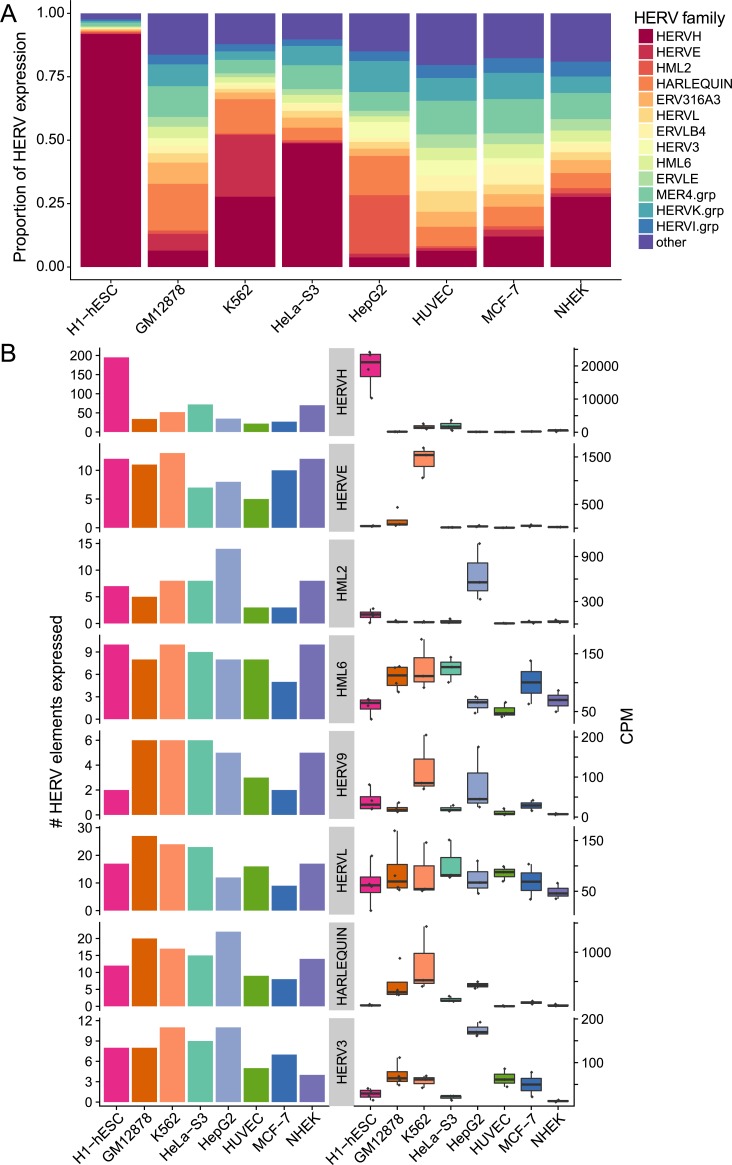
Family-level HERV expression profiles using Telescope. Family-level HERV expression profiles were computed from locus-specific profiles (generated by Telescope) by summing expression across all locations within each subfamily. (A) The proportion of fragments assigned to each HERV subfamily relative to the total amount of HERV expression. Families that account for at least 5% of total HERV expression in at least one cell type are shown, with the remaining families in “other”. (B) Number of expressed HERV loci (left) and fragment counts per million mapped fragments (CPM, right) for selected HERV families. Boxplots for each family were constructed using the average CPM for each expressed locus, with a dark line representing the median of all loci and the box borders representing the 1^st^ and 3^rd^ quartiles. Outlying loci that are greater than 1.5 times the interquartile range from the border of the box are plotted as individual points.

To ascertain global, subfamily and locus level specific HERV expression, we assessed the number of HERVs expressed in each cell type. All cell types expressed HERVs; the number of expressed loci ranged from 216 (in MCF-7), to 533 (H1-hESC) (Fig 3A). The number and proportion of cell type specific locations (expressed in only one cell) differed among cell types. Nearly half (46.3%) of locations expressed in H1-hESC were not expressed in any other cell type, while 89.3% of locations expressed in MCF-7 were also present in other cell types (Fig 3A). This suggests that regulatory networks are shared among some cell types but not others. We next examined the relative contribution of HERV families to overall HERV transcription and found that different cell types could be characterized by enrichment for different HERV families. For example, HERVH accounted for 91.8% of the transcriptomic output in H1-hESC cells, while HERVE was dominant in K562 cells (24.4%) (Fig 4A). Other families, such as HERVL, were evenly distributed across cell types, both in number of expressed locations and in expression levels (Fig 4B). Resolving the most highly expressed locations in each cell type at a locus specific level shows that the distribution of expression varies among cell types. (Fig 3C). For example, HepG2 is characterized by high expression from a single locus, while H1-hESC has many locations that are activated.

### HERV expression profiles generated by Telescope are cell type specific

**Fig 5:**
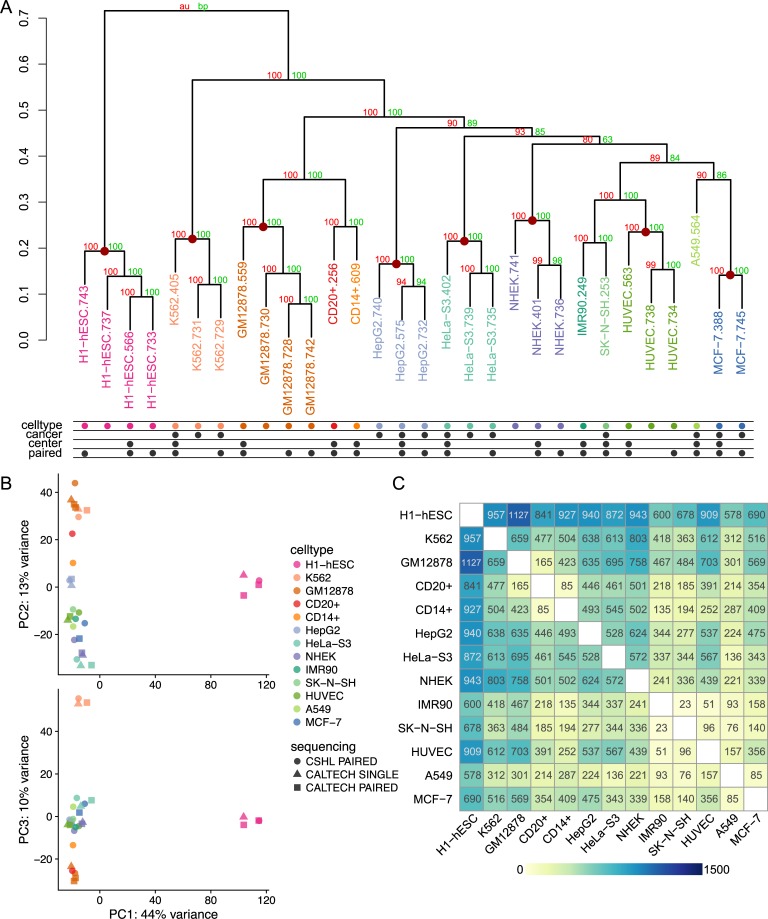
Cell type characterization based on HERV expression profiles using unsupervised learning and linear models. Unsupervised learning and linear modeling were used to identify patterns in HERV expression profiles generated by Telescope for 30 polyA RNA-seq datasets from 13 cell types. (A) Similarities among normalized expression profiles were explored using hierarchical cluster analysis. Supporting p-values were based on 1000 multiscale bootstrap replicates and calculated using Approximately Unbiased (AU, red) and Bootstrap probability (BP, green) approaches. Red dots are placed on nodes that exclusively cluster together all replicates for a cell type. (B) Principal component analysis (PCA) of normalized expression profiles. The first component accounts for 44% of the variance in the data, and is plotted against component 2 and 3, which account for 13% and 10% of the variance, respectively. (C) Heatmap of the number of HERV elements found to be significantly differentially expressed (DE) among each pair of cell types. Significance was determined using cutoffs for the false discovery rate (FDR < 0.1) and log2 fold change (abs(LFC) > 1.0). Yellow indicates low numbers of differentially expressed elements, while blue indicates high numbers.

Previous work has suggested that estimates of HERV expression are highly sensitive to sequencing technology used, and differences due to sequencing technology can obscure biological differences due to cell type[40]. Since aligning shorter fragments (i.e. single-end reads) tends to produce more ambiguously mapping fragments compared to longer fragments, we hypothesized that Telescope (which resolves ambiguity) would create HERV expression profiles that are robust to differences in sequencing technology. Hierarchical clustering of all 30 polyA RNA-seq HERV profiles shows that replicates from the same cell type cluster most closely with other samples from the same cell type, regardless of the sequencing technology used (Fig 5A). Clusters for all cell types had significant support using multiscale bootstrap resampling (approximately unbiased (AU) > 95%). Principal component analysis (PCA) also indicates that cell type, not sequencing technology, is associated with the strongest differences among expression profiles. The first principal component, accounting for 44% of the total variance in the data, separates H1-hESC samples from all other samples (Fig 5B). The second and third components further separate the samples into the other 12 cell types, and capture 13% and 10% of the total variance, respectively. Interestingly, the second component separates blood-derived cell types (K562, GM12878, CD20+ and CD14+) from the other cell types, suggesting that cells derived from the same tissue may share similarities in HERV expression profiles.

We further explored differences among cell types using differential expression (DE) analysis. Pairwise contrasts between cell types were performed to determine the number of significant DE loci (FDR < 0.1, abs(LFC) > 1.0) (Fig 5C). As found in the unsupervised analysis, HERV expression in H1-hESC was drastically different from other cell types, with between 578 and 1127 significantly DE loci.

Finally, we asked whether other existing approaches for TE quantification would be sufficient to identify cell type specific signal in the data or whether these approaches would be sensitive to other variables. We analyzed the ENCODE datasets using default parameters for five other approaches, including best counts, unique counts, TEtranscripts, RepEnrich, and SalmonTE. Hierarchical clustering of the resulting expression profiles reveal that cell types clusters are only recovered using unique counts and Telescope (S3 Fig), though unique counts tended to have less support for clusters. In contrast, clustering with the other four approaches did not recover all cell type clusters; 7 out of 8 cell types clustered together when using best counts expression profiles, 5 cell types were recovered with TEtranscripts and RepEnrich, and only 1 cell type cluster was recovered with SalmonTE profiles (S4 Fig). Interestingly, clustering of the SalmonTE expression profiles revealed 5 samples that did not cluster with their respective cell types, but instead clustered with other single-end datasets (S4 Fig).

### Statistical performance of TE quantification methods

In order to examine the sensitivity and biases of computational approaches for quantifying TE expression, we designed simulation experiments with known expression values. Earlier studies have suggested that the HERV-K(HML-2) subfamily (hereafter referred to as HML-2) is expressed in human tissue and may be relevant to human health[8,10,50,51]. Furthermore, its relatively few subfamily members (~90 distinct genomic loci[48]) and high nucleotide identity make HML-2 a good model for studying TE expression. Here, we report on the performance of each method to detect locus-specific expression of HML-2 by simulating RNA-seq fragments with sequencing error. We simulated 25 independent RNA-seq datasets (see methods) and analyzed each using 7 TE quantification approaches: 1) unique counts, 2) best counts, 3) RepEnrich, 4) TEtranscripts, 5) RSEM, 6) SalmonTE, and 7) Telescope. To ensure equal comparisons, all approaches use the same annotation (S1 File), and modifications to the annotation were made to allow locus-specific quantification (instead of family-level quantification) for RepEnrich, TEtranscripts, and SalmonTE.

**Fig 6:**
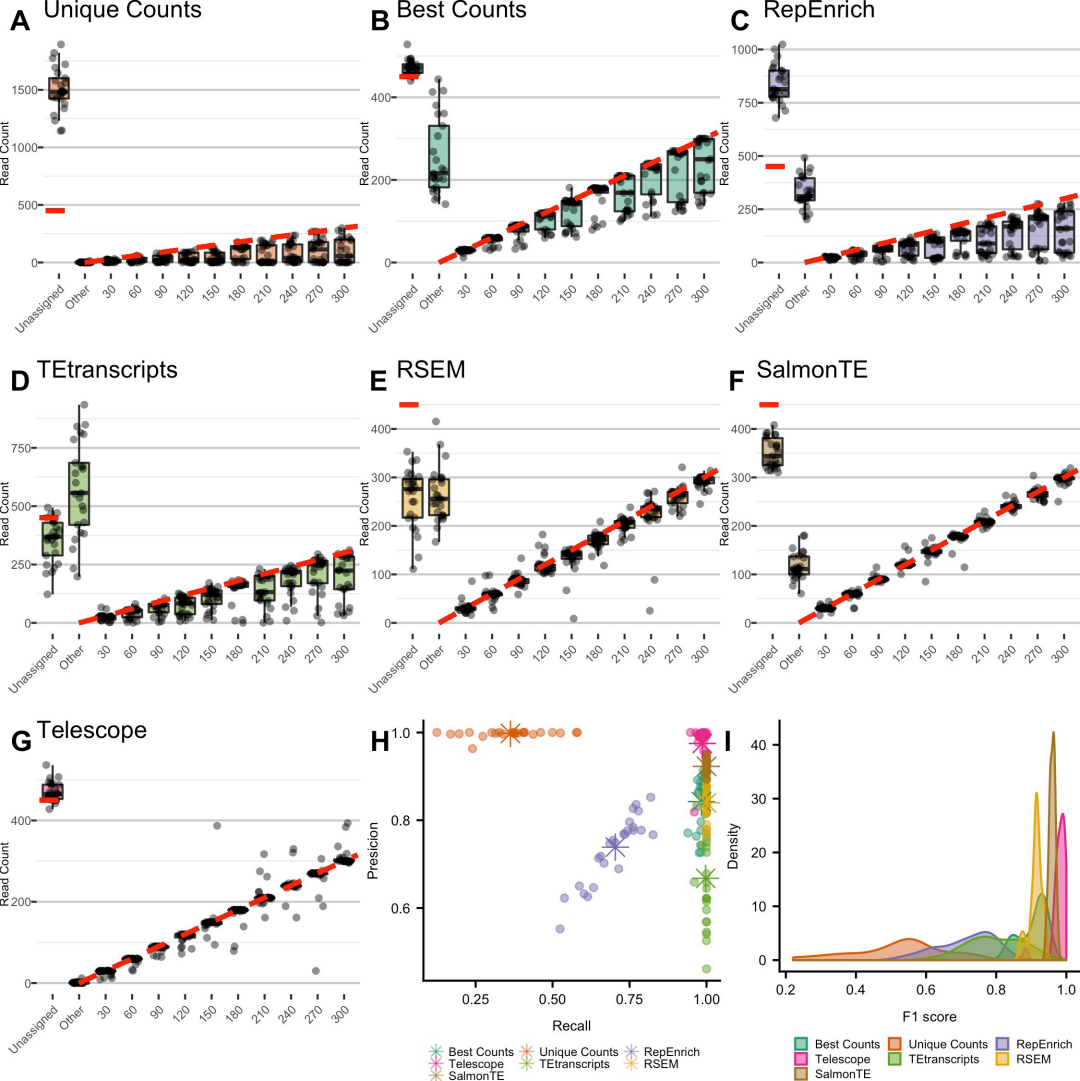
Comparison of performance results for TE quantification approaches. 25 RNA-seq samples were simulated, each sample consisted of 10 randomly chosen HML-2 loci with simulated counts equal to 30, 60, 90, 120, 150, 180, 210, 240, 270, and 300. Each point represents the final count from one simulation, with the expected (simulated) expression value on the x-axis. Reads that were not assigned to one of the 10 expressed loci were categorized as “Unassigned” if the read did not map to any loci in the annotation, and “Other” if assigned to an annotated locus that was not expressed; these categories are also shown on the x-axis. A boxplot showing the median and quartiles is shown for each category, and the expected expression value is represented with a red dashed line. Approaches tested: (A) unique counts, (B) best counts, (C) RepEnrich, (D) TEtranscripts, (E) RSEM, (F) SalmonTE, and (G) Telescope. The precision and recall for each sample simulated as well as the mean of both are shown for all methods (H), and F1-score calculation (I).

For all simulations, we plotted the final counts estimated by each approach compared to the expected read count (Fig 6A–6G). We calculated the precision and recall across all loci and simulations (Fig 6H) and represented the overall accuracy of the approach using the F1 score (Fig 6I). Five out of seven approaches were highly sensitive, with true positive rates above 95% in most simulations. The two exceptions were RepEnrich and unique counts, which both tend to discard many more reads than expected (“Unassigned”, Fig 6A and 6C). The unique counts approach consistently underestimated expression levels with ~40% of all estimates (96 out of 250) missing at least 50% of the true expression (Fig 6A). One striking example of this underestimation was for HML2_8p21e; this locus did not generate any fragment that could be uniquely mapped, thus was never detected by this approach.

Performance of the other five approaches differed primarily in the type and magnitude of misclassification errors. False positives occur when reads are incorrectly assigned to annotated loci that are not expressed, resulting in incorrect detection of unexpressed HERV loci. Best counts had a high false positive rate; on average, 12.1% of fragments were incorrectly assigned to unexpressed loci resulting in false detection of unexpressed loci in all simulations (“Other”, Fig 6B). Similarly, the average proportion of reads assigned to unexpressed HERVs is greater than 5% for TEtranscripts, RSEM, and SalmonTE (“Other”, Fig 6D–6F) but is less than 0.1% for Telescope (“Other”, Fig 6G). On the other hand, false negatives occur when reads originating from non-TE regions are assigned to TEs. Since we expect non-TE reads to be unassigned, the number of false negatives can be measured by the difference between the expected number of non-TE reads and the final number of unassigned reads (“Unassigned”, Fig 6). Best counts and Telescope both tend to correctly discard non-TE reads (“Unassigned”, Fig 6B and 6G), while TEtranscripts, RSEM, and SalmonTE tend to incorrectly assign these reads to annotated TEs (“Unassigned”, Fig 6D–6F). We suspect that the model implemented in TEtranscripts attempts to assign all fragments to annotated transcripts, as there is no category for unannotated regions in their model. For RSEM and SalmonTE, this error may be due to the restricted sequence space used to classify the reads. As these methods are mapping to the transcriptome, the true originating sequence is absent from the index and fragments are forced to map to similar, yet incorrect, sequences. This error could be avoided by developing more complete TE annotations or including additional loci that share sequence similarity with TEs of interest.

Of all methods considered here, Telescope had the highest rate of precision and recall from all other counting methods tested (Fig 6H and 6I). In contrast to the best counts approach (Fig 6I), Telescope assigned only 20 fragments to genomic annotations that were not expressed, while 6061 fragments were assigned incorrectly by best counts. The overall accuracy of Telescope estimates from true expression levels, as measured by F1-score, was the highest of all approaches (Fig 6I). These simulation results demonstrate that Telescope resolves ambiguously aligned fragments and produces unbiased estimates of TE expression that are robust to sequencing error.

## Discussion

Transposable elements represent a major biochemically active group of transcripts that are increasingly recognized as important regulators in complex biological systems and disease. However, difficulties in identifying and quantifying these elements has led to TEs being largely ignored in the literature. Here we present Telescope, a novel software package that can be used to mine new or existing RNA-seq datasets to accurately quantify the expression of TEs. The key advantage of our approach is the capability to localize TE expression to an exact chromosomal location.

Based on our analysis of 13 ENCODE cell types, we have identified 1365 individual HERV loci that are expressed in one or more cell types and generated genomic maps that showing cell type specific HERV expression profiles. The ability to quantify expression at specific loci demonstrates that regulation of HERV expression occurs at the locus level (in addition to subfamily-level regulation), as different expression patterns are observed for loci within the same subfamily. For example, our results confirm previous studies identifying HERVH upregulation in embryonic stem cells [9,39,52] and add to this finding by identifying the precise location of HERVH insertions that produce the most transcripts. This high level of resolution for TE expression enables further investigation into the local genomic context, epigenetic regulation, and coding potential of expressed loci.

An earlier study investigating HERV expression using the same datasets found strong differences in estimated HERV expression profiles depending on the sequencing technology used (paired or single end)[40]. Using Telescope, we did not find this same bias; instead, replicates of the same cell type clustered together, while most variance in the data was among cell types. Four of the other TE quantification approaches tested did not appear biased with respect to sequencing technology, while one (SalmonTE) appeared to separate single-end from paired-end samples. We suspect that this is a result of SalmonTEs pseudoalignment approach, as more ambiguous assignments can occur if pairing information is not considered. Other types of bias, such as fragment bias, have been identified in RNA-seq data[53] and may influence expression estimates in Telescope and other programs. We expect future versions of our software to implement corrections for these biases.

Our simulations show that Telescope is highly sensitive and has low type I and II error rates. Unique counts, a heuristic that is commonly chosen for its unambiguous assignments, was shown to discard much of the data and underestimate true TE expression. Best counts, which is commonly used for convenience, also performed poorly and spuriously identified transcripts that were not expressed. Several software packages, including RepEnrich, TEtranscripts, and SalmonTE, also aim to quantify TE expression, but use a family-level approach that quantifies TE subfamilies instead of individual loci. Our simulations used modified inputs for these approaches that allowed us to compare them to Telescope. Based on our simulation results, we find that our approach achieves high sensitivity while minimizing spurious detections, while all other approaches tend to identify TEs that are not expressed. We conclude that Telescope offers superior accuracy for TE quantification and is the only available software package that quantifies TE expression at single-locus resolution.

Telescope will have widespread utility in other settings. Studies on TE expression have become prominent in studies of embryonic stem cell development[8][9], neural cell plasticity[54,55], oncogenesis[4–7,56,57], psychiatric and neurological disorders[58–60] and autoimmune diseases[61,62]. As the breadth of knowledge on TEs expands, expression profiling of TEs using Telescope will allow scientists to discover unique and collective TE transcripts involved in the biology of complex systems.

## Methods

### Fragment reassignment mixture model

Telescope implements a generative model of RNA-seq relating the probability of observing a sequenced fragment to the proportions of fragments originating from each transcript. Formally, let *F* = [*f*_1_,*f*_2_,…,*f*_*N*_] be the set of *N* observed sequencing fragments. We assume these fragments originate from *K* annotated transcripts in the transcriptome *T* = [*t*_0_,*t*_1_,…,*t*_*K*_]. In practice, annotations fail to identify all possible transcripts that generate fragments, thus we include an additional category, *t*_0_, for fragments that cannot be assigned to annotated transcripts. Let *G* = [*G*_1_,*G*_2_,…,*G*_*N*_] represent the true generating transcripts for *F*, where *G*_*i*_∈*T* and *G*_*i*_ = *t*_*j*_ if *f*_*i*_ originates from *t*_*j*_. Since the process of generating *F* from *T* cannot be directly observed, the true generating transcripts *G* are considered to be “missing” data. The objective of our model is to estimate the proportions of *T* by learning the generating transcripts of *F*.

The alignment stage identifies one or more possible alignments for each fragment, along with corresponding alignment scores. Telescope uses the alignment score generated by the aligner and reported in the AS tag[63]. This is typically calculated by adding scores and penalties for each position in the alignment; a higher alignment score indicates a better alignment. Let *q*_*i*_ = [*q*_*i*0_,*q*_*i*1_,…,*q*_*iK*_] be the set of mapping qualities for fragment *f*_*i*_, where *q*_*ij*_ = Pr(*f*_*i*_|*G*_*i*_ = *t*_*j*_) represents the conditional probability of observing *f*_*i*_ assuming it was generated from *t*_*j*_; we calculate this by scaling the raw alignment score by the maximum alignment score observed for the data. We write the likelihood of observing uniquely aligned fragment *f*_u_ as a function of the conditional probabilities *q*_u_ and the relative expression of each transcript for all possible generating transcripts *G*_u_

$$\mathrm{Pr}\left(f_{u}|\pi ,q_{u}\right)=\sum_{j=0}^{K}\pi_{j}q_{uj}$$

where ***π*** = [*π*_0_,*π*_1_,…,*π*_*K*_] represents the fraction of observed fragments originating from each transcript. Note that *q*_u*j*_ = 0 for all transcripts that are not aligned by *f*_u_. For non-unique fragments, we introduce an additional parameter in the above likelihood to reweight each ambiguous alignment among the set of possible alignments. The probability of observing ambiguous fragment *f*_a_ is given by

$$\mathrm{Pr}\left(f_{a}|\pi ,\theta ,q_{a}\right)=\sum_{j=0}^{K}\pi_{j}\theta_{j}q_{aj}$$

where ***θ*** = [*θ*_0_,*θ*_1_,…,*θ*_*K*_] is a reassignment parameter representing the fraction of non-unique reads generated by each transcript.

Using these probabilities of observing ambiguous and unique fragments, we formulate a mixture model describing the likelihood of the data given parameters ***π*** and ***θ***. The *K* mixture weights in the model are given by ***π***, the proportion of all fragments originating from each transcript. To account for uncertainty in the initial fragment assignments, let *x*_*i*_ = [*x*_*i*0_,*x*_*i*1_,…,*x*_*iK*_] be a set of partial assignment (or membership) weights for fragment *f*_*i*_, where $\sum_{j=0}^{K}x_{ij}=1$ and *x*_*ij*_ = 0 if *f*_*i*_ does not align to *t*_*j*_. We assume that *x*_*i*_ is distributed according to a multinomial distribution with success probability ***π*.** Intuitively, *x*_*ij*_ represents our confidence that *f*_*i*_ was generated by transcript *t*_*j*_. In order to simplify our notation, we introduce an indicator variable ***y*** = [*y*_1_,*y*_2_,…,*y*_*N*_] where *y*_*i*_ = 1 if *f*_*i*_ is ambiguously aligned and *y*_*i*_ = 0 otherwise. The complete data likelihood is

$$L(\pi ,\theta |x,q,y)\propto \prod_{i=1}^{N}\prod_{j=0}^{K}{\left[\pi_{j}\theta_{j}^{y_{i}}q_{ij}\right]}^{x_{ij}}$$

### Parameter estimation and fragment reassignment by EM

Telescope iteratively optimizes the likelihood function using an expectation-maximization algorithm[64]. First, the parameters ***π*** and ***θ*** are initialized by assigning equal weight to all transcripts. In the expectation step, we compute the expected values of *x*_*i*_ under current estimates of the model parameters. The expectation is given by the posterior probability of *x*_*i*_:

$$E[x_{ij}]=\frac{\pi_{j}\theta_{j}^{y_{i}}\;q_{ij}}{\sum_{k=0}^{K}\pi_{k}\theta_{k}^{y_{i}}\;q_{ik}}$$

In the M-step we calculate the maximum a posteriori (MAP) estimates for ***π*** and ***θ***

$$\hat{\pi_{j}}=\frac{\sum_{i=1}^{N}E[x_{ij}]+a_{j}}{M+\sum_{k=0}^{K}a_{k}}\;and\;\hat{\theta_{j}}=\frac{\sum_{i=1}^{N}E[x_{ij}]y_{i}+b_{j}}{\sum_{i=1}^{N}y_{i}+\sum_{k=0}^{K}b_{k}}$$

Where $M=\sum_{j=0}^{K}\sum_{i=1}^{N}E[x_{ij}]$ and *a*_*j*_ and *b*_*j*_ are prior information for transcript *t*_*j*_. Intuitively, these priors are equivalent to adding unique or ambiguous fragments to *t*_*j*_. As currently implemented, the user may provide a prior value for either parameter that is distributed equally among all transcripts. We have found that providing an informative prior for the *b*_*j*_ (--theta_prior) is recommended given the repeat content of the human genome, since large values for this parameter prevents convergence to boundary values. Convergence of EM algorithms to local maxima has been shown by Wu[65], and is achieved when the absolute change in parameter estimates is less than a user defined level, typically *ϵ*<0.001.

### HERV annotations

A Telescope analysis requires an annotation that defines the transcriptional unit of each TE to be quantified. For HERV proviruses, the prototypical transcriptional unit contains an internal protein-coding region flanked by LTR regulatory regions. Existing annotations, such as those identified by RepeatMasker[33] (using the RepBase database[32]) or Dfam[66] identify sequence regions belonging to TE families but do not seek to annotate transcriptional units. Both databases represent the internal region and corresponding LTRs using separate models, and the regions identified are sometimes discontinuous. Thus, a HERV transcriptional unit is likely to appear as a collection of nearby annotations from the same HERV subfamily.

Transcriptional units for HERV proviruses were defined by combining RepeatMasker annotations belonging to the same HERV subfamily that are located in adjacent or nearby genomic regions. Briefly, repeat families belonging to the same HERV subfamily (internal region plus flanking LTRs) were identified using the RepBase database[32]. RepeatMasker annotations for each repeat subfamily were downloaded using the UCSC table browser[67] and converted to GTF format, merging nearby annotations from the same repeat subfamily. Next, LTRs found flanking internal regions were identified and grouped using BEDtools[68]. HERV transcriptional units containing internal regions were assembled using custom python scripts. Each putative locus was categorized according to provirus organization; loci that did not conform to expected HERV organization or conflicted with other loci were visually inspected using IGV[69] and manually curated. As validation, we compared our annotations to the HERV-K(HML-2) annotations published by Subramanian et al.[48]; the two annotations were concordant. Final annotations were output as GTF (S1 File); all annotations, scripts, and supporting documentation are available at https://github.com/mlbendall/telescope_annotation_db.

### HERV expression analysis of ENCODE datasets

We identified 30 ENCODE datasets with available whole-cell bulk RNA-seq data from tier 1 and 2 common cell types (S1 Table). Sequence data was obtained from SRA and extracted using the parallel-fastq-dump package (https://github.com/rvalieris/parallel-fastq-dump). Adapter trimming, quality trimming, and filtering were performed using Flexbar[70] (version 3.0.3). For Telescope analysis, the trimmed and filtered reads from each run were aligned to human reference genome hg38 using bowtie2[71]. Alignment options were specified to perform a sensitive local alignment search (--very-sensitive-local) with up to 100 alignments reported for each fragment pair (-k 100). The minimum alignment score threshold was chosen so that fragments with approximately 95% or greater sequence identity would be reported (--score-min L,0,1.6). Sequence alignment map (SAM/BAM) files from different runs corresponding to the same sample were concatenated to obtain sample-level BAM files. An annotation of HERV locations in hg38 (S1 File) and the BAM file for each sample were provided as inputs for Telescope. Telescope options included up to 200 iterations of the expectation-maximization algorithm (--max_iter 200) and an informative prior on theta (--theta_prior 200000). The “final counts” column in the Telescope report are used as HERV expression data in subsequent analysis.

ENCODE datasets were also analyzed using five other approaches. Unique and best counts approaches use the same alignment and annotation as above and are included as part of the Telescope output. RepEnrich, TEtranscripts, and SalmonTE were all run according to author instructions, with author-provided annotations and default parameters.

### Differential expression analysis

Library size for each sample is considered to be the total number of fragments that map to the reference genome. Counts per million (CPM) were calculated by dividing the raw count by the library size and multiplying by 1 million. A CPM cutoff of 0.5 was used to identify expressed loci; since the smallest sample considered has more than 20 million fragments, expressed loci are represented by at least 10 observations. Raw counts output by Telescope were used for differential expression analysis. Size factors for normalization were calculated by dividing the library sizes by their geometric mean. Normalization, dispersion estimation, and generalized linear model fitting was performed using DESeq2[72]; the model was specified with cell type as the only covariate. Contrasts were extracted for each pair of cell types; HERVs with an adjusted p-value < 0.1 and log2FoldChange > 1.0 were considered to be differentially expressed.

### Hierarchical clustering

Read counts for clustering were transformed using a variance stabilizing transformation in DESeq2[72]. Hierarchical clustering with multiscale bootstrap resampling was performed on transformed counts using correlation distance and UPGMA clustering implemented in pvclust[73]. Uncertainty in hierarchical cluster analysis was assessed by calculating two p-values for each cluster that range from 0 to 1, with 1 indicating strong support for the cluster. The bootstrap probability (BP) is calculated by normal bootstrap resampling and approximately unbiased (AU) probability is computed by multiscale bootstrap resampling[74].

### Simulated HML-2 expression data

For the simulation study, we simulated 25 independent RNA-seq datasets with 2100 paired-end fragments each. For each dataset, we randomly selected 13 loci to be expressed, including 10 HML-2 proviruses and three “non-TE” loci. HML-2 proviruses were selected from 92 HML-2 loci present in our annotation; non-TE loci were selected from a set of 968 unannotated genomic regions that share sequence similarity with the HML-2 subfamily (S2 File). Non-TE loci are included to examine the type II error rate of the approaches; assigning non-TE fragments to HML-2 loci is considered a false negative. Expression levels for the 10 HML-2 loci in each dataset were randomly chosen, ranging from 30 to 300 fragments per locus. Each of the three non-TE loci were expressed at 150 fragments each. Using this expression pattern, we simulated sequencing fragments with the Bioconductor package for RNA-seq simulation, Polyester[75]. All simulations used the parameters of read length: 75 bp; average fragment size: 250; fragment size standard deviation: 25; and an Illumina error model with an error rate of 5e-3.

### Analysis of simulation data with TE quantification approaches

Each simulation dataset was analyzed using 7 TE quantification approaches: 1) unique counts, 2) best counts, 3) RepEnrich, 4) TEtranscripts, 5) RSEM, 6) SalmonTE, and 7) Telescope. To ensure a fair comparison among approaches, the same annotation (S1 File) was used as input for all approaches. Note that the HML-2 loci used for simulation are contained in this annotation, but the non-TE loci are absent. For RepEnrich, TEtranscripts, and SalmonTE, the locus identifier was used in place of the family name in order to generate locus-specific estimates. Aside from these changes, each program was run as suggested by the authors. Unique counts was implemented by aligning reads with bowtie2, allowing for multi-mapped reads (-k 100 --very-sensitive-local --score-min L,0,1.6) and filtering reads with multiple alignments. The same bowtie2 parameters were used for best counts without specifying -k (--very-sensitive-local --score-min L,0,1.6).

The five software packages include final read counts as part of the output. Read counts for the unique counts and best counts approaches were obtained using htseq-count[76]. After mapping and counting the reads for each annotated HERV, reads can be divided in two categories, depending their origin, HML-2 reads or non-TE reads. Those reads can then be correctly or incorrectly mapped, depending of the outcome of the counting method, leading to 4 different categories: a) reads assigned to HML-2 correctly (True Positive) b) reads assigned to HML-2 incorrectly (False Positive) c) reads not assigned correctly (True Negative) d) reads not assigned incorrectly (False Negative). All classifications were made based on counts and not fragment assignments, as several approaches do not provide final fragment assignments. The classifications were used for recall and precision calculations.

### Implementation

Telescope is implemented in Python, is available as an open-source program under the MIT license, and has been developed and tested on Linux and MacOS. The software package and test data can be found at https://github.com/mlbendall/telescope. We recommend installing Telescope and its dependencies using the bioconda package manager[77].

A complete snakemake[78] pipeline for reproducing the ENCODE analysis is available from https://github.com/mlbendall/TelescopeEncode. Scripts for reproducing the simulations are available from https://github.com/LIniguez/Telescope_simulations. A tutorial for running the single-locus analysis is available from https://github.com/mlbendall/telescope_demo.

## Supporting information

S1 Fig Telescope resolves alignment ambiguity and enables single-locus expression estimation. Visualization of simulated fragment alignments to three selected HML-2 loci. Simulated fragments were generated from HML2_1q22. Proviruses HML2_5q33.3 and HML2_11q22.1 were chosen as examples because they are closely related to HML2_1q22 and have high numbers of initially ambiguous mappings. The top track shows alignments found using bowtie2 while allowing for multimapping (-k 100); bottom track shows the alignments after being reassigned using Telescope. Fragments shown in gold represent unique mapping locations, dark blue fragments represent a best alignment out of several possible alignments, and light blue fragments represent alignments with suboptimal alignment scores. Alignments shown in white (bottom track only) are included to indicate alignments that were present in the initial alignment but were reassigned to HML2_1q22 by Telescope.
(PDF)

S2 Fig HERV expression map for chromosome 19 positions 53,000,000–59,000,000. Outer track is a plot of RefSeq gene locations, with genes containing zinc-finger domains in green.
(EPS)

S3 Fig HERV expression map for chromosome 6 positions 25,000,000–37,000,000. Outer track is a plot of RefSeq gene locations, with genes containing zinc-finger domains in green, human leukocyte antigen (HLA) genes in blue, and histone genes in purple.
(EPS)

S4 Fig Hierarchical clustering of HERV expression profiles estimated using other approaches. Expression values were estimated using each approach using author provided annotations and default arguments. Resulting counts were normalized by library size, transformed, and clustered using pvclust[73]. Supporting p-values were based on 1000 multiscale bootstrap replicates and calculated using Approximately Unbiased (AU, red) and Bootstrap probability (BP, green) approaches. Red dots are placed on nodes that exclusively cluster together all replicates for a cell type.
(EPS)

S1 Table ENCODE datasets profiled using Telescope. Information about each sample analyzed, including ENCODE experiment ID, GEO sample accession, and SRA run accessions. The first column contains the display name of each sample used in Fig 5 and S4 Fig.
(XLSX)

S1 File Annotation of HERV elements from 60 subfamilies in reference genome hg38. Annotation contains 14,968 HERV loci in GTF format. The “locus” attribute is used to identify features belonging to the same locus. “Exon” features are regions matching to transposable element models, while “gene” features span the full locus, including insertions.
(GTF)

S2 File Annotation of non-TE loci for simulation. Annotation contains a set of 968 unannotated genomic regions that share sequence similarity with the HML-2 subfamily.
(BED)

S3 File Comparison of HERV annotation to previously described HML-2 elements. The HERV annotation created for this study was compared to previously described HML-2 proviruses. Tables 1 and 2 from Subramanian et al.[48] were lifted over to hg38 and visualized using IGV. The annotations were mostly concordant. Previously identified loci that are not found in our annotation include two solo LTRs (10p12.1 and 12q13.2), one polymorphic locus (19p12b), and one locus that did not satisfy the minimum length threshold (16p13.3).
(PDF)
