# Supplementary material for: Telescope: Characterization of the retrotranscriptome by accurate estimation of transposable element expression
Source: PLoS Comput Biol. 2019 Sep 30;15(9):e1006453. doi: 10.1371/journal.pcbi.1006453 (PMC6786656; doi:10.1371/journal.pcbi.1006453)
Supplement: S1 Fig — Visualization of simulated fragment alignments to three selected HML-2 loci. Simulated fragments were generated from HML2_1q22. Proviruses HML2_5q33.3 and HML2_11q22.1 were chosen as examples because they are closely related to HML2_1q22 and have high numbers of initially ambiguous mappings. The top track shows alignments found using bowtie2 while allowing for multimapping (-k 100); bottom track shows the alignments after being reassigned using Telescope. Fragments shown in gold represent unique mapping locations, dark blue fragments represent a best alignment out of several possible alignments, and light blue fragments represent alignments with suboptimal alignment scores. Alignments shown in white (bottom track only) are included to indicate alignments that were present in the initial alignment but were reassigned to HML2_1q22 by Telescope. (PDF) [file pcbi.1006453.s001.pdf]

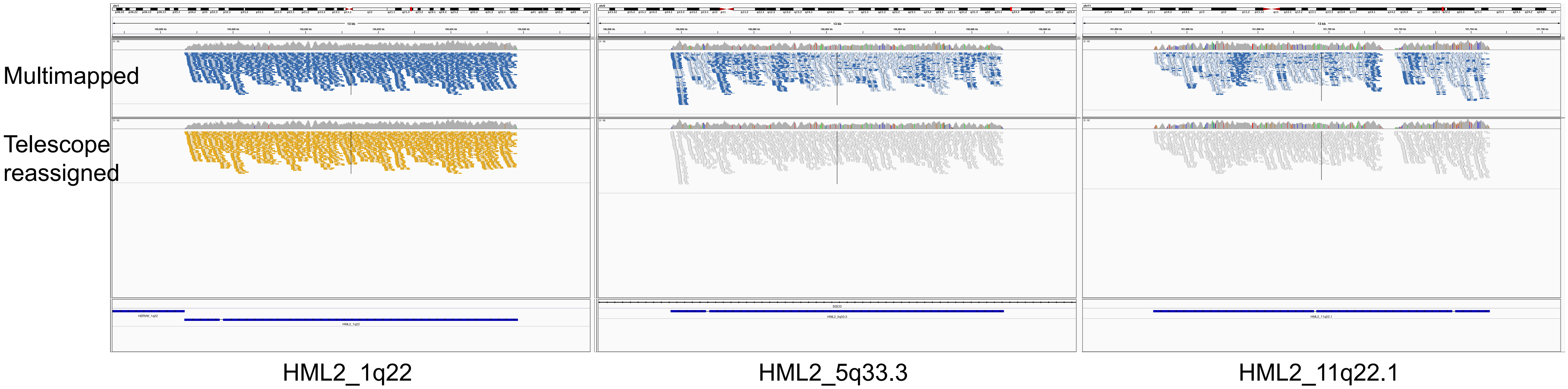


**S1 Fig. Telescope resolves alignment ambiguity and enables single-locus expression estimation.** Visualization of simulated fragment alignments to three selected HML-2 loci. Simulated fragments were generated from HML2_1q22. Proviruses HML2_5q33.3 and HML2_11q22.1 were chosen as examples because they are closely related to HML2_1q22 and have high numbers of initially ambiguous mappings. The top track shows alignments found using bowtie2 while allowing for multimapping (-k 100); bottom track shows the alignments after being reassigned using Telescope. Fragments shown in gold represent unique mapping locations, dark blue fragments represent a best alignment out of several possible alignments, and light blue fragments represent alignments with suboptimal alignment scores. Alignments shown in white (bottom track only) are included to indicate alignments that were present in the initial alignment but were reassigned by Telescope.
