# Supplementary material for: Telescope: Characterization of the retrotranscriptome by accurate estimation of transposable element expression
Source: PLoS Comput Biol. 2019 Sep 30;15(9):e1006453. doi: 10.1371/journal.pcbi.1006453 (PMC6786656; doi:10.1371/journal.pcbi.1006453)
Supplement: S3 File — The HERV annotation created for this study was compared to previously described HML-2 proviruses. Tables 1 and 2 from Subramanian et al.[48] were lifted over to hg38 and visualized using IGV. The annotations were mostly concordant. Previously identified loci that are not found in our annotation include two solo LTRs (10p12.1 and 12q13.2), one polymorphic locus (19p12b), and one locus that did not satisfy the minimum length threshold (16p13.3). (PDF) [file pcbi.1006453.s008.pdf]

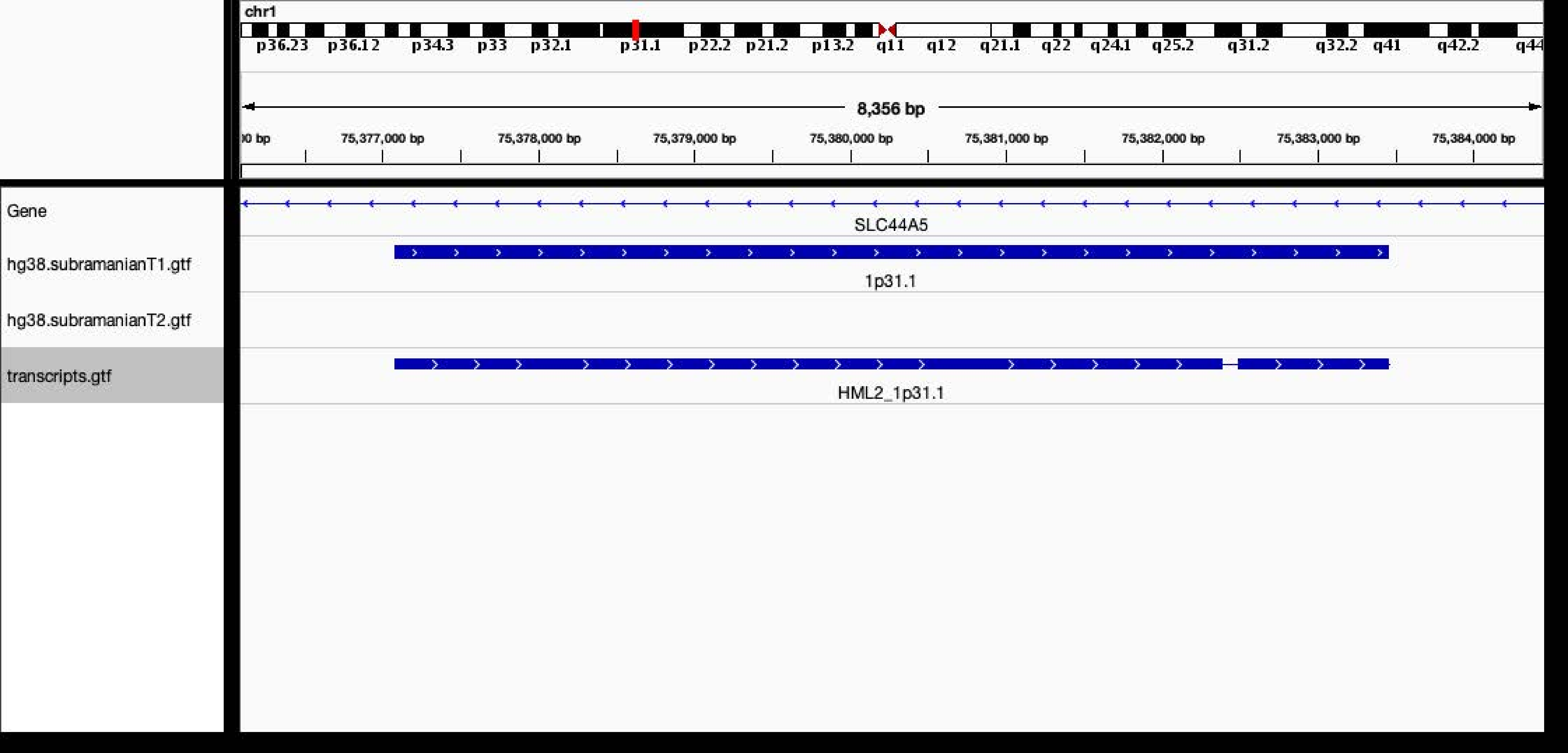
chr1
p36.23
p36.12
p34.3
p33
p32.1
p31.1
p22.2
p21.2
p13.2
q11
q12
q21.1
q22
q24.1
q25.2
q31.2
q32.2
q41
q42.2
8,356 bp
75,377,000 bp
75,378,000 bp
75,379,000 bp
75,380,000 bp
75,381,000 bp
75,382,000 bp
75,383,000 bp
75,384,000 bp
Gene
SLC44A5
hg38.subramanianT1.gtf
1p31.1
hg38.subramanianT2.gtf
transcripts.gtf
HML2_1p31.1

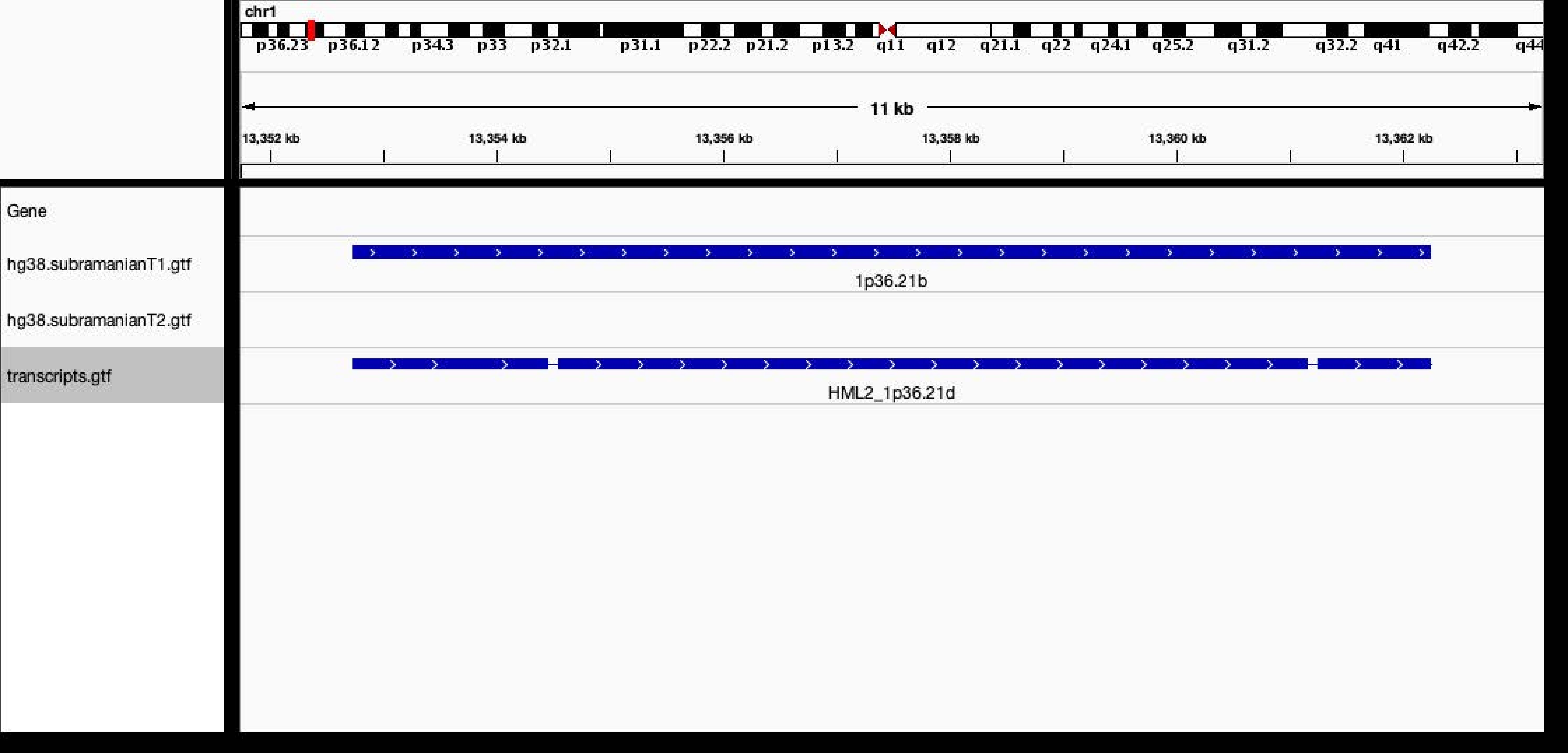

chr1
p36.23
p36.12
p34.3
p33
p32.1
p31.1
p22.2
p21.2
p13.2
q11
q12
q21.1
q22
q24.1
q25.2
q31.2
q32.2
q41
q42.2
11 kb
13,352 kb
13,354 kb
13,356 kb
13,358 kb
13,360 kb
13,362 kb
Gene
hg38.subramanianT1.gtf
1p36.21b
hg38.subramanianT2.gtf
transcripts.gtf
HML2_1p36.21d

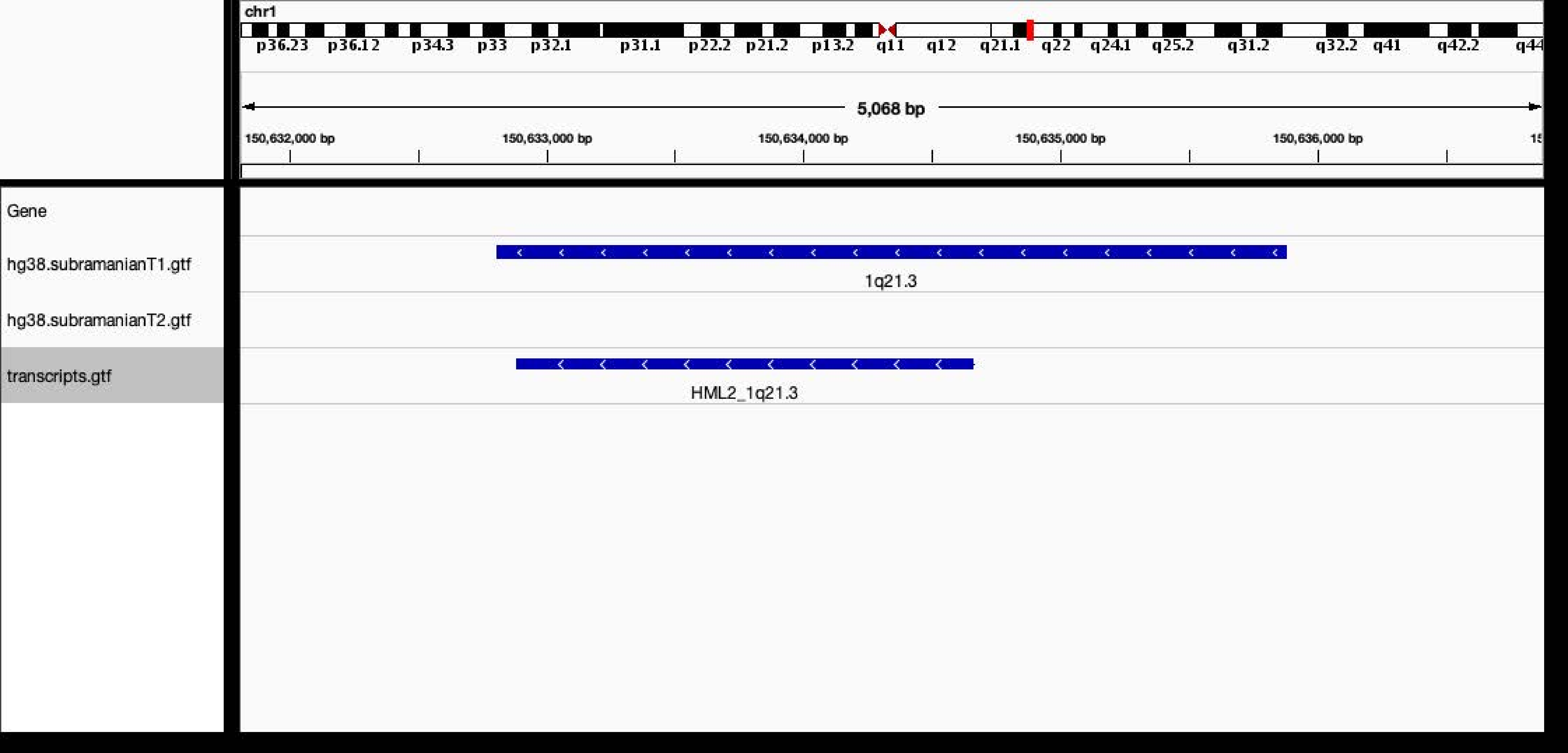

chr1
p36.23 p36.12 p34.3 p33 p32.1 p31.1 p22.2 p21.2 p13.2 q11 q12 q21.1 q22 q24.1 q25.2 q31.2 q32.2 q41 q42.2
5,068 bp
150,632,000 bp
150,633,000 bp
150,634,000 bp
150,635,000 bp
150,636,000 bp
Gene
hg38.subramanianT1.gtf
1q21.3
hg38.subramanianT2.gtf
transcripts.gtf
HML2_1q21.3

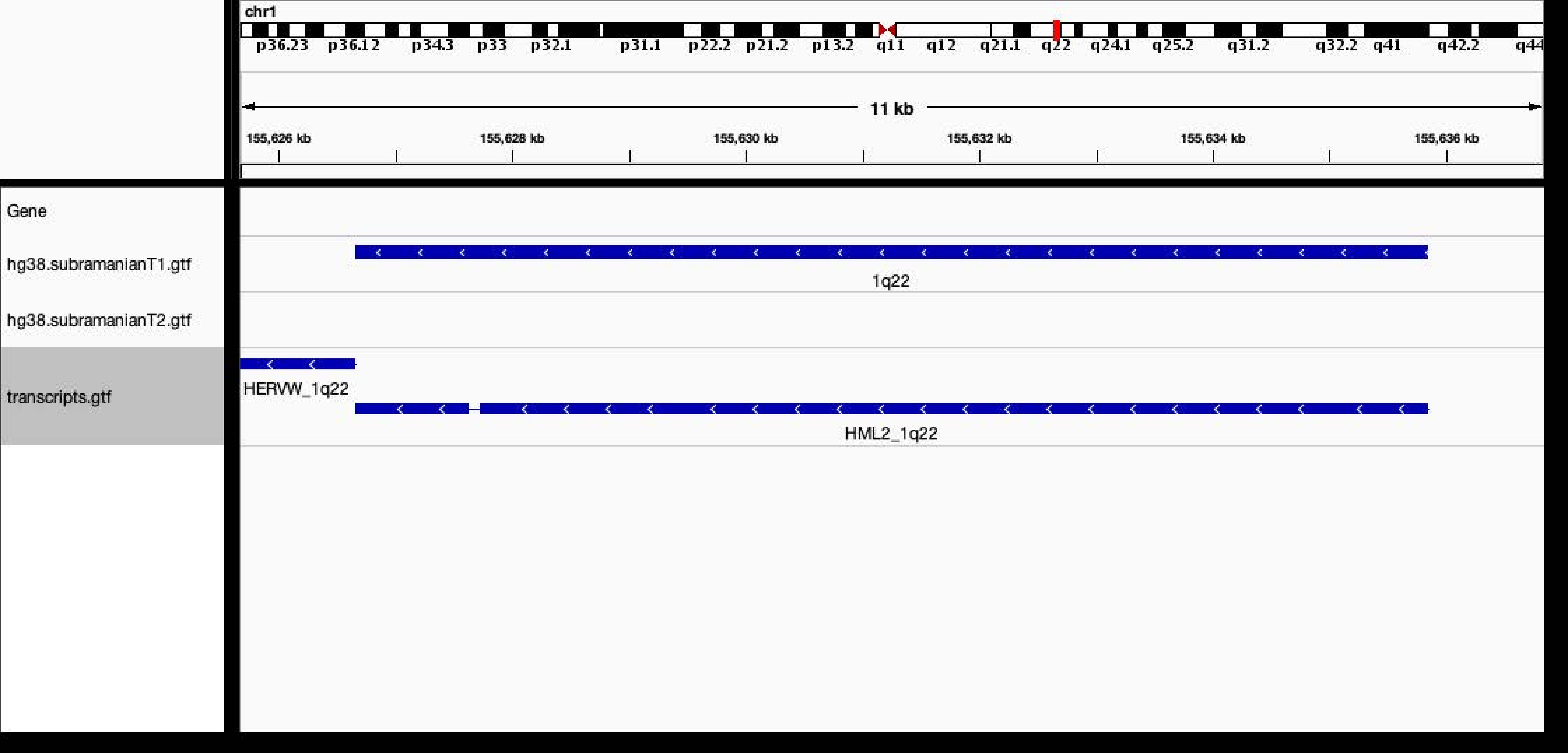
chr1
p36.23
p36.12
p34.3
p33
p32.1
p31.1
p22.2
p21.2
p13.2
q11
q12
q21.1
q22
q24.1
q25.2
q31.2
q32.2
q41
q42.2
11 kb
155,626 kb
155,628 kb
155,630 kb
155,632 kb
155,634 kb
155,636 kb
Gene
hg38.subramanianT1.gtf
1q22
hg38.subramanianT2.gtf
transcripts.gtf
HERVW_1q22
HML2_1q22

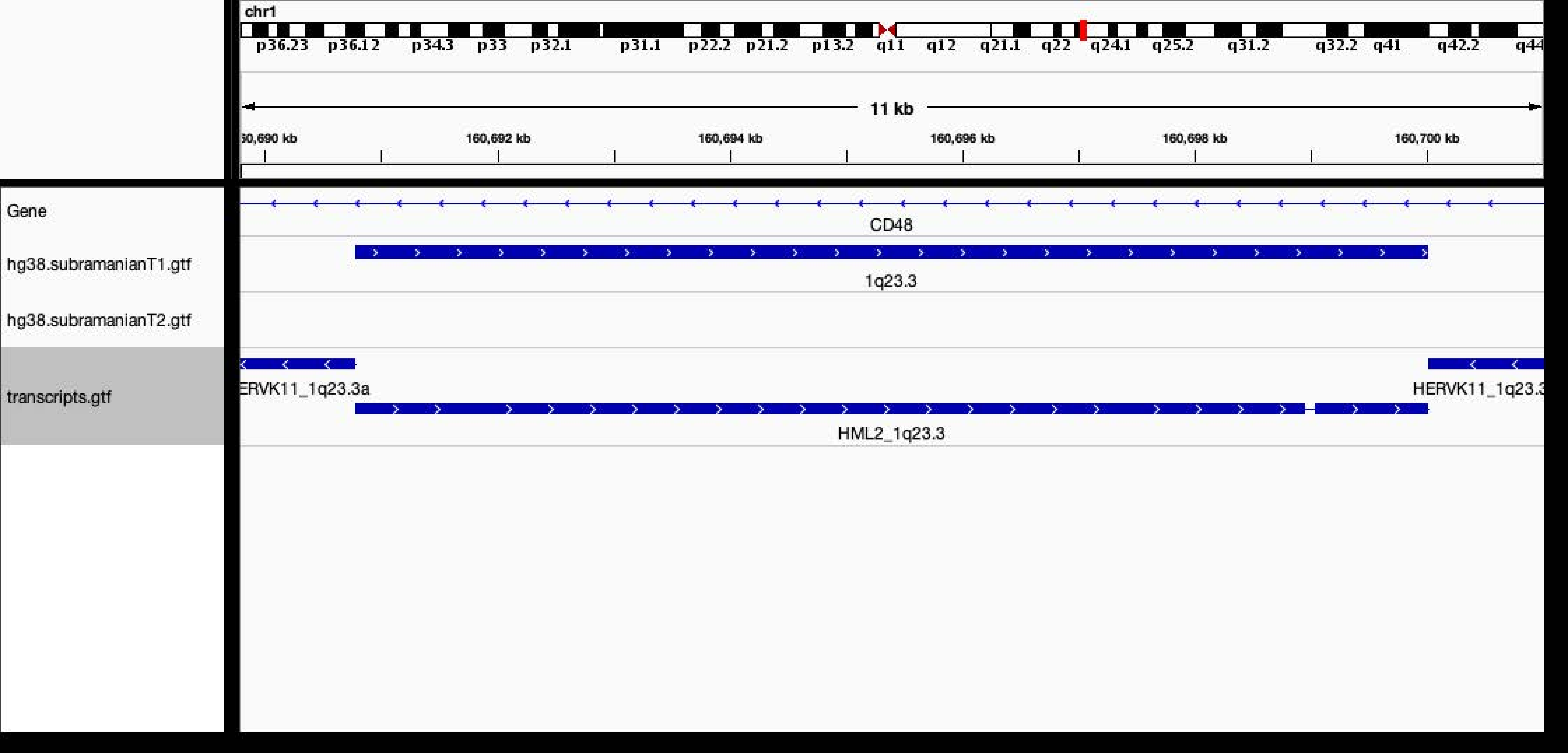
chr1
p36.23
p36.12
p34.3
p33
p32.1
p31.1
p22.2
p21.2
p13.2
q11
q12
q21.1
q22
q24.1
q25.2
q31.2
q32.2
q41
q42.2
11 kb
160,692 kb
160,694 kb
160,696 kb
160,698 kb
160,700 kb
Gene
CD48
hg38.subramanianT1.gtf
1q23.3
hg38.subramanianT2.gtf
transcripts.gtf
HML2_1q23.3

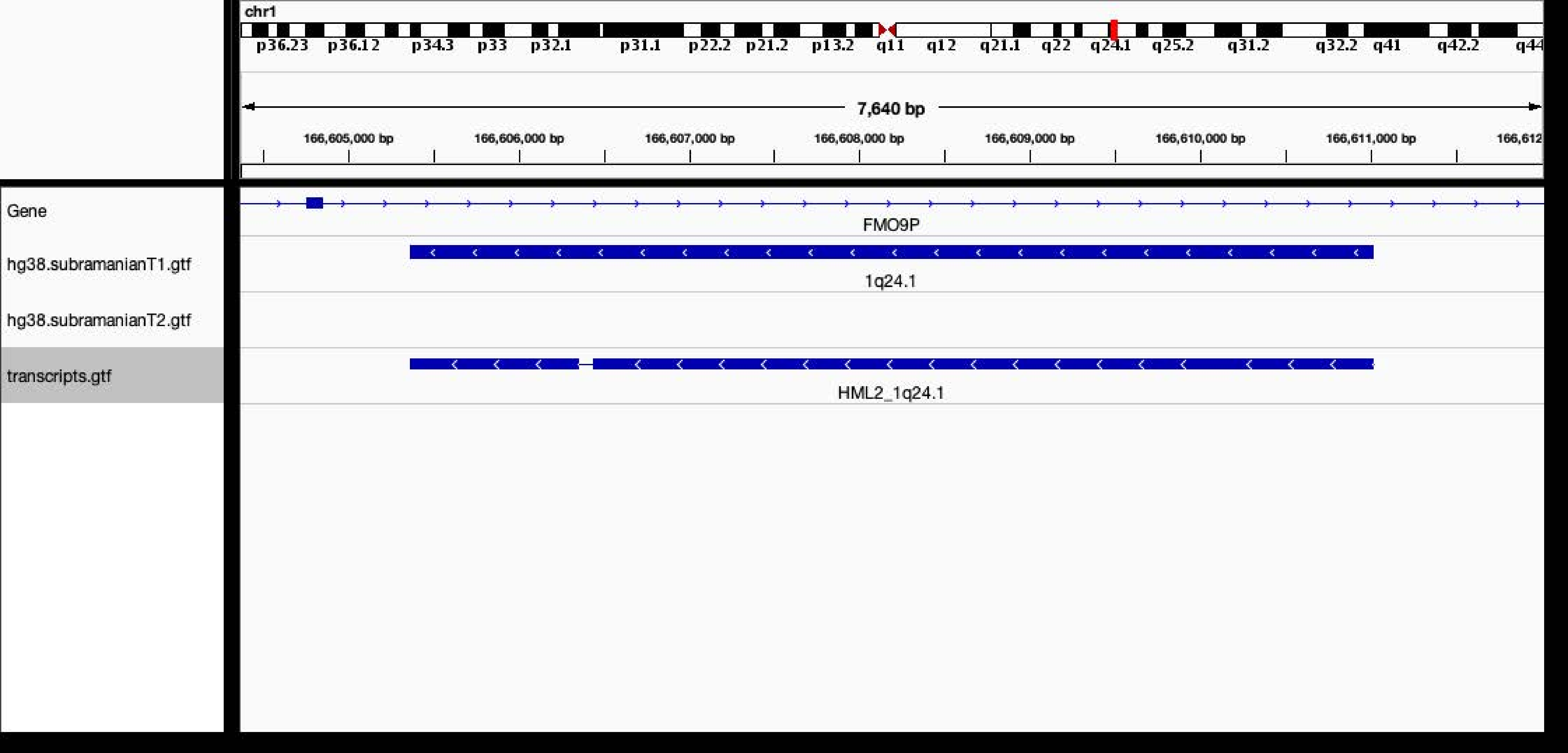
chr1
p36.23 p36.12 p34.3 p33 p32.1 p31.1 p22.2 p21.2 p13.2 q11 q12 q21.1 q22 q24.1 q25.2 q31.2 q32.2 q41 q42.2 q44
7,640 bp
166,605,000 bp 166,606,000 bp 166,607,000 bp 166,608,000 bp 166,609,000 bp 166,610,000 bp 166,611,000 bp
Gene
FMO9P
hg38.subramanianT1.gtf
1q24.1
hg38.subramanianT2.gtf
transcripts.gtf
HML2_1q24.1

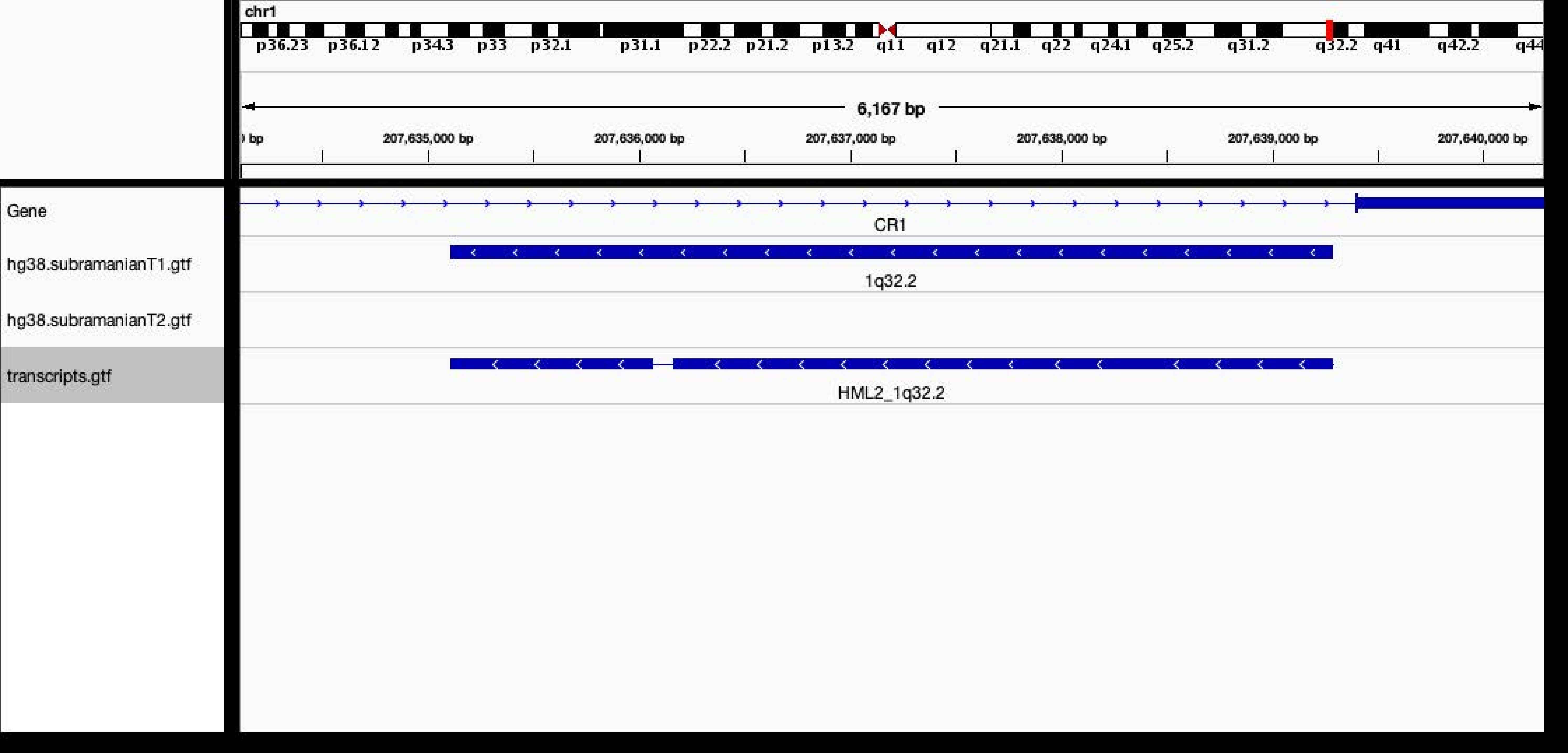

chr1
p36.23 p36.12 p34.3 p33 p32.1 p31.1 p22.2 p21.2 p13.2 q11 q12 q21.1 q22 q24.1 q25.2 q31.2 q32.2 q41 q42.2 q44
6,167 bp
207,635,000 bp
207,636,000 bp
207,637,000 bp
207,638,000 bp
207,639,000 bp
207,640,000 bp
Gene
CR1
hg38.subramanianT1.gtf
1q32.2
hg38.subramanianT2.gtf
transcripts.gtf
HML2_1q32.2

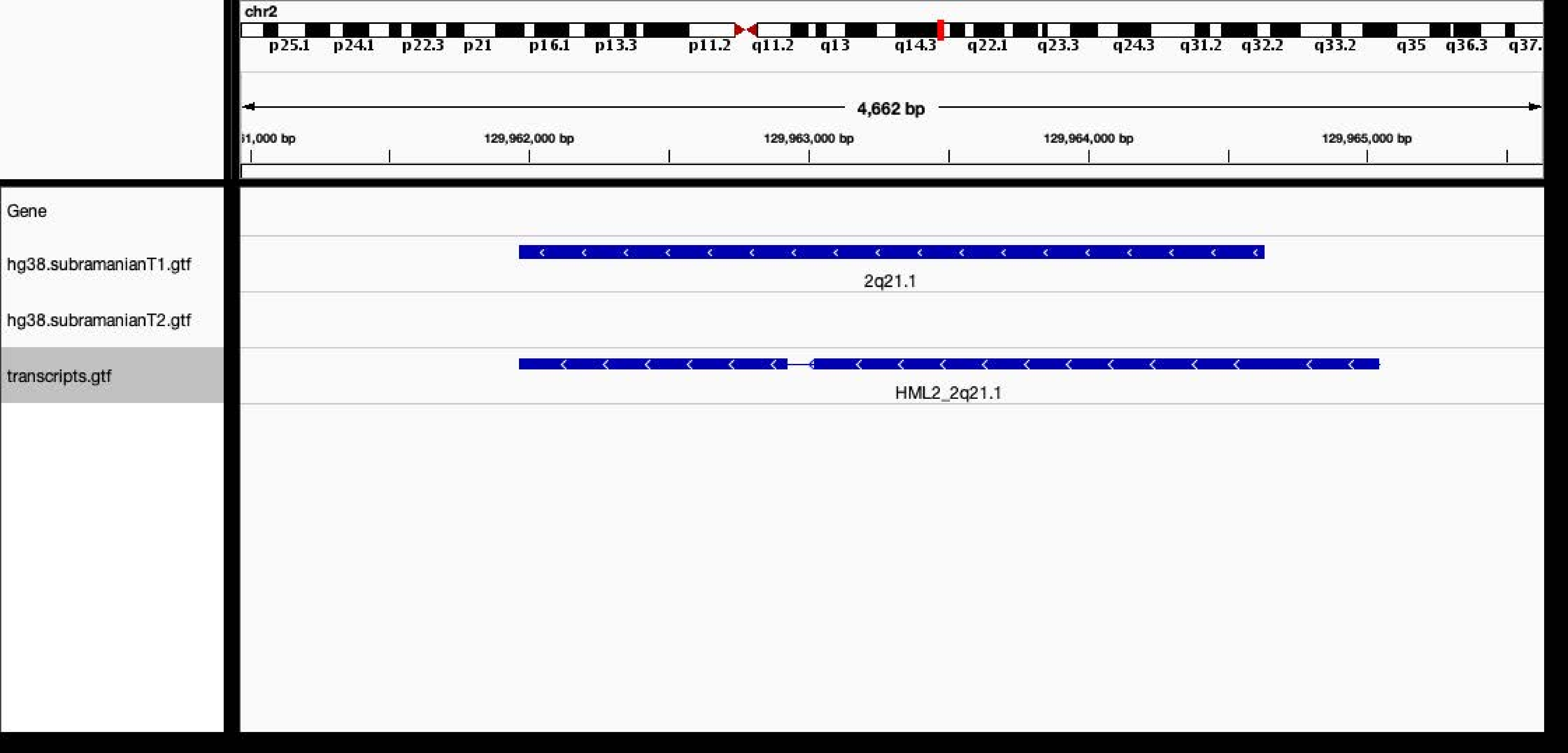
chr2
p25.1 p24.1 p22.3 p21 p16.1 p13.3 p11.2 q11.2 q13 q14.3 q22.1 q23.3 q24.3 q31.2 q32.2 q33.2 q35 q36.3 q37.
4,662 bp
129,962,000 bp
129,963,000 bp
129,964,000 bp
129,965,000 bp
Gene
hg38.subramanianT1.gtf
hg38.subramanianT2.gtf
transcripts.gtf
2q21.1
HML2_2q21.1

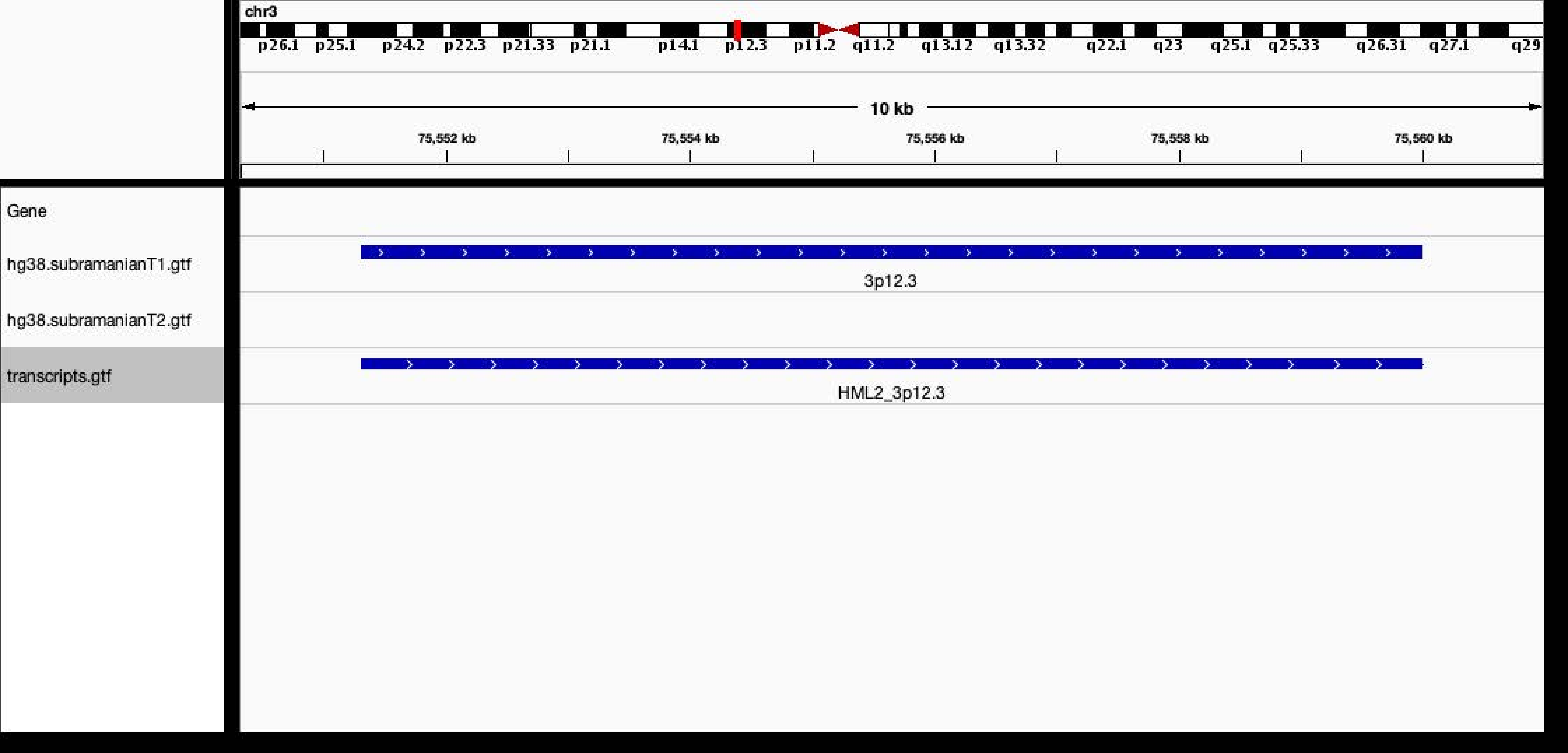
chr3
p26.1
p25.1
p24.2
p22.3
p21.33
p21.1
p14.1
p12.3
p11.2
q11.2
q13.12
q13.32
q22.1
q23
q25.1
q25.33
q26.31
q27.1
q29
10 kb
75,552 kb
75,554 kb
75,556 kb
75,558 kb
75,560 kb
Gene
hg38.subramanianT1.gtf
3p12.3
hg38.subramanianT2.gtf
transcripts.gtf
HML2_3p12.3

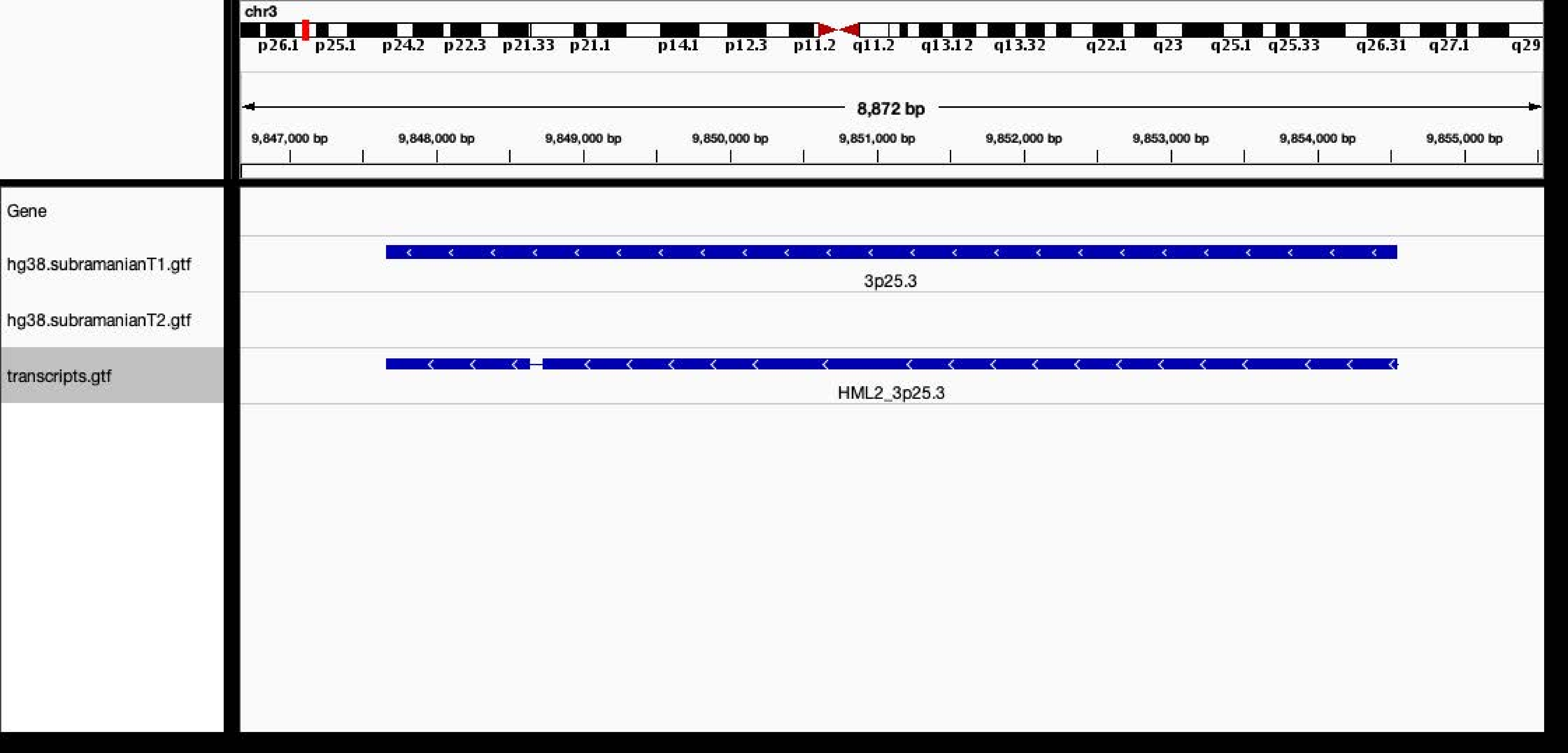

chr3
p26.1
p25.1
p24.2
p22.3
p21.33
p21.1
p14.1
p12.3
p11.2
q11.2
q13.12
q13.32
q22.1
q23
q25.1
q25.33
q26.31
q27.1
q29
8,872 bp
9,847,000 bp
9,848,000 bp
9,849,000 bp
9,850,000 bp
9,851,000 bp
9,852,000 bp
9,853,000 bp
9,854,000 bp
9,855,000 bp
Gene
hg38.subramanianT1.gtf
3p25.3
hg38.subramanianT2.gtf
transcripts.gtf
HML2_3p25.3

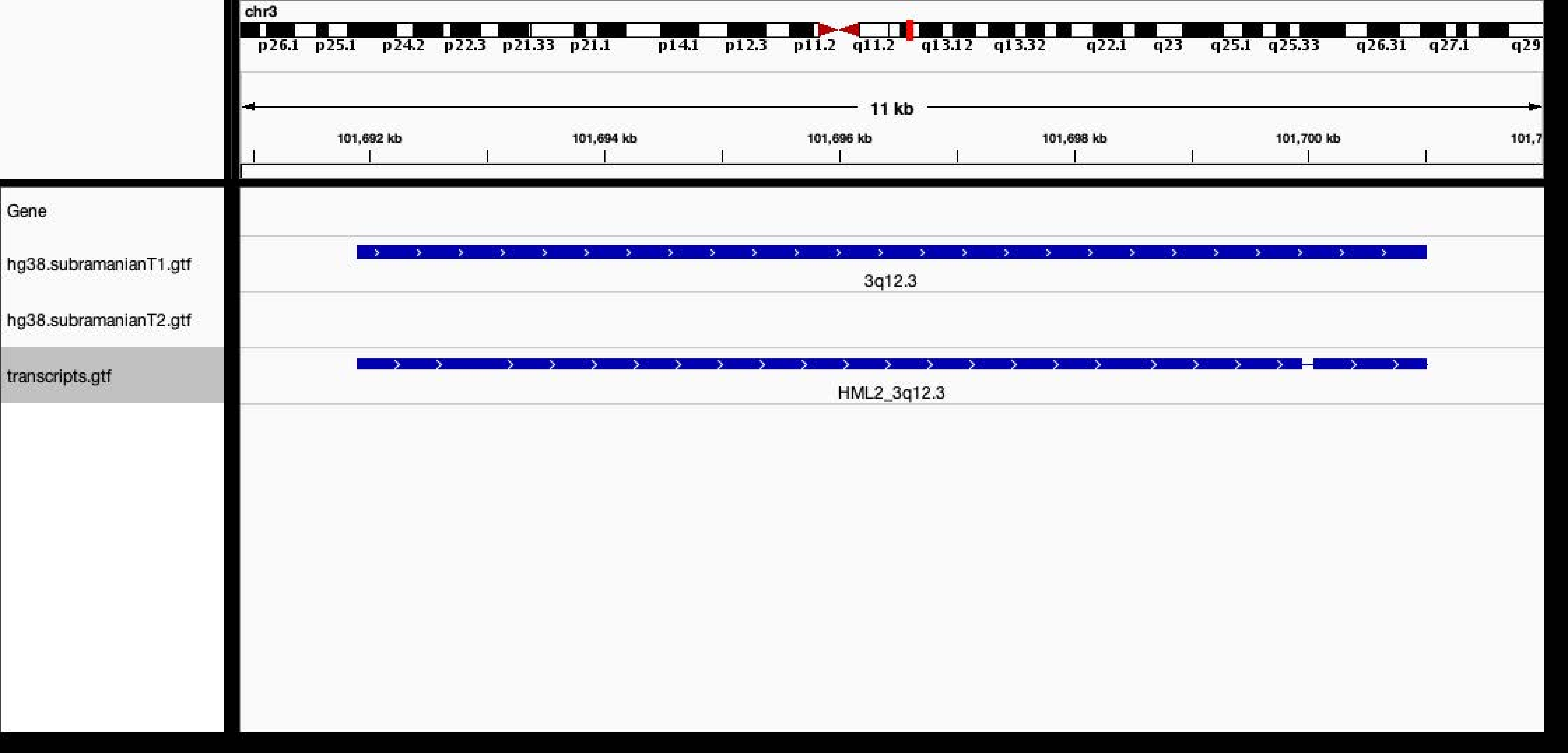

chr3
p26.1 p25.1 p24.2 p22.3 p21.33 p21.1 p14.1 p12.3 p11.2 q11.2 q13.12 q13.32 q22.1 q23 q25.1 q25.33 q26.31 q27.1 q29
11 kb
101,692 kb
101,694 kb
101,696 kb
101,698 kb
101,700 kb
Gene
hg38.subramanianT1.gtf
3q12.3
hg38.subramanianT2.gtf
transcripts.gtf
HML2_3q12.3

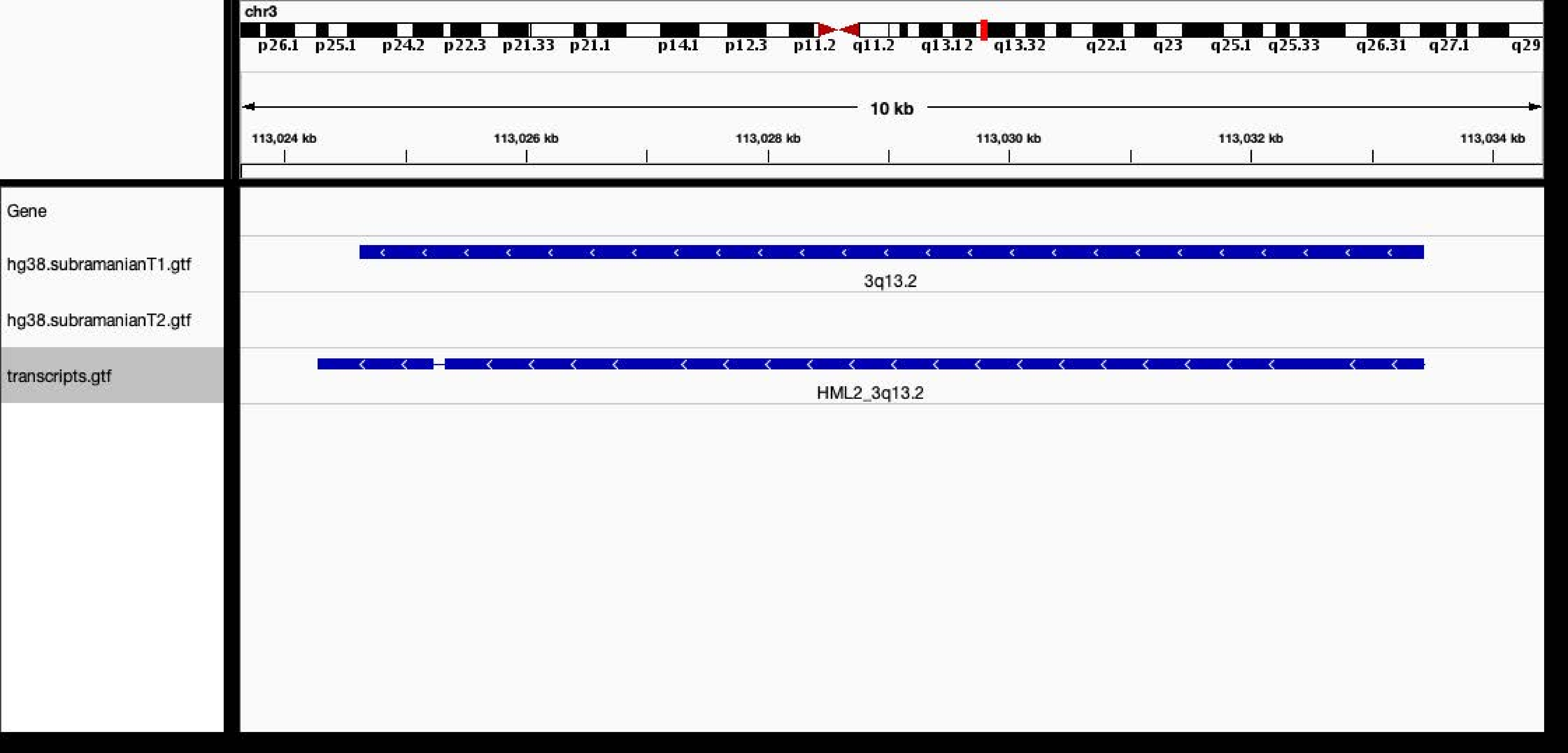

chr3
p26.1
p25.1
p24.2
p22.3
p21.33
p21.1
p14.1
p12.3
p11.2
q11.2
q13.12
q13.32
q22.1
q23
q25.1
q25.33
q26.31
q27.1
q29
10 kb
113,024 kb
113,026 kb
113,028 kb
113,030 kb
113,032 kb
113,034 kb
Gene
hg38.subramanianT1.gtf
3q13.2
hg38.subramanianT2.gtf
transcripts.gtf
HML2_3q13.2

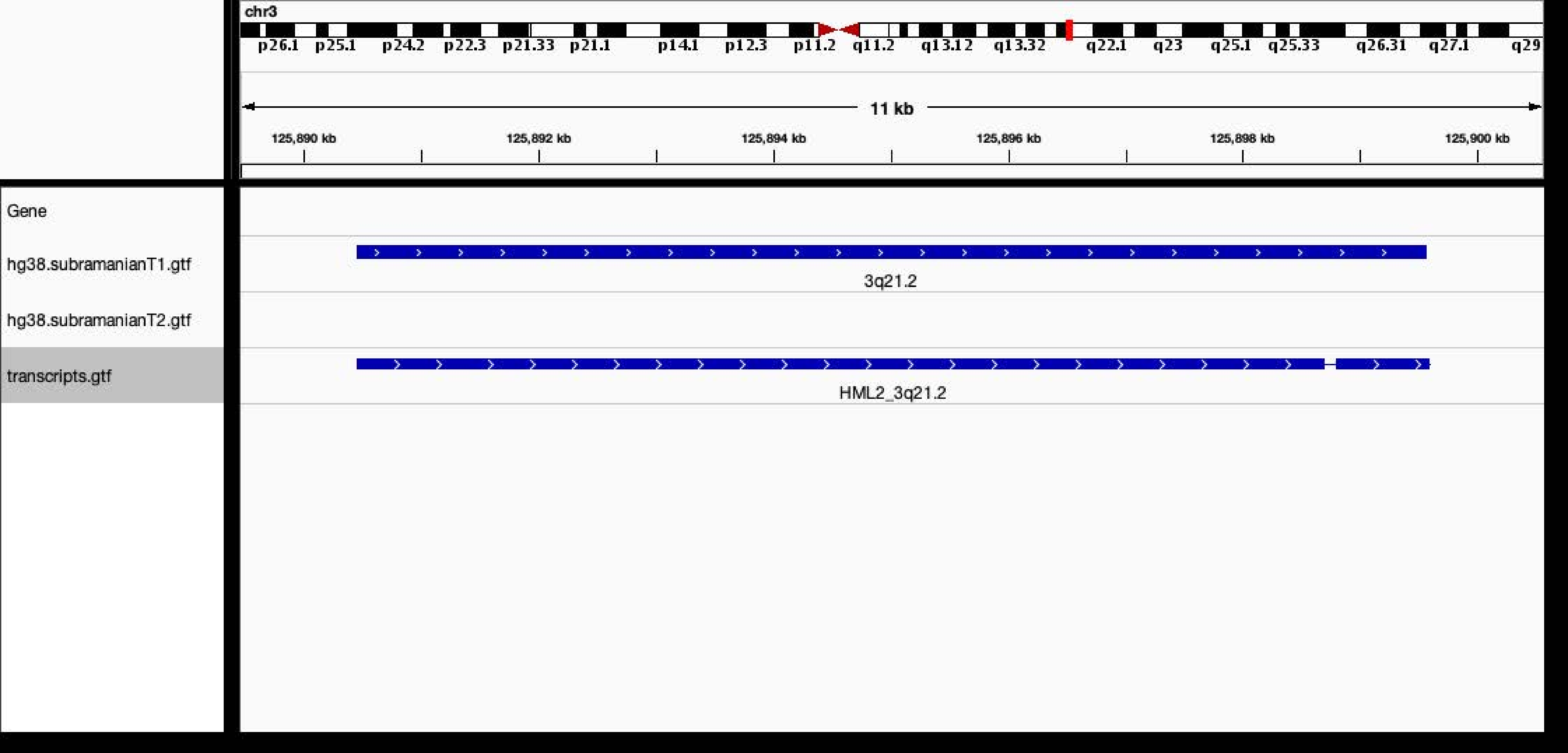
chr3
p26.1 p25.1 p24.2 p22.3 p21.33 p21.1 p14.1 p12.3 p11.2 q11.2 q13.12 q13.32 q22.1 q23 q25.1 q25.33 q26.31 q27.1 q29
11 kb
125,890 kb
125,892 kb
125,894 kb
125,896 kb
125,898 kb
125,900 kb
Gene
hg38.subramanianT1.gtf
3q21.2
hg38.subramanianT2.gtf
transcripts.gtf
HML2_3q21.2

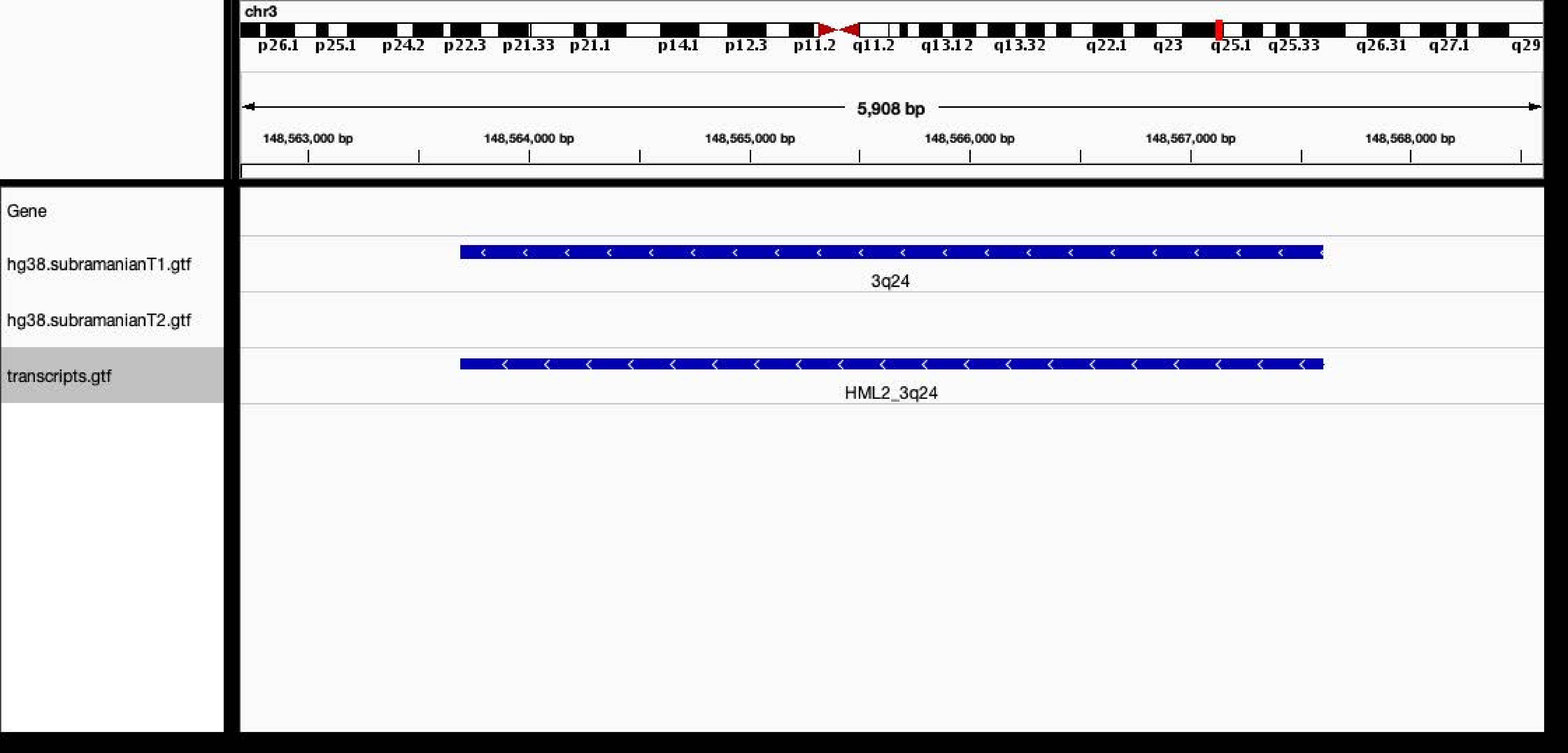
chr3
p26.1
p25.1
p24.2
p22.3
p21.33
p21.1
p14.1
p12.3
p11.2
q11.2
q13.12
q13.32
q22.1
q23
q25.1
q25.33
q26.31
q27.1
q29
5,908 bp
148,563,000 bp
148,564,000 bp
148,565,000 bp
148,566,000 bp
148,567,000 bp
148,568,000 bp
Gene
hg38.subramanianT1.gtf
3q24
hg38.subramanianT2.gtf
transcripts.gtf
HML2_3q24

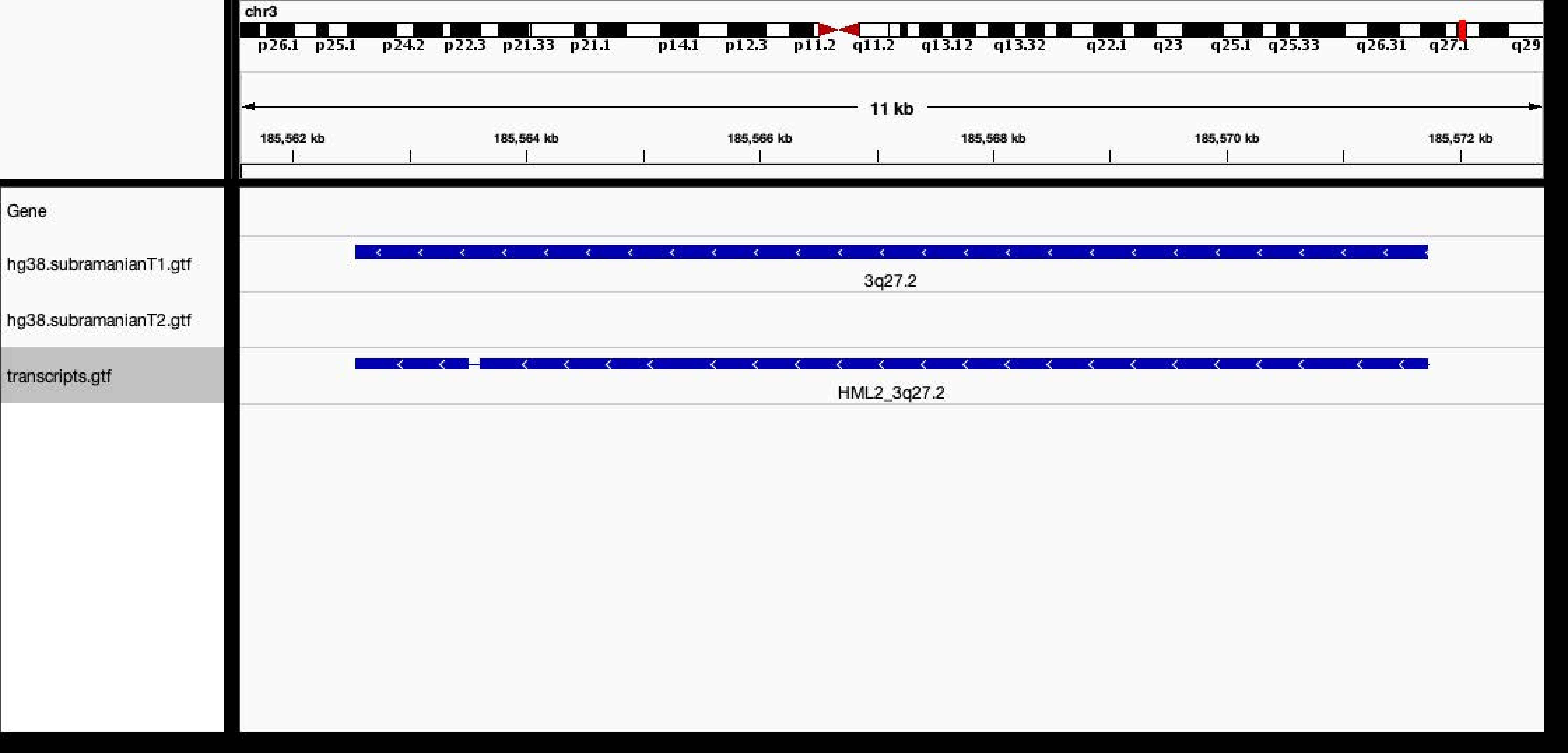
chr3
p26.1 p25.1 p24.2 p22.3 p21.33 p21.1 p14.1 p12.3 p11.2 q11.2 q13.12 q13.32 q22.1 q23 q25.1 q25.33 q26.31 q27.1 q29
11 kb
185,562 kb
185,564 kb
185,566 kb
185,568 kb
185,570 kb
185,572 kb
Gene
hg38.subramanianT1.gtf
3q27.2
hg38.subramanianT2.gtf
transcripts.gtf
HML2_3q27.2

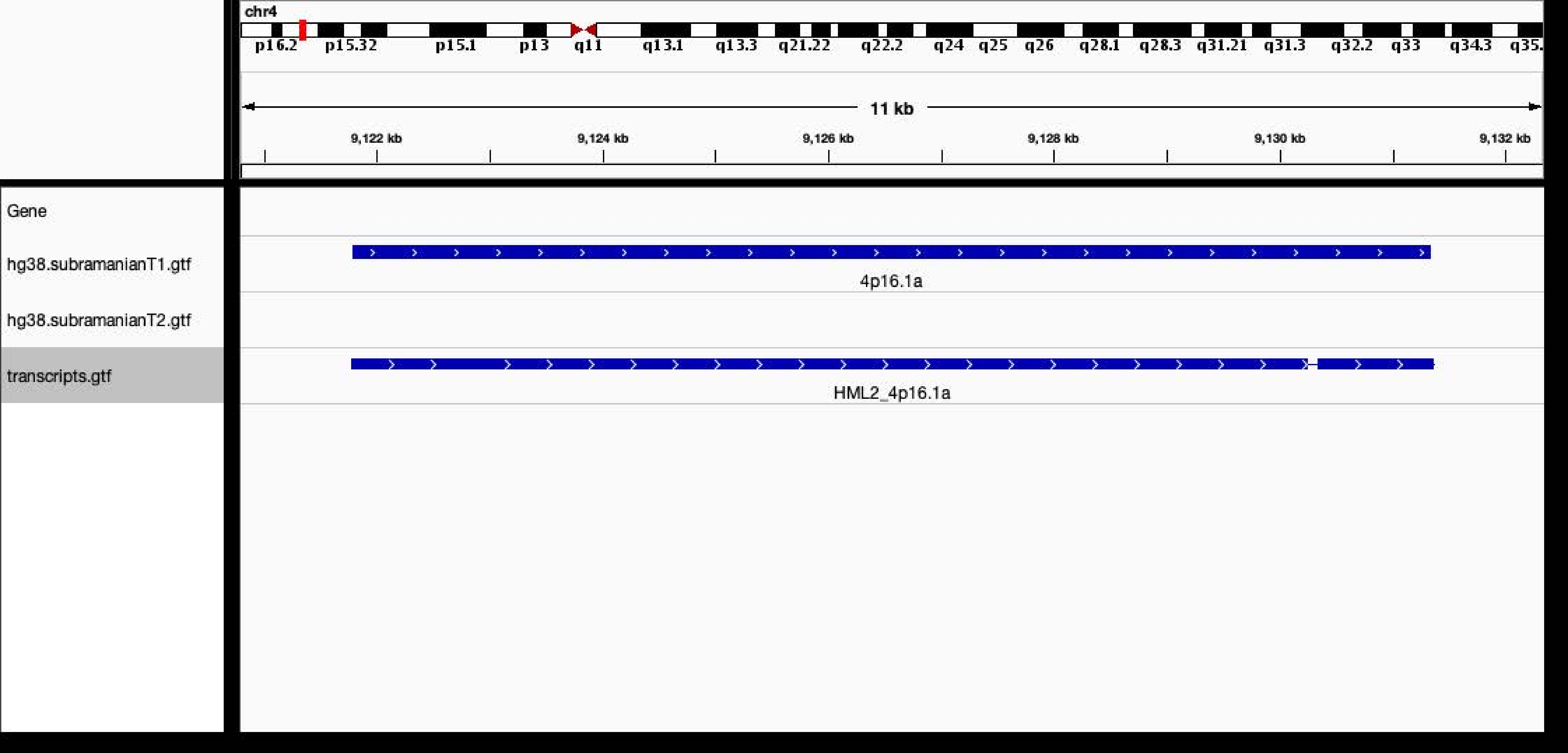

chr4
p16.2
p15.32
p15.1
p13
q11
q13.1
q13.3
q21.22
q22.2
q24
q25
q26
q28.1
q28.3
q31.21
q31.3
q32.2
q33
q34.3
q35.
11 kb
9,122 kb
9,124 kb
9,126 kb
9,128 kb
9,130 kb
9,132 kb
Gene
hg38.subramanianT1.gtf
4p16.1a
hg38.subramanianT2.gtf
transcripts.gtf
HML2_4p16.1a

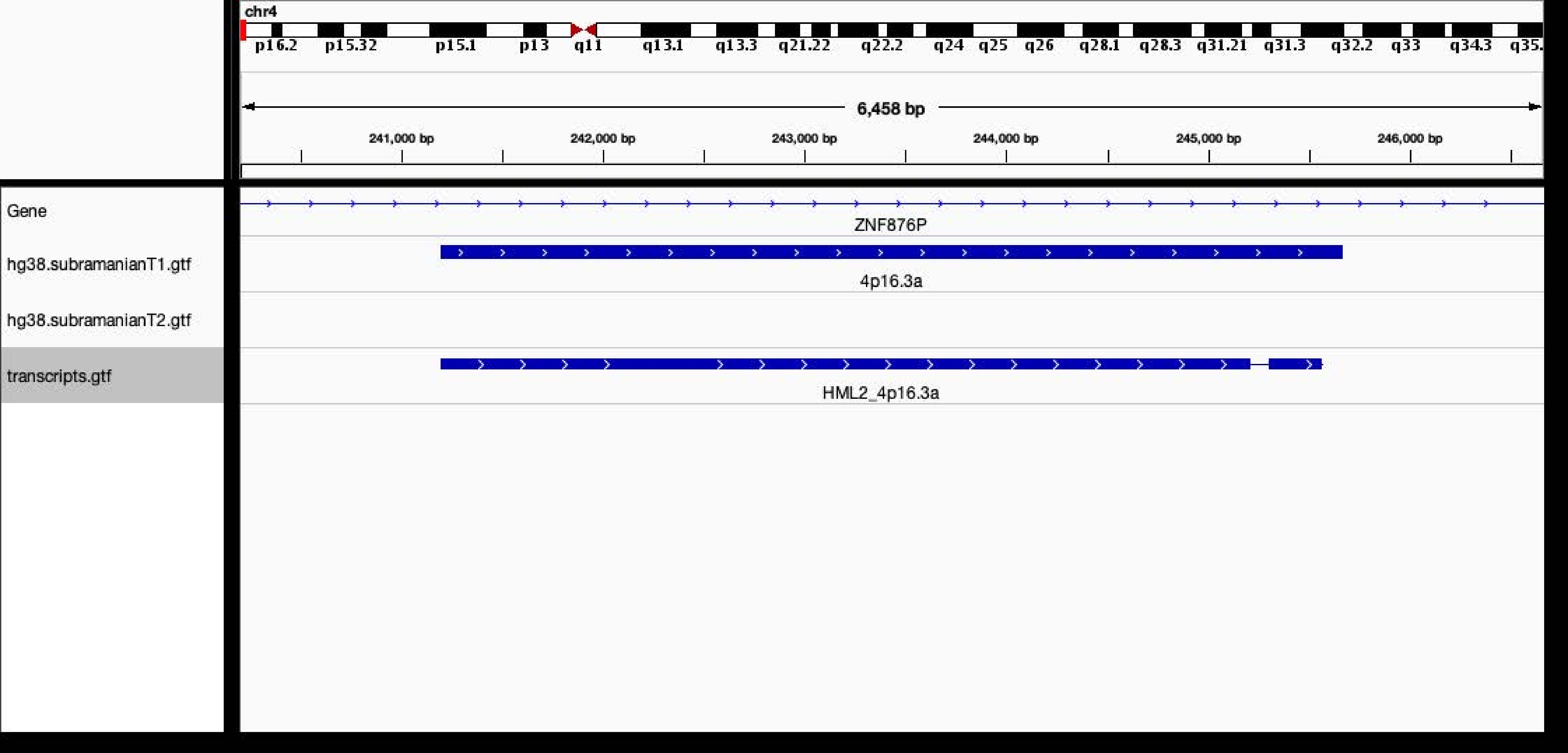
chr4
p16.2
p15.32
p15.1
p13
q11
q13.1
q13.3
q21.22
q22.2
q24
q25
q26
q28.1
q28.3
q31.21
q31.3
q32.2
q33
q34.3
6,458 bp
241,000 bp
242,000 bp
243,000 bp
244,000 bp
245,000 bp
246,000 bp
Gene
ZNF876P
hg38.subramanianT1.gtf
4p16.3a
hg38.subramanianT2.gtf
transcripts.gtf
HML2_4p16.3a

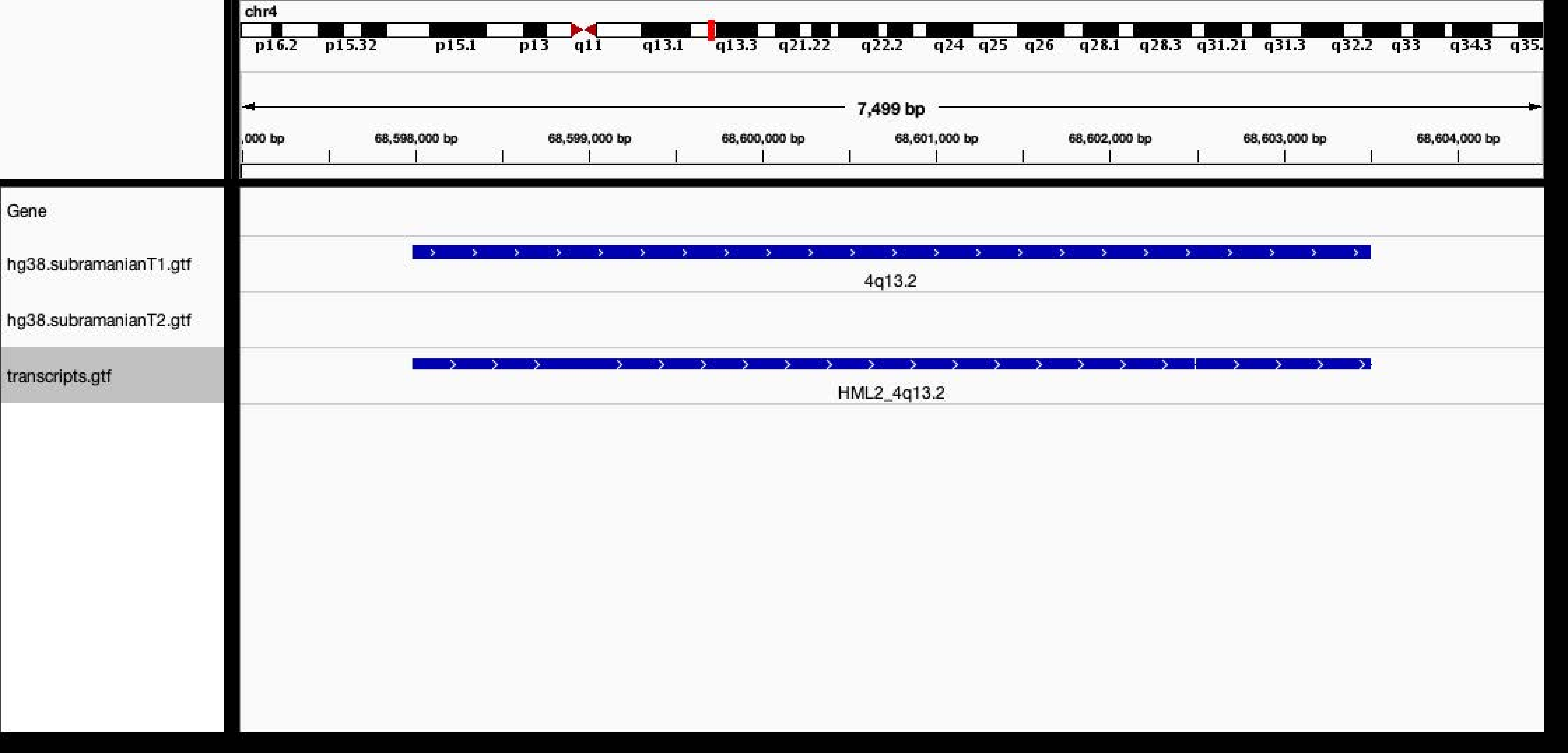
chr4
p16.2
p15.32
p15.1
p13
q11
q13.1
q13.3
q21.22
q22.2
q24
q25
q26
q28.1
q28.3
q31.21
q31.3
q32.2
q33
q34.3
7,499 bp
68,598,000 bp
68,599,000 bp
68,600,000 bp
68,601,000 bp
68,602,000 bp
68,603,000 bp
68,604,000 bp
Gene
hg38.subramanianT1.gtf
4q13.2
hg38.subramanianT2.gtf
transcripts.gtf
HML2_4q13.2

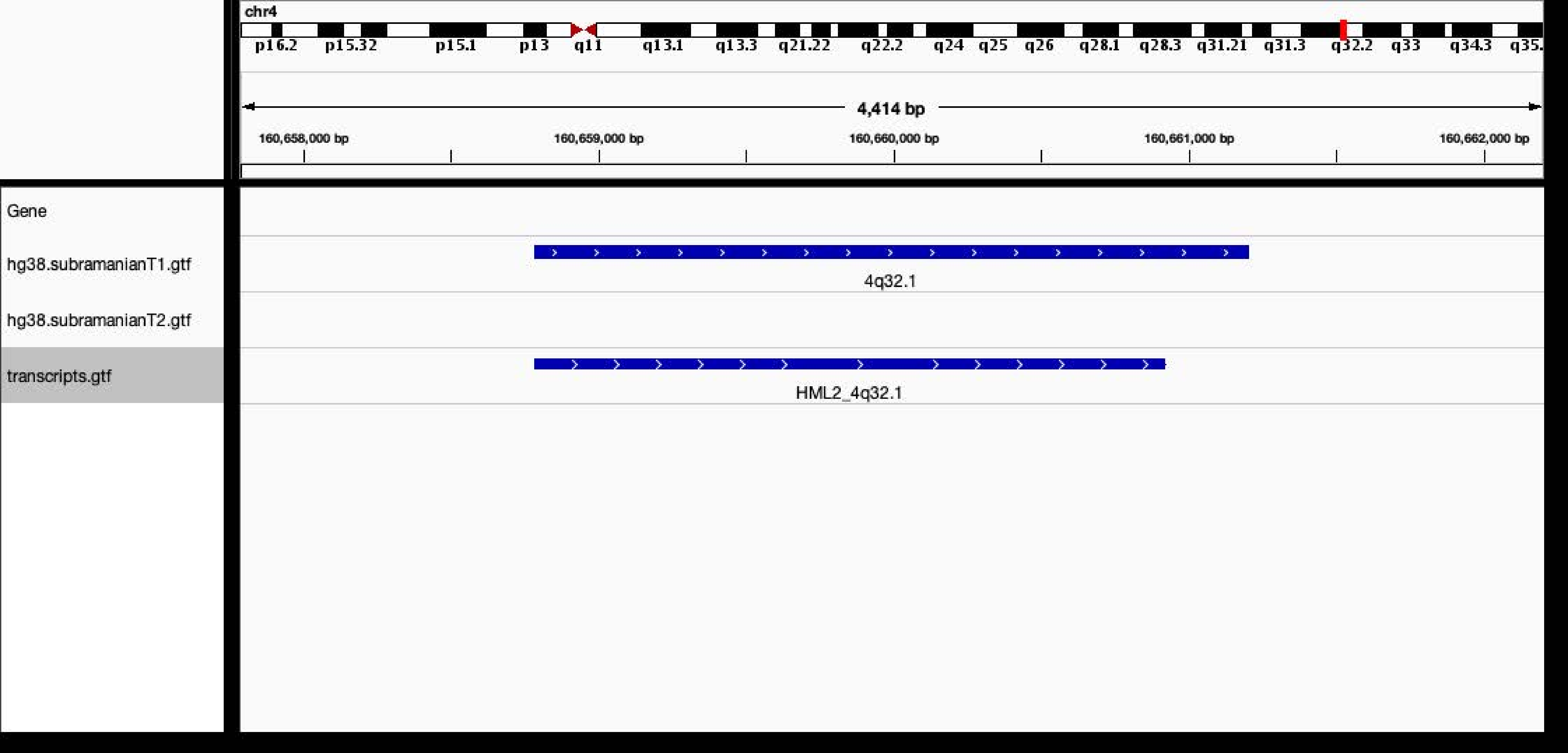
chr4
p16.2
p15.32
p15.1
p13
q11
q13.1
q13.3
q21.22
q22.2
q24
q25
q26
q28.1
q28.3
q31.21
q31.3
q32.2
q33
q34.3
4,414 bp
160,658,000 bp
160,659,000 bp
160,660,000 bp
160,661,000 bp
160,662,000 bp
Gene
hg38.subramanianT1.gtf
4q32.1
hg38.subramanianT2.gtf
transcripts.gtf
HML2_4q32.1

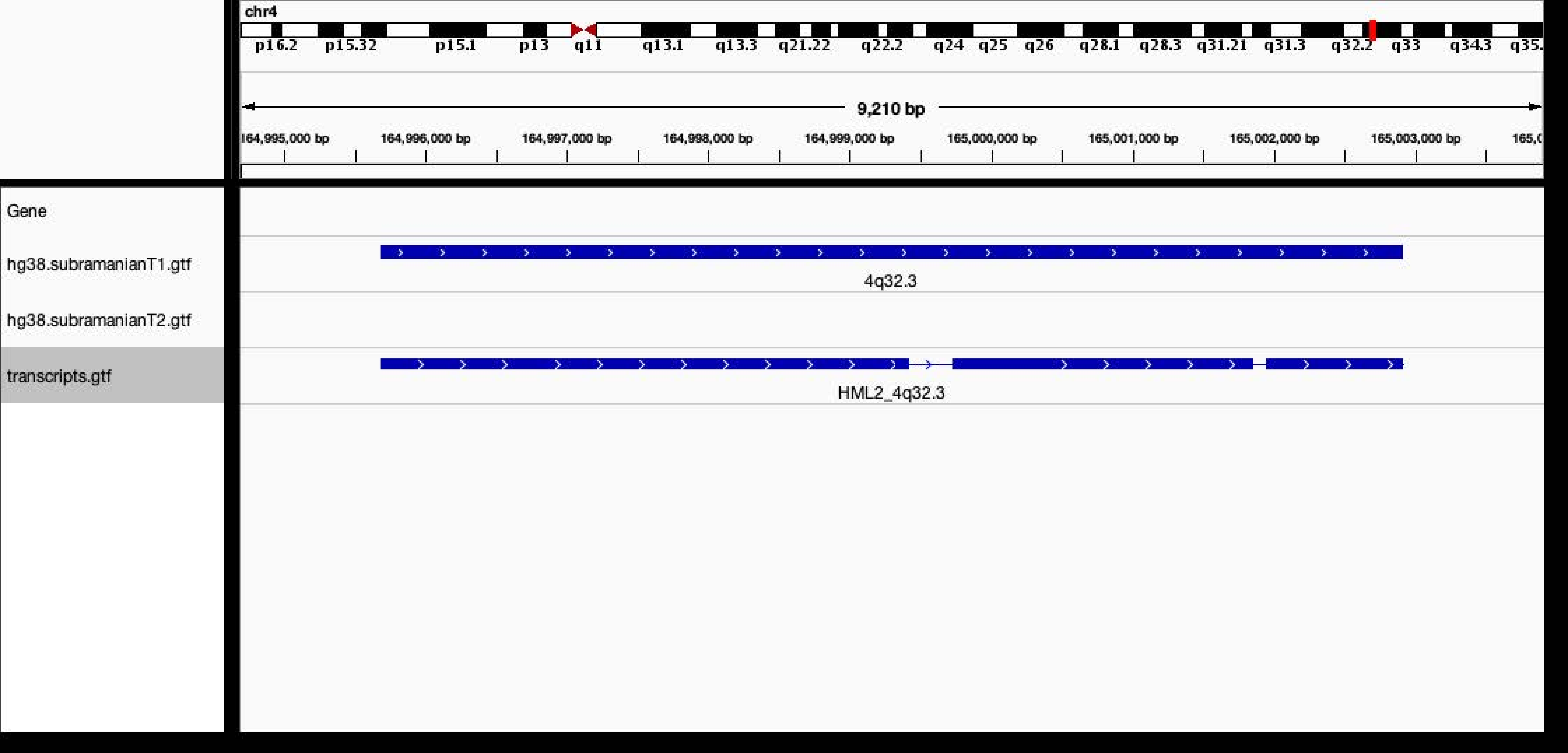
chr4
p16.2
p15.32
p15.1
p13
q11
q13.1
q13.3
q21.22
q22.2
q24
q25
q26
q28.1
q28.3
q31.21
q31.3
q32.2
q33
q34.3
9,210 bp
164,995,000 bp
164,996,000 bp
164,997,000 bp
164,998,000 bp
164,999,000 bp
165,000,000 bp
165,001,000 bp
165,002,000 bp
165,003,000 bp
Gene
hg38.subramanianT1.gtf
4q32.3
hg38.subramanianT2.gtf
transcripts.gtf
HML2_4q32.3

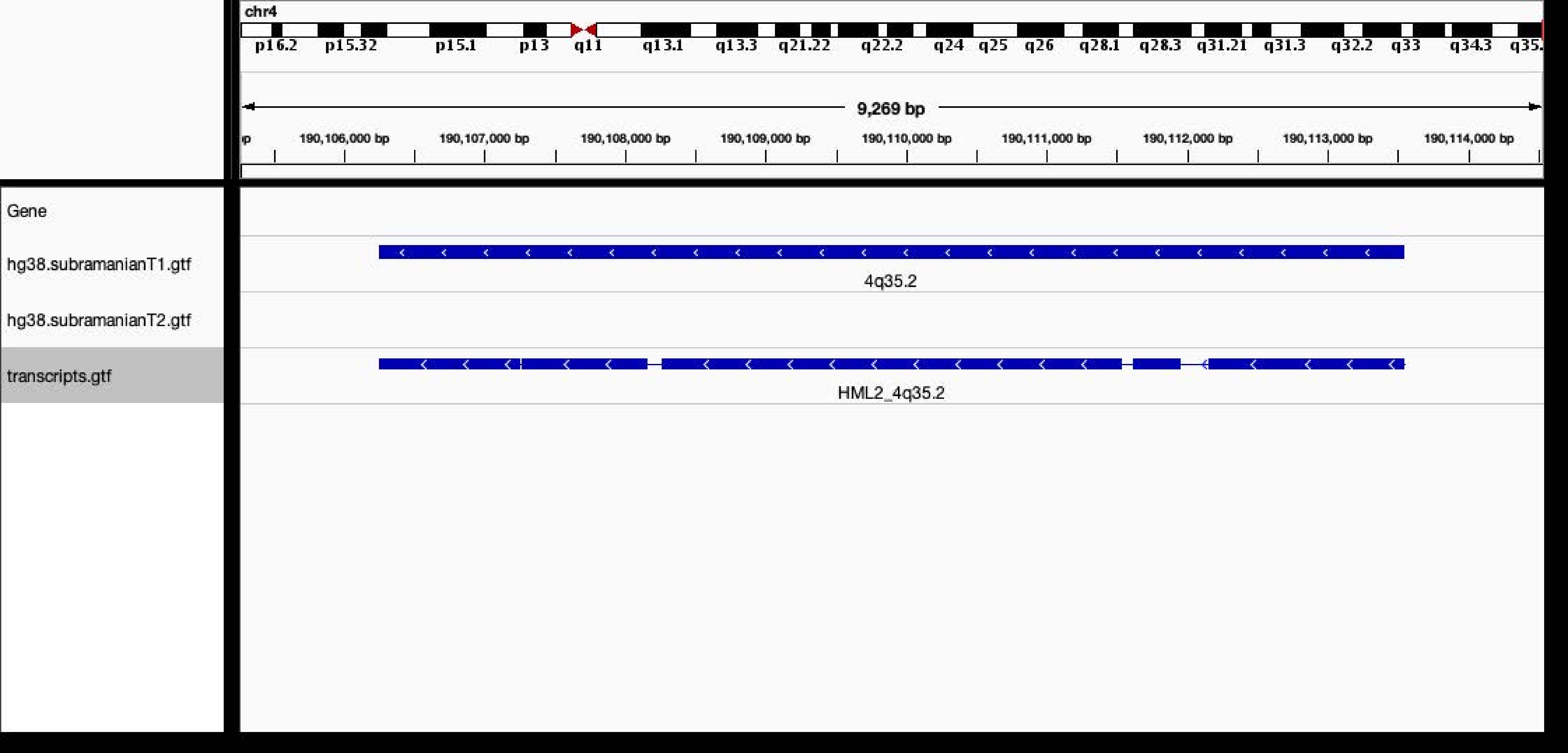
chr4
p16.2
p15.32
p15.1
p13
q11
q13.1
q13.3
q21.22
q22.2
q24
q25
q26
q28.1
q28.3
q31.21
q31.3
q32.2
q33
q34.3
9,269 bp
190,106,000 bp
190,107,000 bp
190,108,000 bp
190,109,000 bp
190,110,000 bp
190,111,000 bp
190,112,000 bp
190,113,000 bp
190,114,000 bp
Gene
hg38.subramanianT1.gtf
4q35.2
hg38.subramanianT2.gtf
transcripts.gtf
HML2_4q35.2

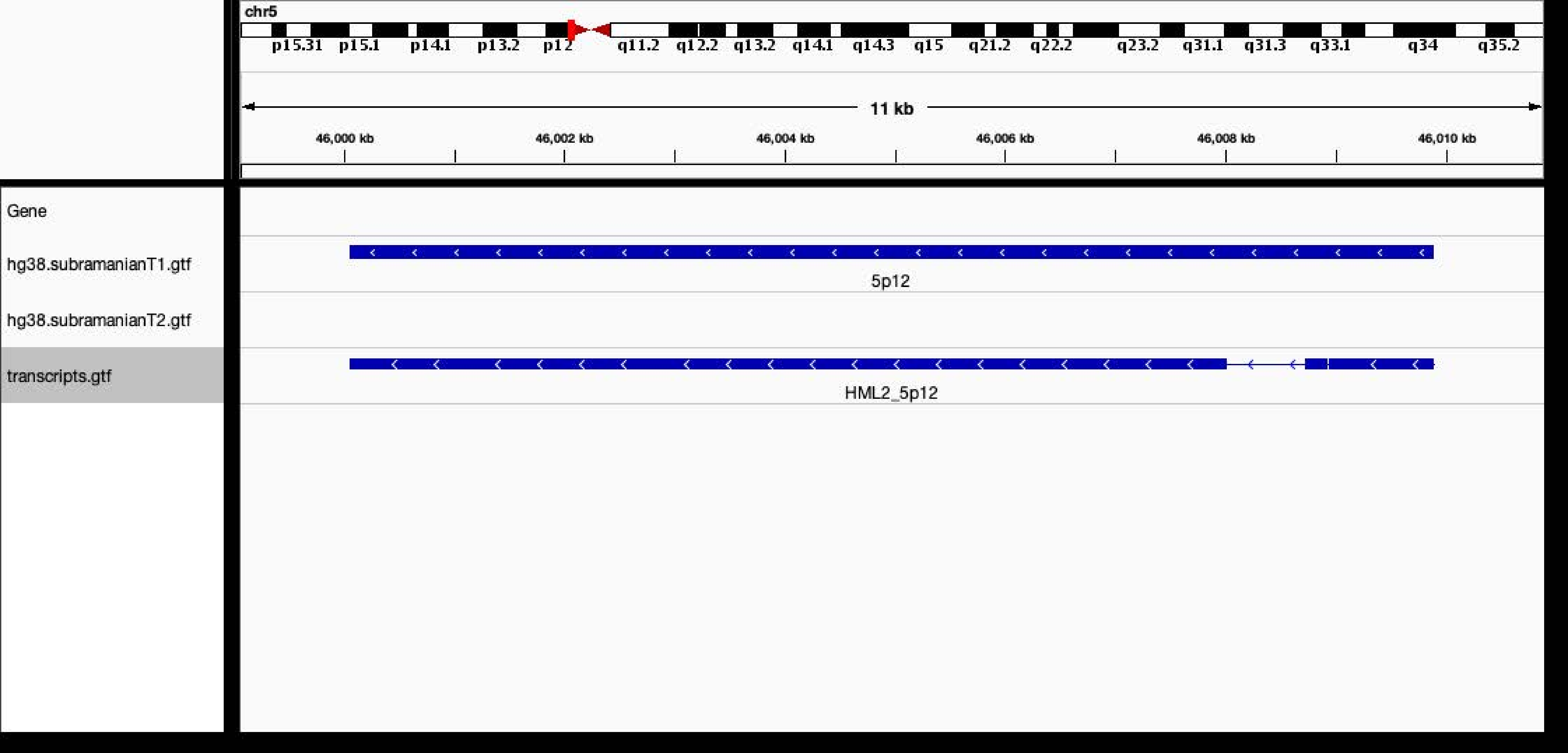
chr5
p15.31 p15.1 p14.1 p13.2 p12 q11.2 q12.2 q13.2 q14.1 q14.3 q15 q21.2 q22.2 q23.2 q31.1 q31.3 q33.1 q34 q35.2
11 kb
46,000 kb
46,002 kb
46,004 kb
46,006 kb
46,008 kb
46,010 kb
Gene
hg38.subramanianT1.gtf
5p12
hg38.subramanianT2.gtf
transcripts.gtf
HML2_5p12

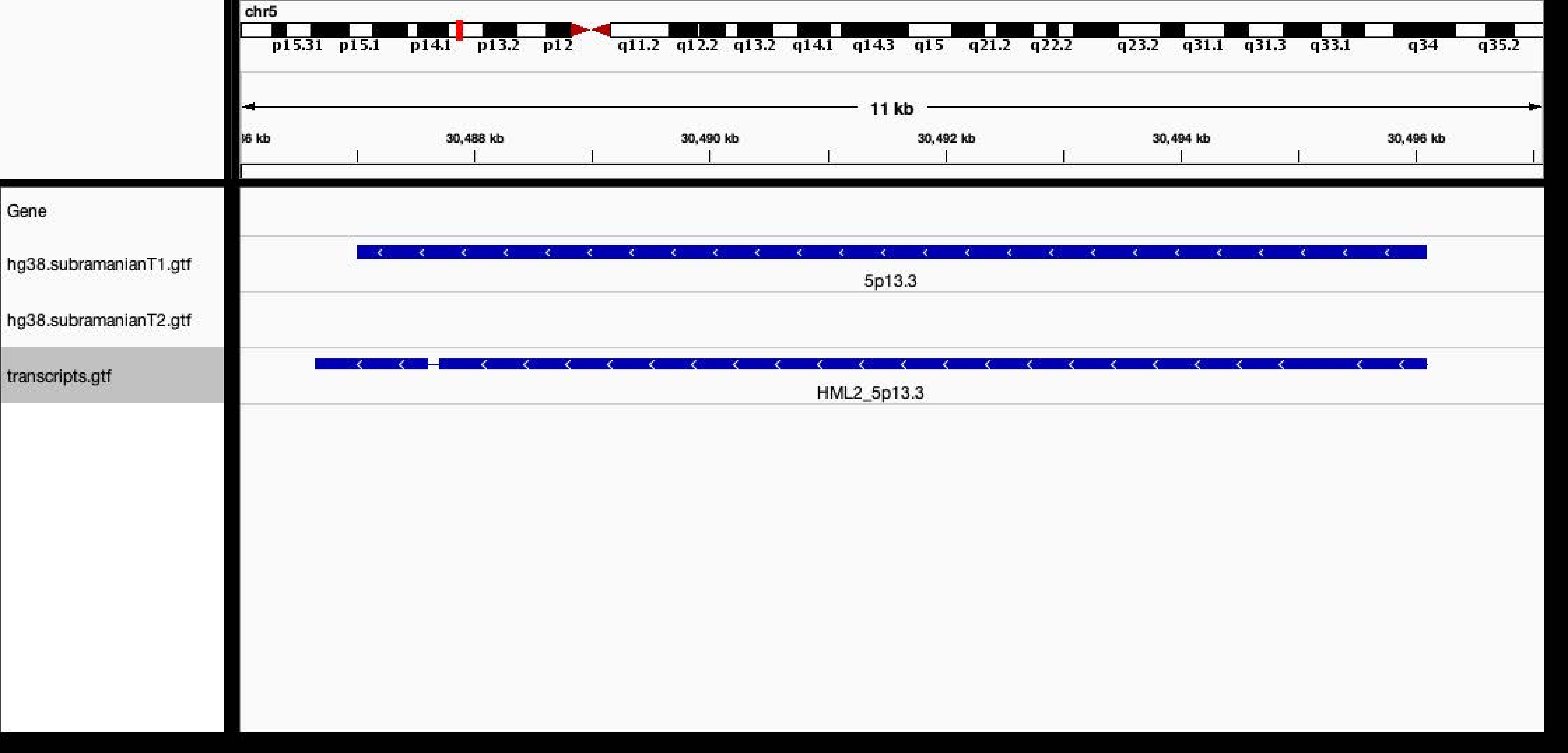

chr5
p15.31 p15.1 p14.1 p13.2 p12 q11.2 q12.2 q13.2 q14.1 q14.3 q15 q21.2 q22.2 q23.2 q31.1 q31.3 q33.1 q34 q35.2
11 kb
30,488 kb
30,490 kb
30,492 kb
30,494 kb
30,496 kb
Gene
hg38.subramanianT1.gtf
5p13.3
hg38.subramanianT2.gtf
transcripts.gtf
HML2_5p13.3

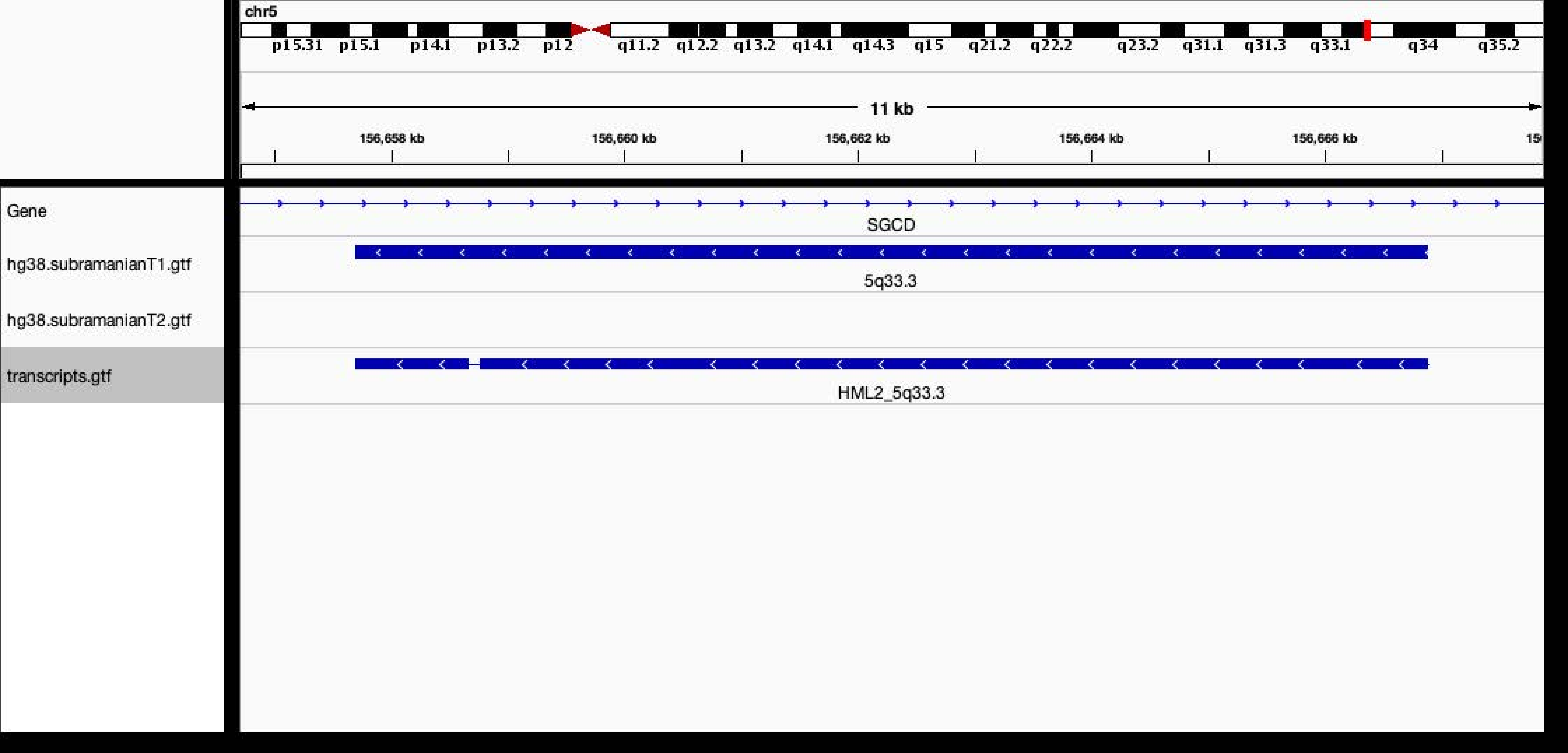

chr5
p15.31 p15.1 p14.1 p13.2 p12 q11.2 q12.2 q13.2 q14.1 q14.3 q15 q21.2 q22.2 q23.2 q31.1 q31.3 q33.1 q34 q35.2
11 kb
156,658 kb
156,660 kb
156,662 kb
156,664 kb
156,666 kb
Gene
SGCD
hg38.subramanianT1.gtf
5q33.3
hg38.subramanianT2.gtf
transcripts.gtf
HML2_5q33.3

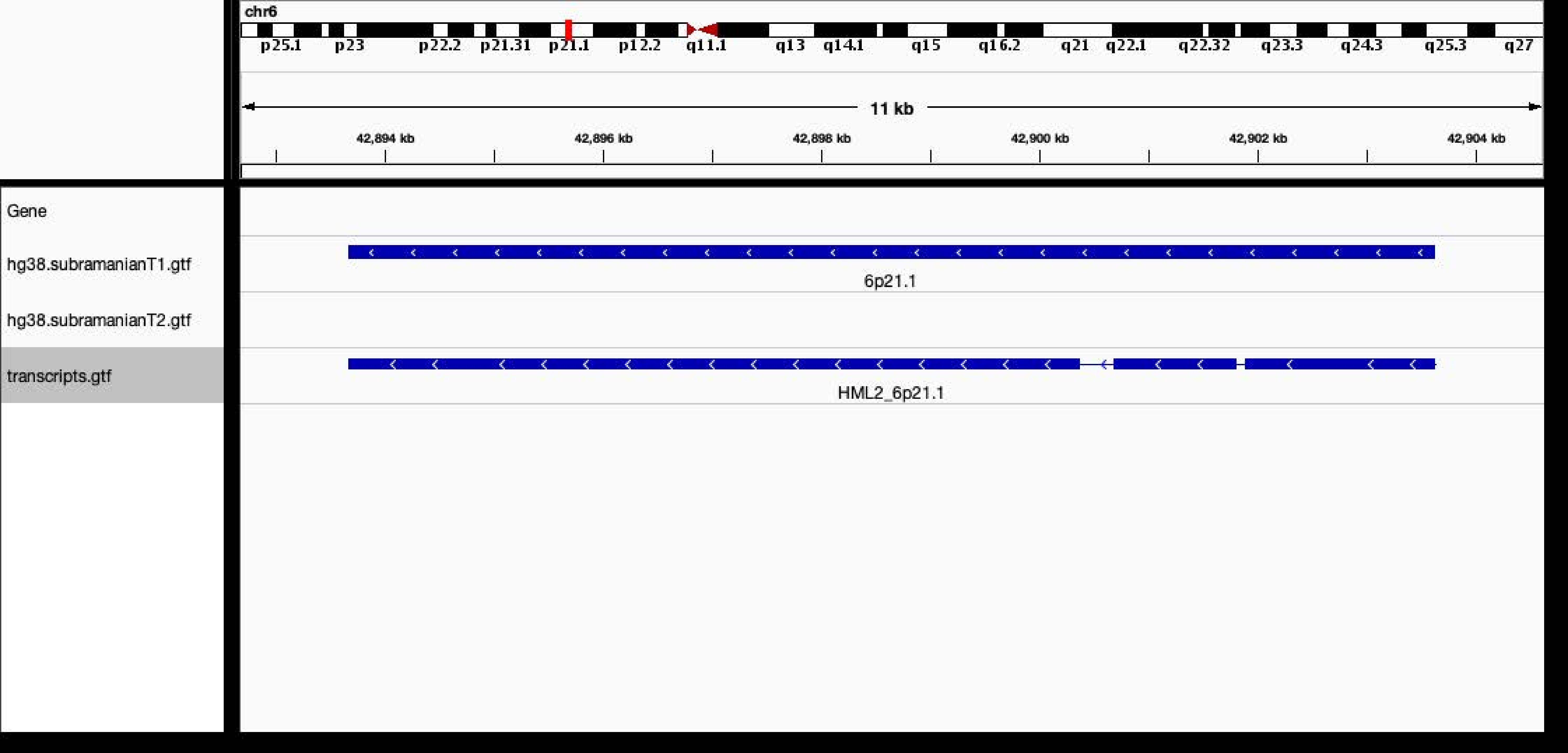

chr6
p25.1
p23
p22.2
p21.31
p21.1
p12.2
q11.1
q13
q14.1
q15
q16.2
q21
q22.1
q22.32
q23.3
q24.3
q25.3
q27
11 kb
42,894 kb
42,896 kb
42,898 kb
42,900 kb
42,902 kb
42,904 kb
Gene
hg38.subramanianT1.gtf
6p21.1
hg38.subramanianT2.gtf
transcripts.gtf
HML2_6p21.1

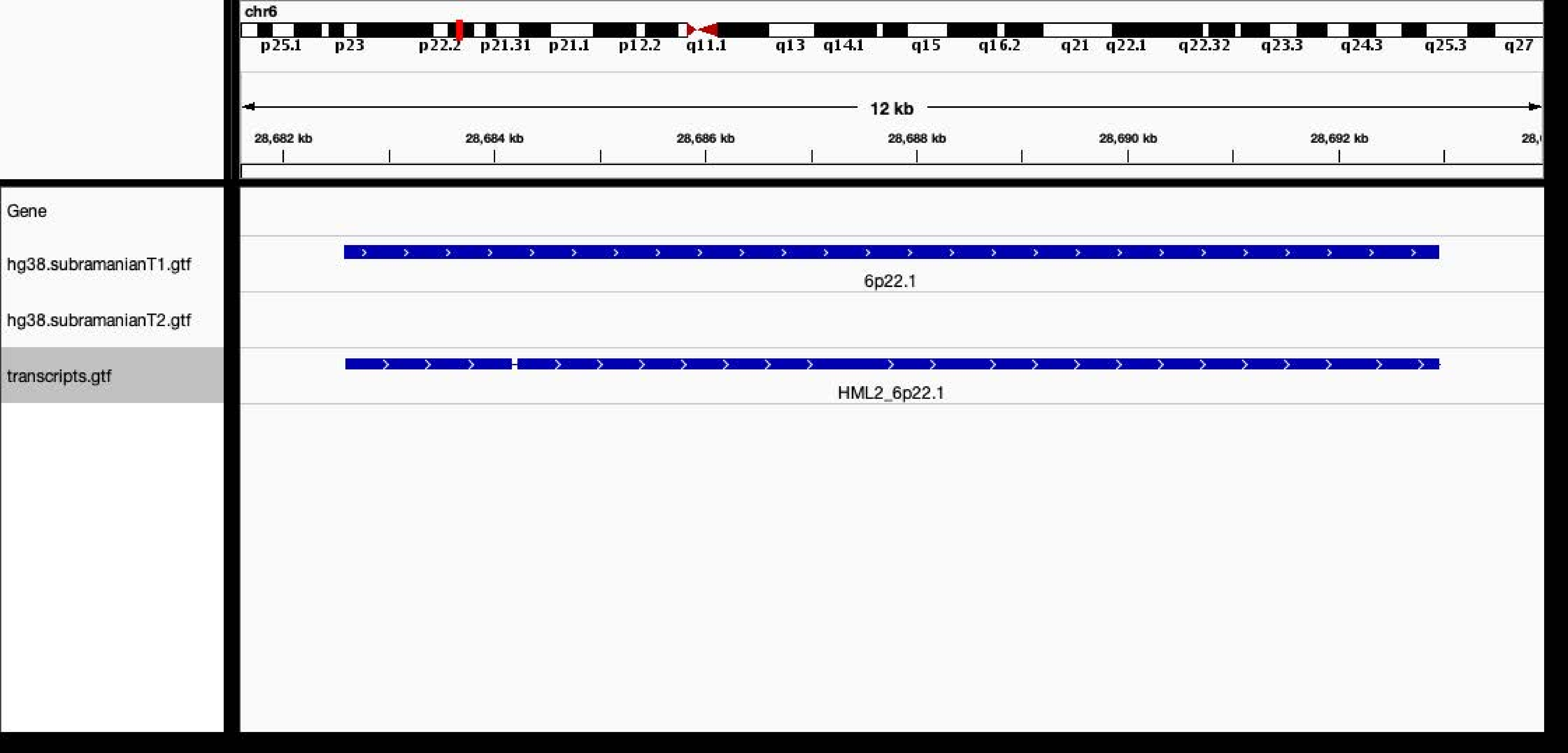
chr6
p25.1
p23
p22.2
p21.31
p21.1
p12.2
q11.1
q13
q14.1
q15
q16.2
q21
q22.1
q22.32
q23.3
q24.3
q25.3
q27
12 kb
28,682 kb
28,684 kb
28,686 kb
28,688 kb
28,690 kb
28,692 kb
Gene
hg38.subramanianT1.gtf
6p22.1
hg38.subramanianT2.gtf
transcripts.gtf
HML2_6p22.1

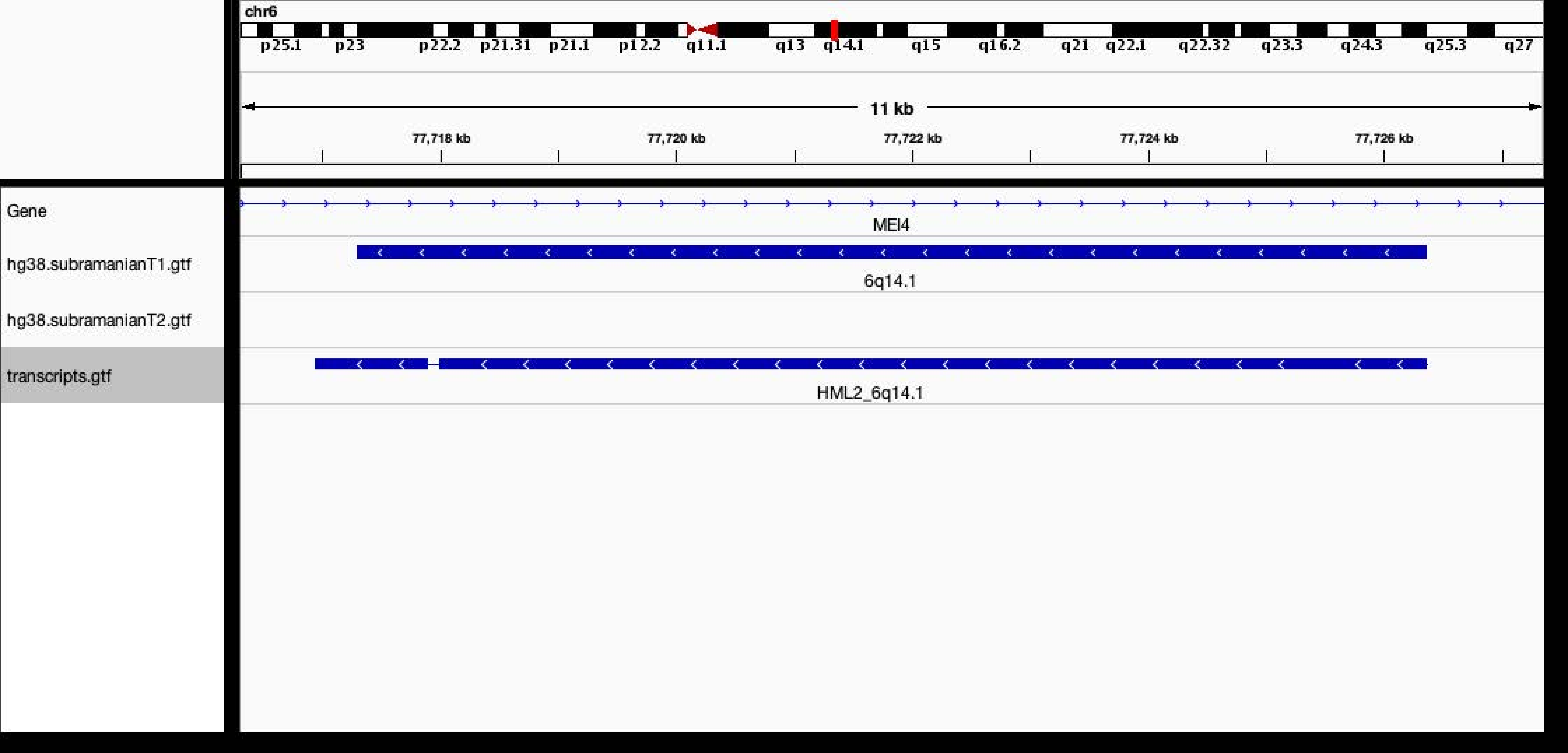
chr6
p25.1
p23
p22.2
p21.31
p21.1
p12.2
q11.1
q13
q14.1
q15
q16.2
q21
q22.1
q22.32
q23.3
q24.3
q25.3
q27
11 kb
77,718 kb
77,720 kb
77,722 kb
77,724 kb
77,726 kb
Gene
MEI4
hg38.subramanianT1.gtf
6q14.1
hg38.subramanianT2.gtf
transcripts.gtf
HML2_6q14.1

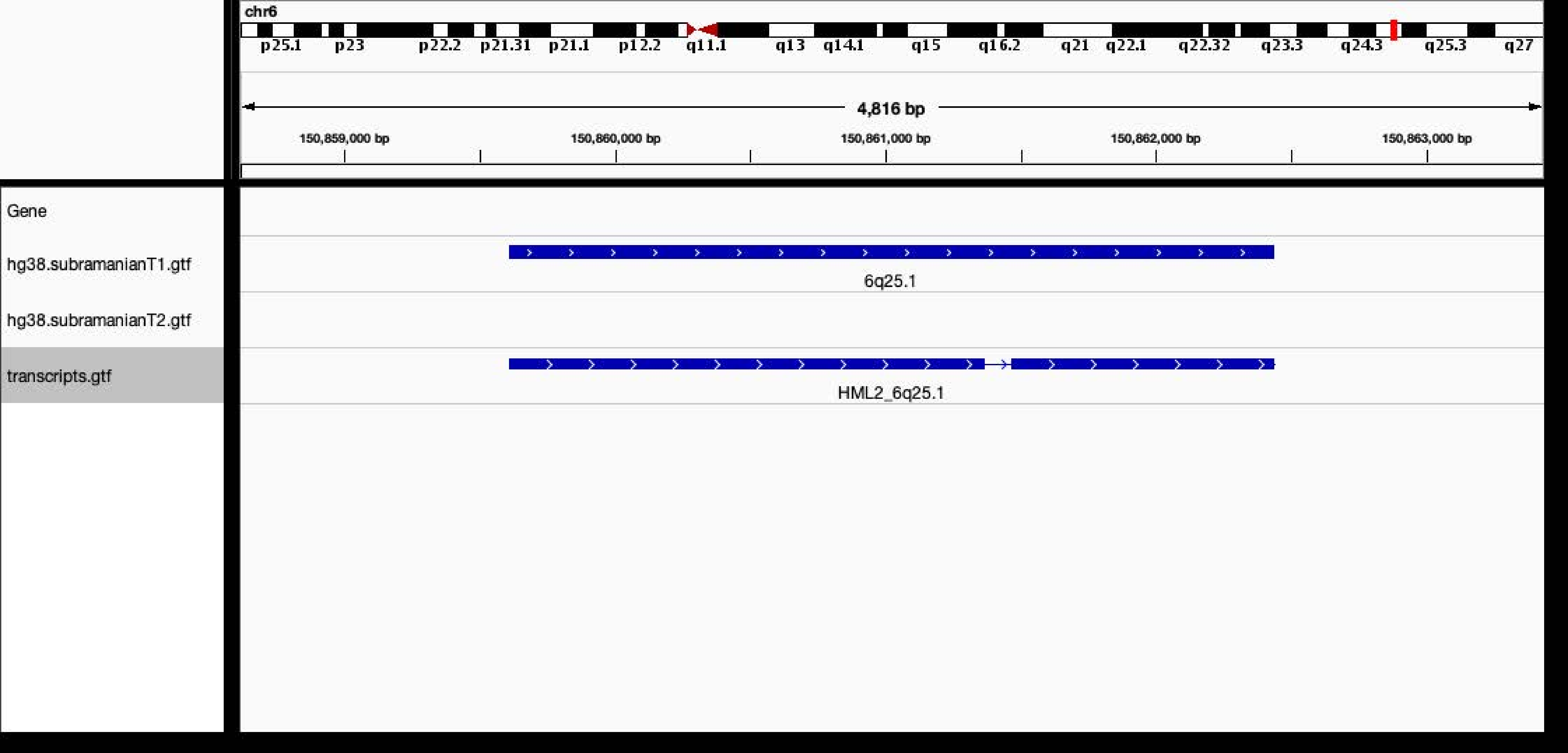
chr6
p25.1 p23 p22.2 p21.31 p21.1 p12.2 q11.1 q13 q14.1 q15 q16.2 q21 q22.1 q22.32 q23.3 q24.3 q25.3 q27
4,816 bp
150,859,000 bp
150,860,000 bp
150,861,000 bp
150,862,000 bp
150,863,000 bp
Gene
hg38.subramanianT1.gtf
hg38.subramanianT2.gtf
transcripts.gtf
6q25.1
HML2_6q25.1

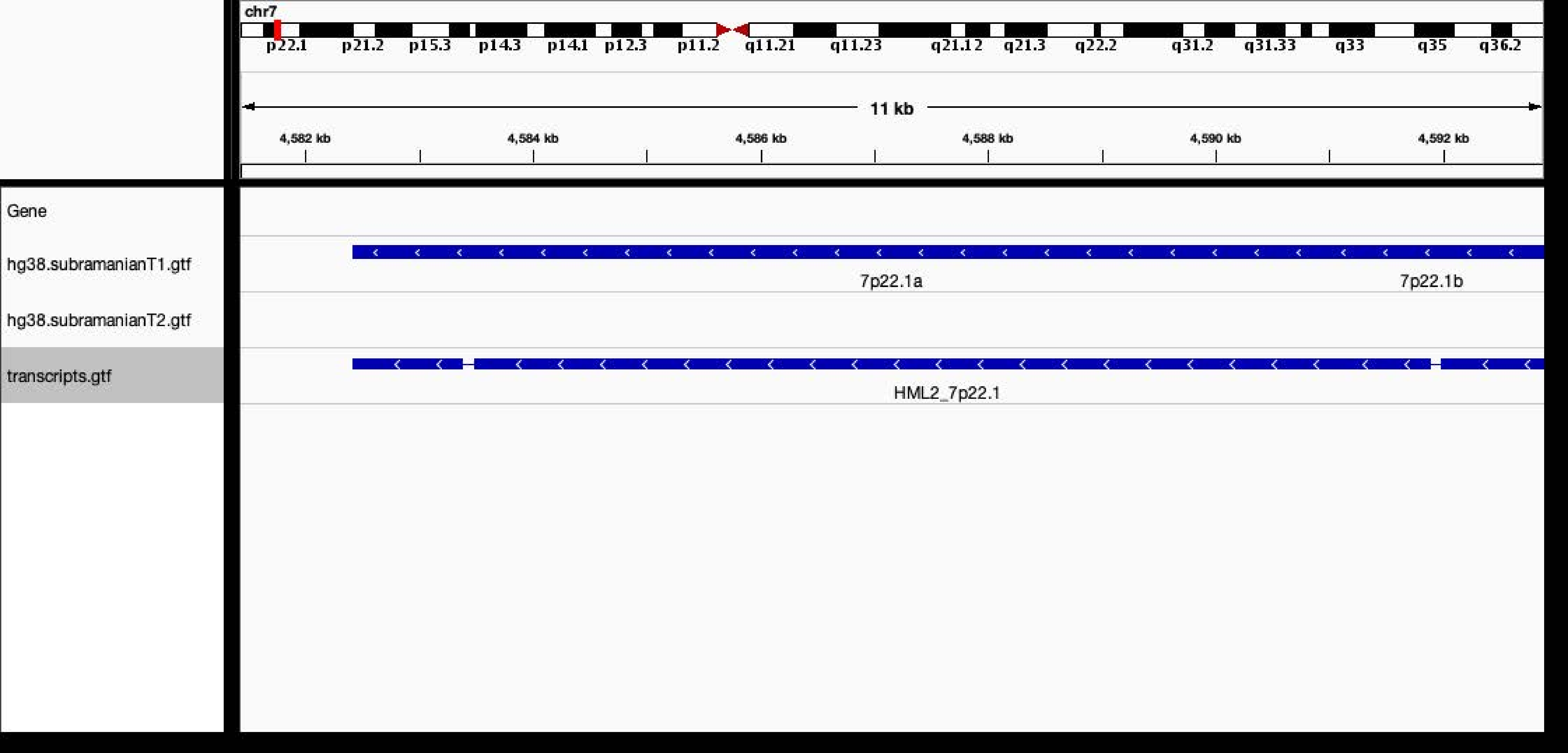
chr7
p22.1
p21.2
p15.3
p14.3
p14.1
p12.3
p11.2
q11.21
q11.23
q21.12
q21.3
q22.2
q31.2
q31.33
q33
q35
q36.2
11 kb
4,582 kb
4,584 kb
4,586 kb
4,588 kb
4,590 kb
4,592 kb
Gene
hg38.subramanianT1.gtf
7p22.1a
7p22.1b
hg38.subramanianT2.gtf
transcripts.gtf
HML2_7p22.1

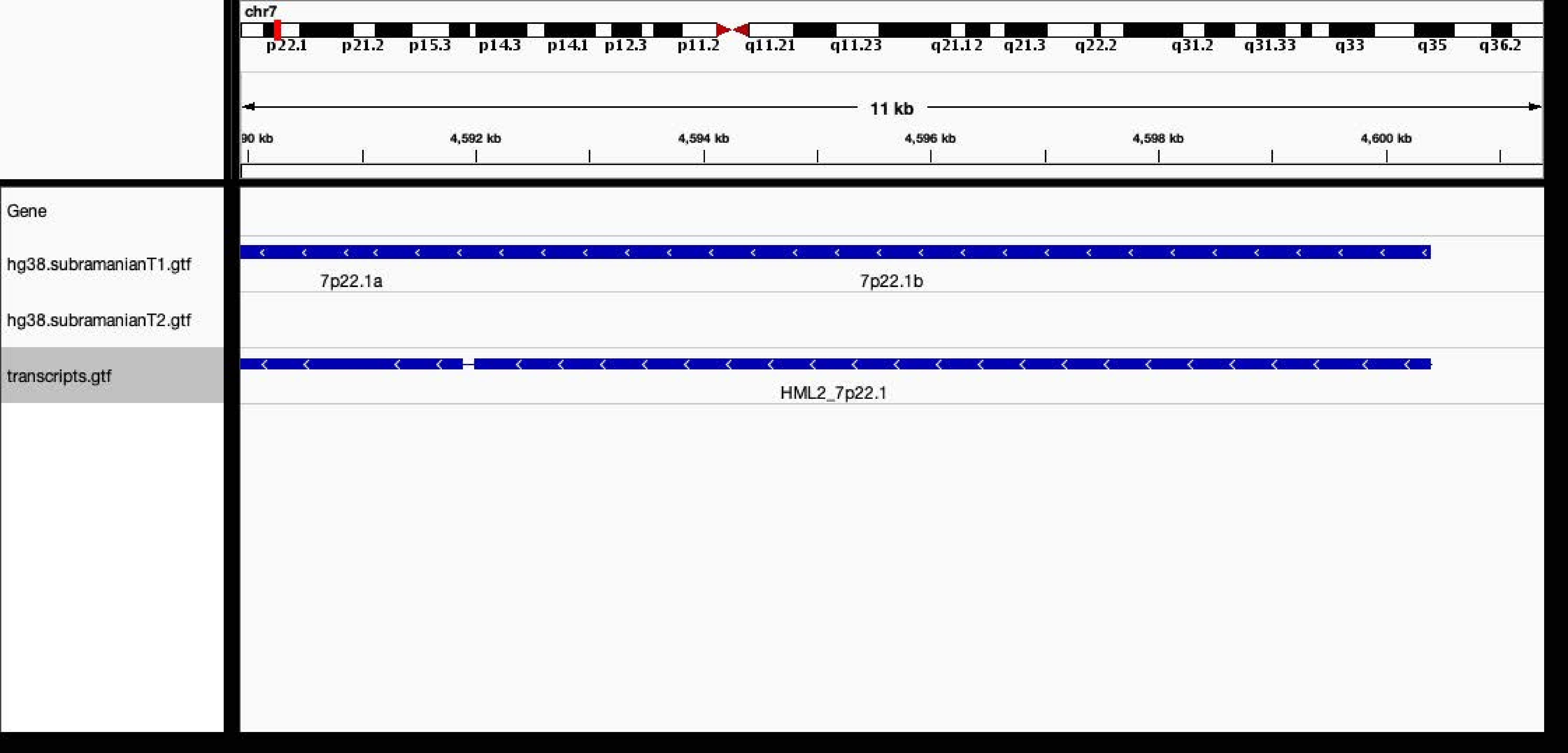
chr7
p22.1
p21.2
p15.3
p14.3
p14.1
p12.3
p11.2
q11.21
q11.23
q21.12
q21.3
q22.2
q31.2
q31.33
q33
q35
q36.2
11 kb
90 kb
4,592 kb
4,594 kb
4,596 kb
4,598 kb
4,600 kb
Gene
hg38.subramanianT1.gtf
7p22.1a
7p22.1b
hg38.subramanianT2.gtf
transcripts.gtf
HML2_7p22.1

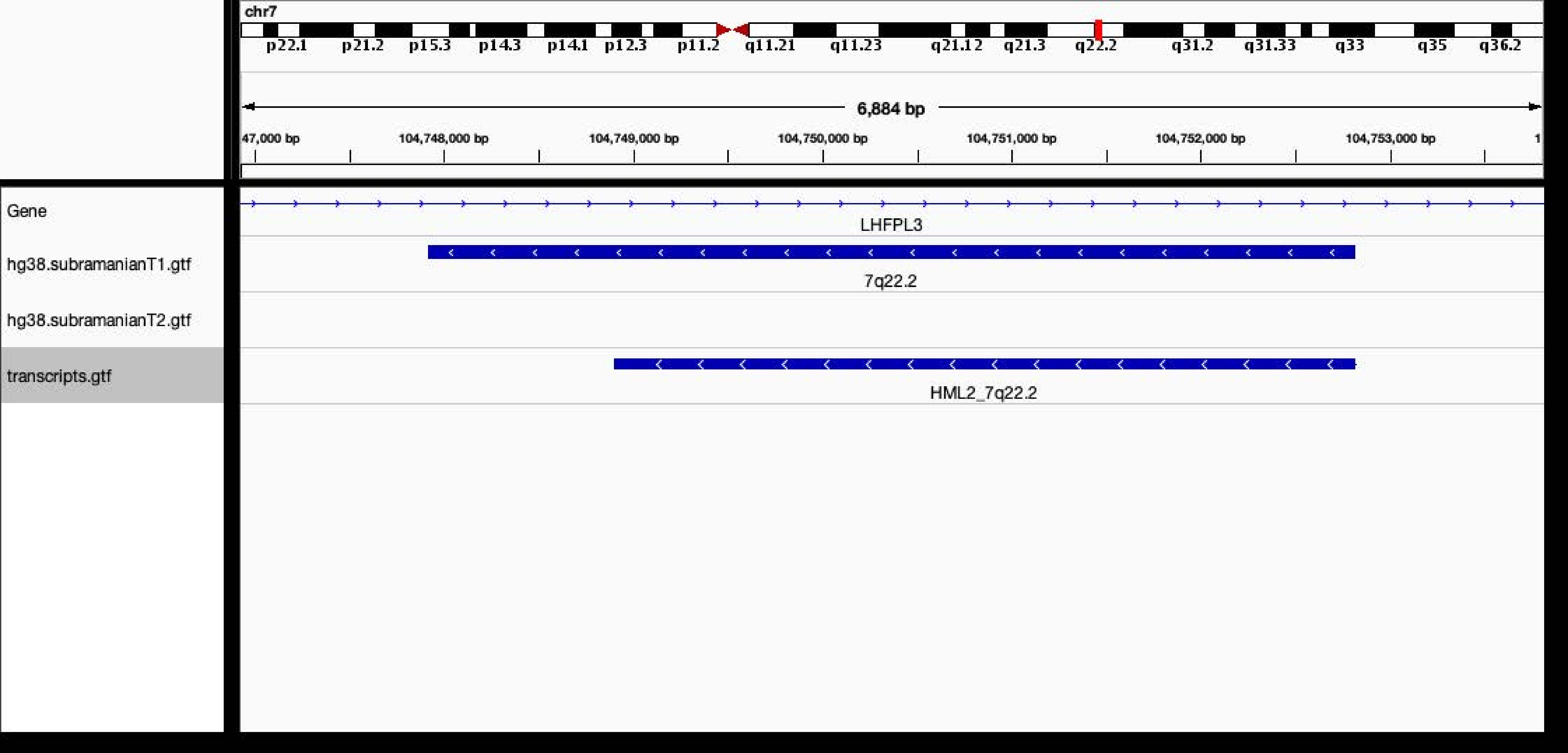
chr7
p22.1
p21.2
p15.3
p14.3
p14.1
p12.3
p11.2
q11.21
q11.23
q21.12
q21.3
q22.2
q31.2
q31.33
q33
q35
q36.2
6,884 bp
47,000 bp
104,748,000 bp
104,749,000 bp
104,750,000 bp
104,751,000 bp
104,752,000 bp
104,753,000 bp
Gene
LHFPL3
hg38.subramanianT1.gtf
7q22.2
hg38.subramanianT2.gtf
transcripts.gtf
HML2_7q22.2

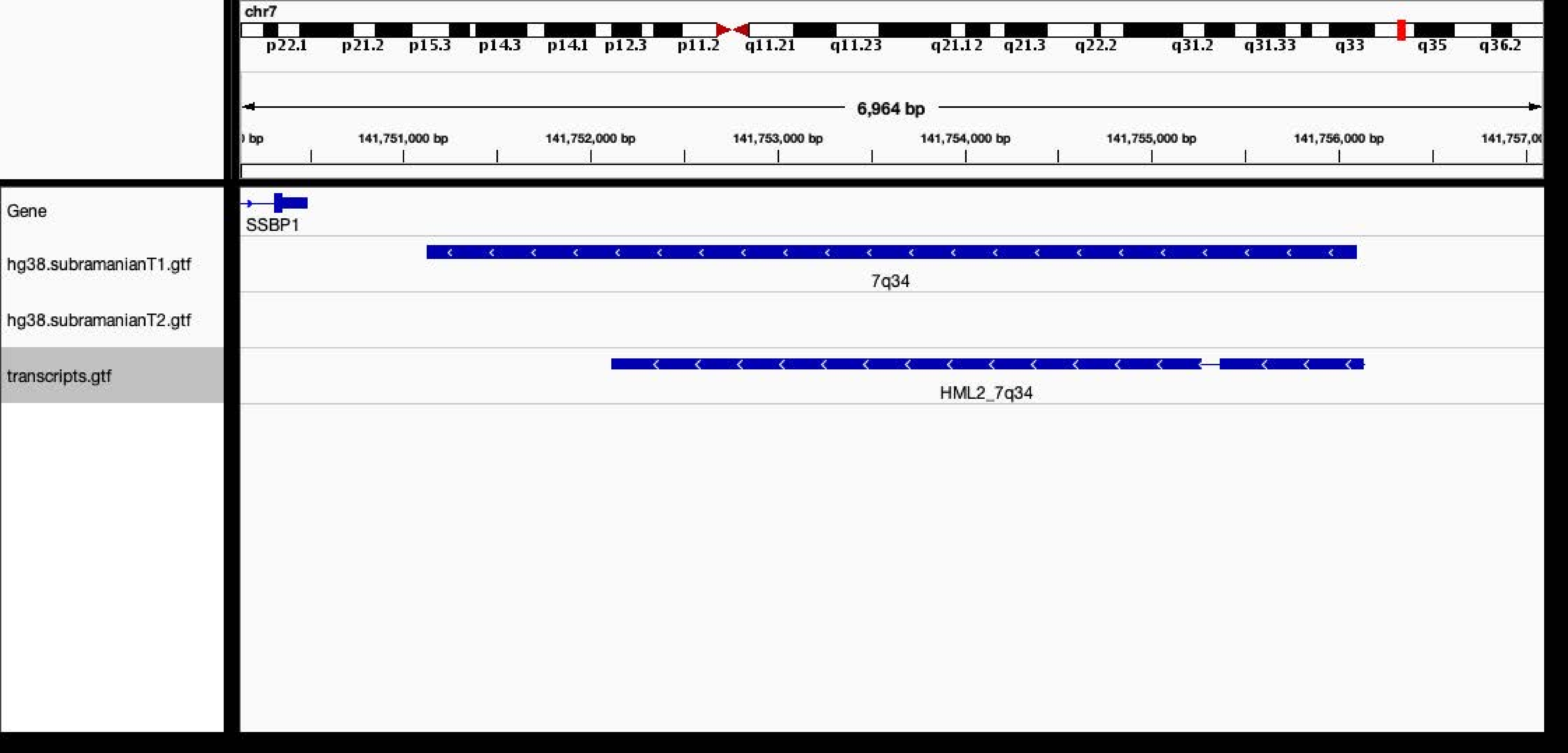
chr7
p22.1
p21.2
p15.3
p14.3
p14.1
p12.3
p11.2
q11.21
q11.23
q21.12
q21.3
q22.2
q31.2
q31.33
q33
q35
q36.2
6,964 bp
141,751,000 bp
141,752,000 bp
141,753,000 bp
141,754,000 bp
141,755,000 bp
141,756,000 bp
Gene
SSBP1
hg38.subramanianT1.gtf
7q34
hg38.subramanianT2.gtf
transcripts.gtf
HML2_7q34

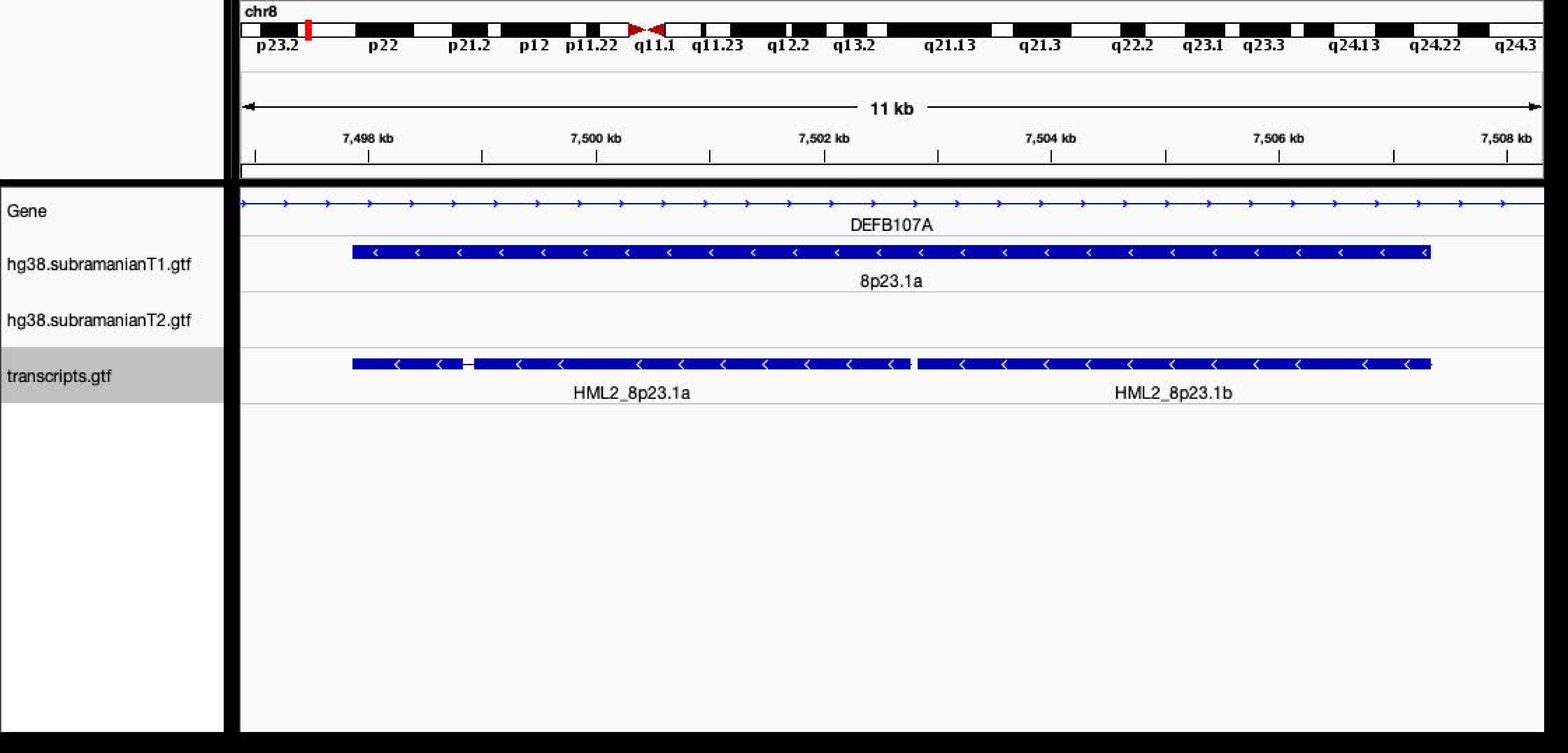
chr8
p23.2
p22
p21.2
p12
p11.22
q11.1
q11.23
q12.2
q13.2
q21.13
q21.3
q22.2
q23.1
q23.3
q24.13
q24.22
q24.3
11 kb
7,498 kb
7,500 kb
7,502 kb
7,504 kb
7,506 kb
7,508 kb
Gene
hg38.subramanianT1.gtf
hg38.subramanianT2.gtf
transcripts.gtf
DEFB107A
8p23.1a
HML2_8p23.1a
HML2_8p23.1b

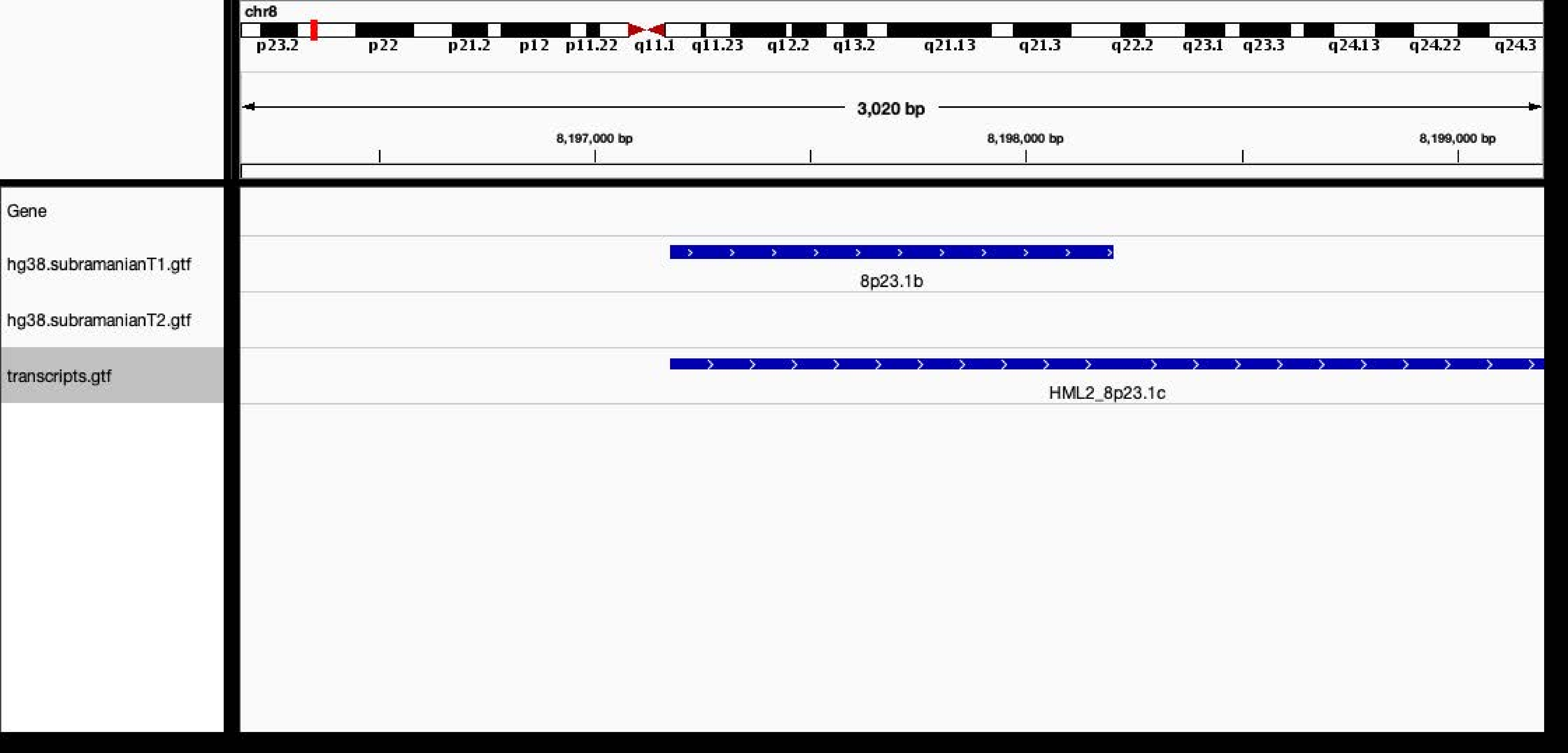
chr8
p23.2
p22
p21.2
p12
p11.22
q11.1
q11.23
q12.2
q13.2
q21.13
q21.3
q22.2
q23.1
q23.3
q24.13
q24.22
q24.3
3,020 bp
8,197,000 bp
8,198,000 bp
8,199,000 bp
Gene
hg38.subramanianT1.gtf
8p23.1b
hg38.subramanianT2.gtf
transcripts.gtf
HML2_8p23.1c

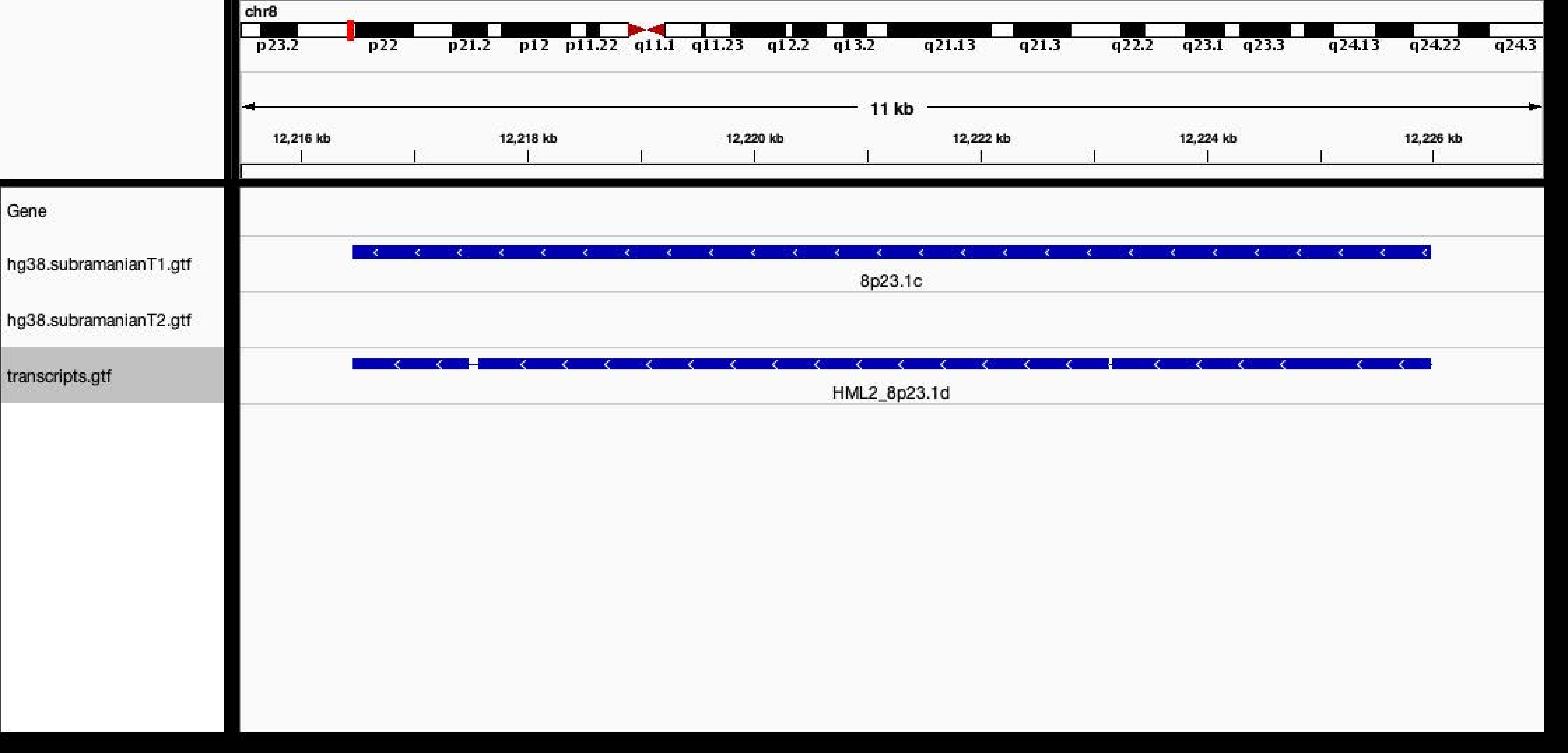

chr8
p23.2
p22
p21.2
p12
p11.22
q11.1
q11.23
q12.2
q13.2
q21.13
q21.3
q22.2
q23.1
q23.3
q24.13
q24.22
q24.3
11 kb
12,216 kb
12,218 kb
12,220 kb
12,222 kb
12,224 kb
12,226 kb
Gene
hg38.subramanianT1.gtf
8p23.1c
hg38.subramanianT2.gtf
transcripts.gtf
HML2_8p23.1d

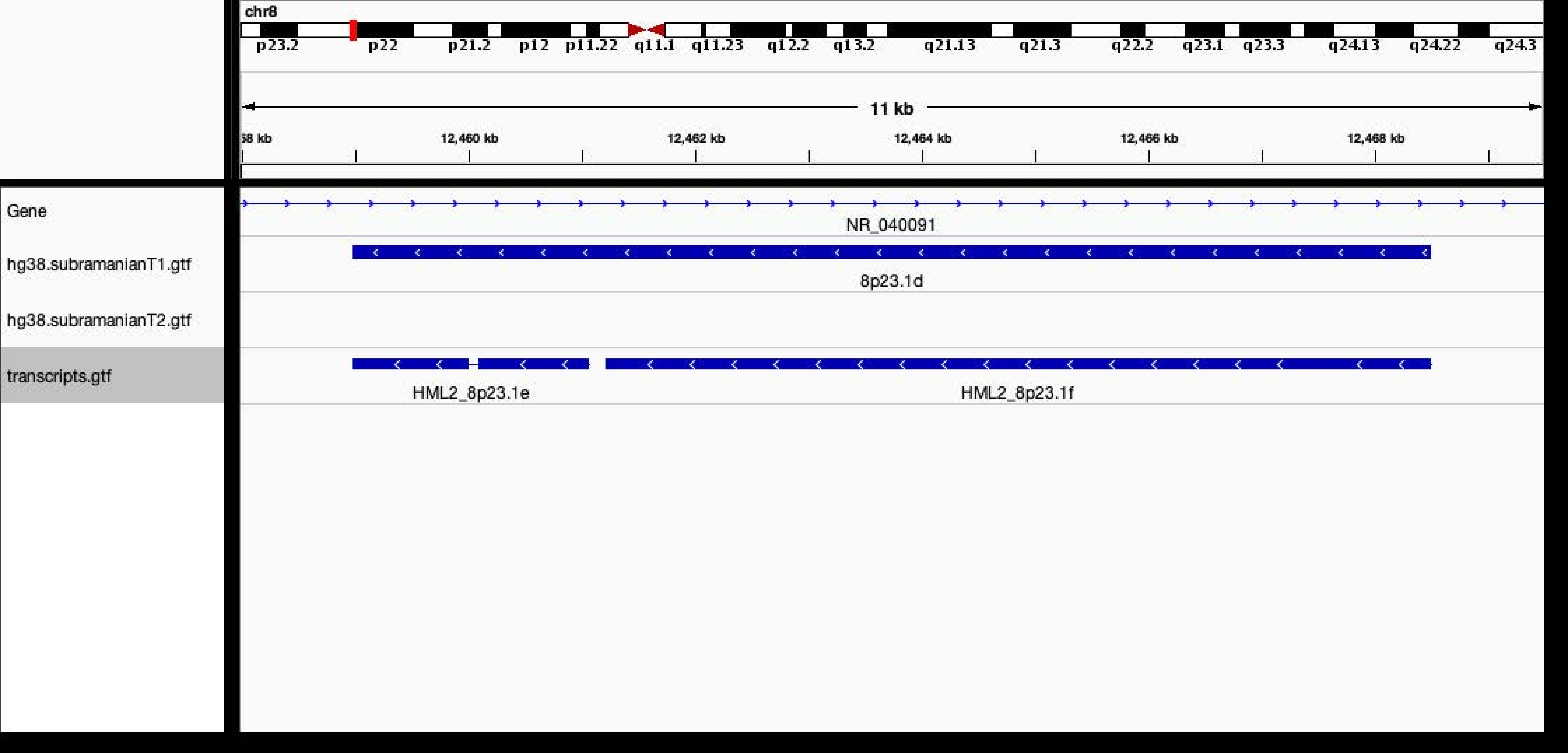

chr8
p23.2
p22
p21.2
p12
p11.22
q11.1
q11.23
q12.2
q13.2
q21.13
q21.3
q22.2
q23.1
q23.3
q24.13
q24.22
q24.3
11 kb
12,460 kb
12,462 kb
12,464 kb
12,466 kb
12,468 kb
Gene
NR_040091
hg38.subramanianT1.gtf
8p23.1d
hg38.subramanianT2.gtf
transcripts.gtf
HML2_8p23.1e
HML2_8p23.1f

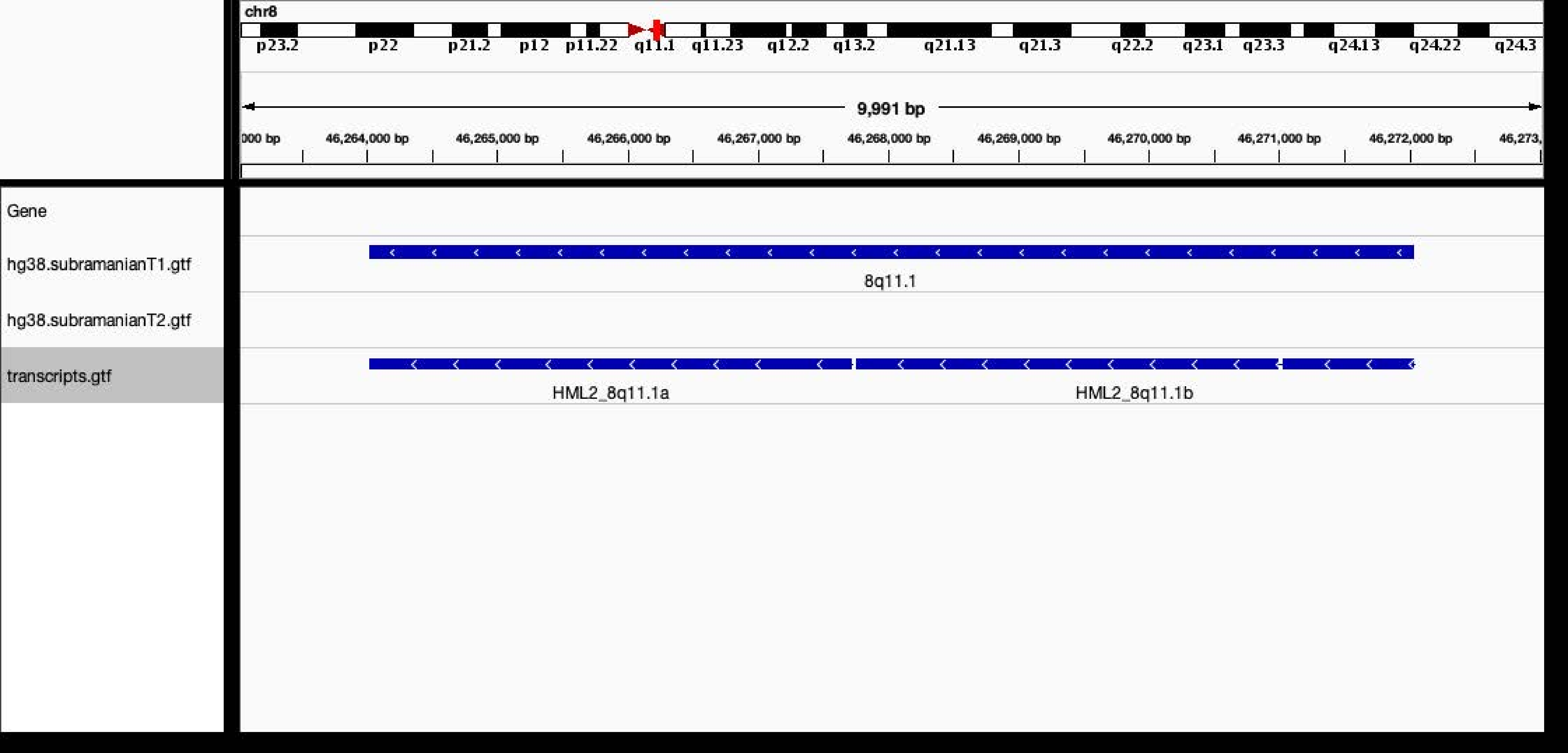
chr8
p23.2
p22
p21.2
p12
p11.22
q11.1
q11.23
q12.2
q13.2
q21.13
q21.3
q22.2
q23.1
q23.3
q24.13
q24.22
q24.3
9,991 bp
46,264,000 bp
46,265,000 bp
46,266,000 bp
46,267,000 bp
46,268,000 bp
46,269,000 bp
46,270,000 bp
46,271,000 bp
46,272,000 bp
Gene
hg38.subramanianT1.gtf
8q11.1
hg38.subramanianT2.gtf
transcripts.gtf
HML2_8q11.1a
HML2_8q11.1b

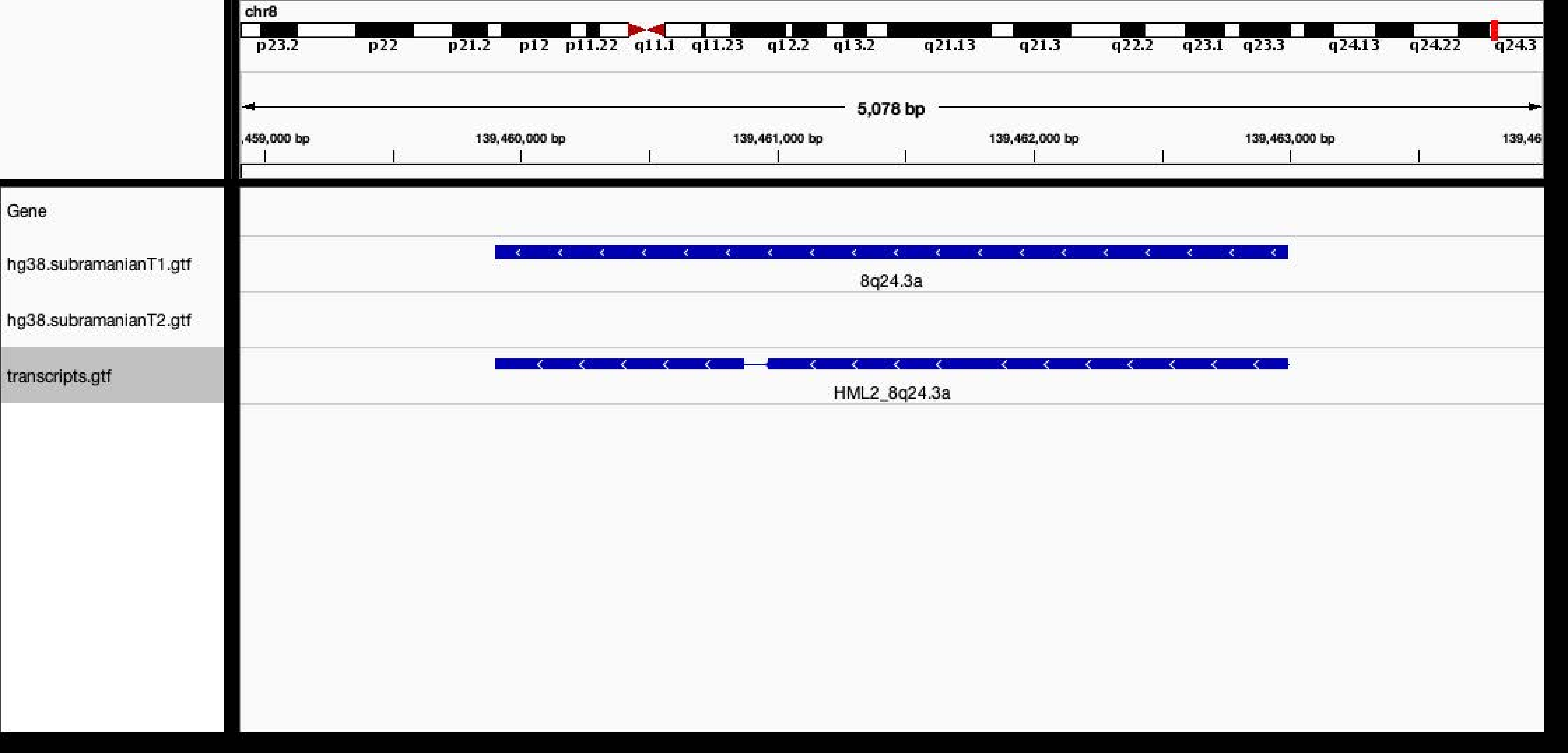

chr8
p23.2
p22
p21.2
p12
p11.22
q11.1
q11.23
q12.2
q13.2
q21.13
q21.3
q22.2
q23.1
q23.3
q24.13
q24.22
q24.3
5,078 bp
,459,000 bp
139,460,000 bp
139,461,000 bp
139,462,000 bp
139,463,000 bp
139,46
Gene
hg38.subramanianT1.gtf
hg38.subramanianT2.gtf
transcripts.gtf
8q24.3a
HML2_8q24.3a

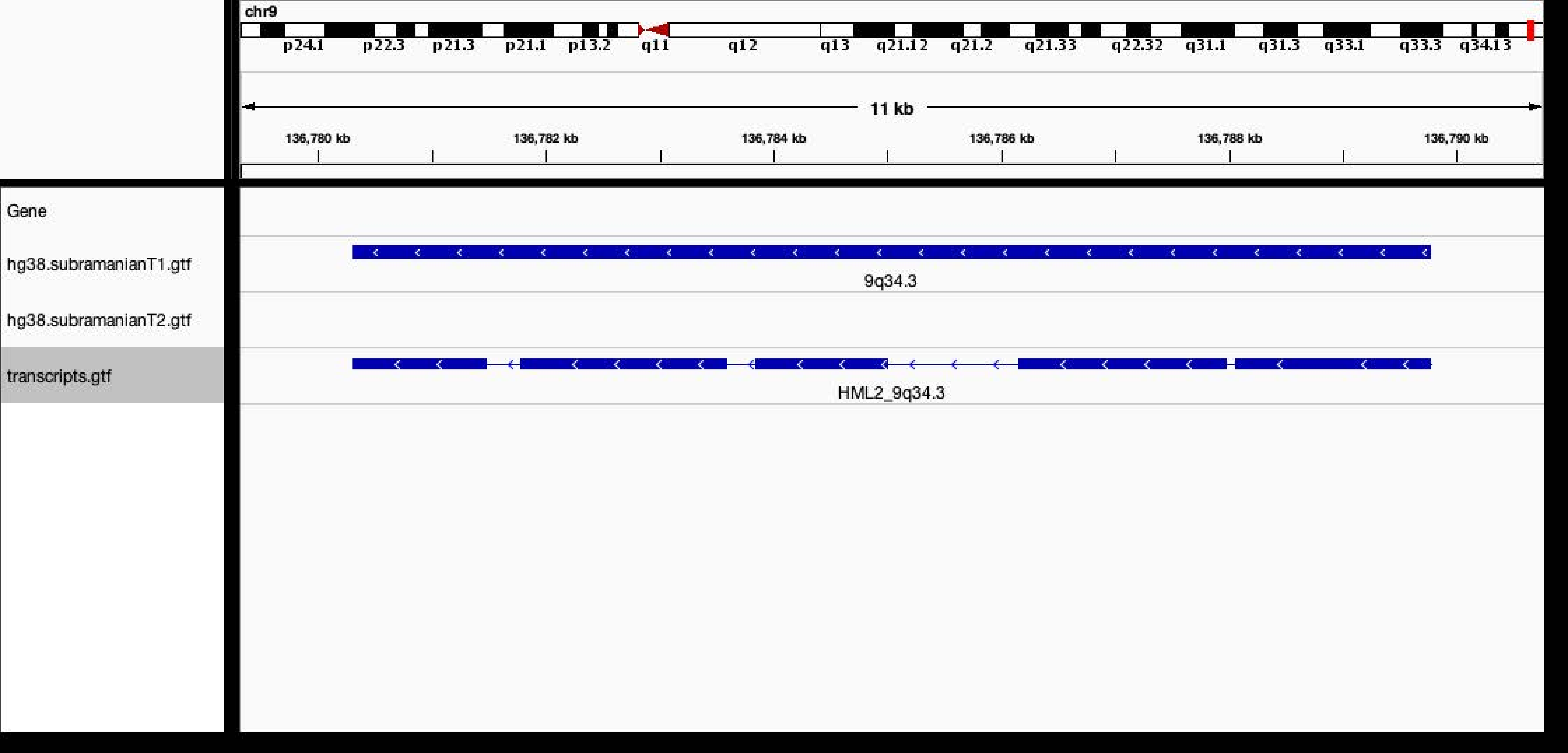
chr9
p24.1
p22.3
p21.3
p21.1
p13.2
q11
q12
q13
q21.12
q21.2
q21.33
q22.32
q31.1
q31.3
q33.1
q33.3
q34.13
11 kb
136,780 kb
136,782 kb
136,784 kb
136,786 kb
136,788 kb
136,790 kb
Gene
hg38.subramanianT1.gtf
9q34.3
hg38.subramanianT2.gtf
transcripts.gtf
HML2_9q34.3

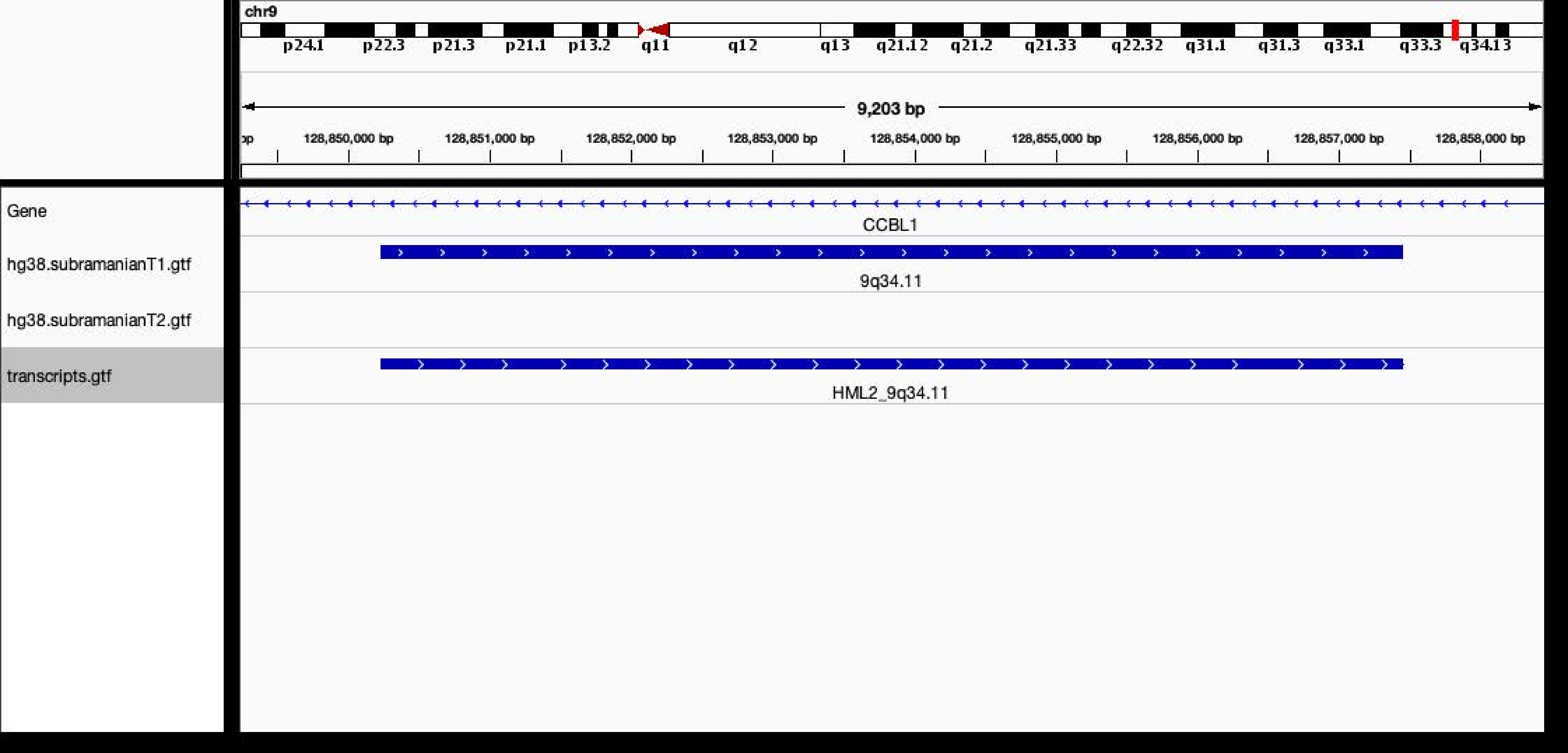

chr9
p24.1
p22.3
p21.3
p21.1
p13.2
q11
q12
q13
q21.12
q21.2
q21.33
q22.32
q31.1
q31.3
q33.1
q33.3
q34.13
9,203 bp
128,850,000 bp
128,851,000 bp
128,852,000 bp
128,853,000 bp
128,854,000 bp
128,855,000 bp
128,856,000 bp
128,857,000 bp
128,858,000 bp
Gene
CCBL1
hg38.subramanianT1.gtf
9q34.11
hg38.subramanianT2.gtf
transcripts.gtf
HML2_9q34.11

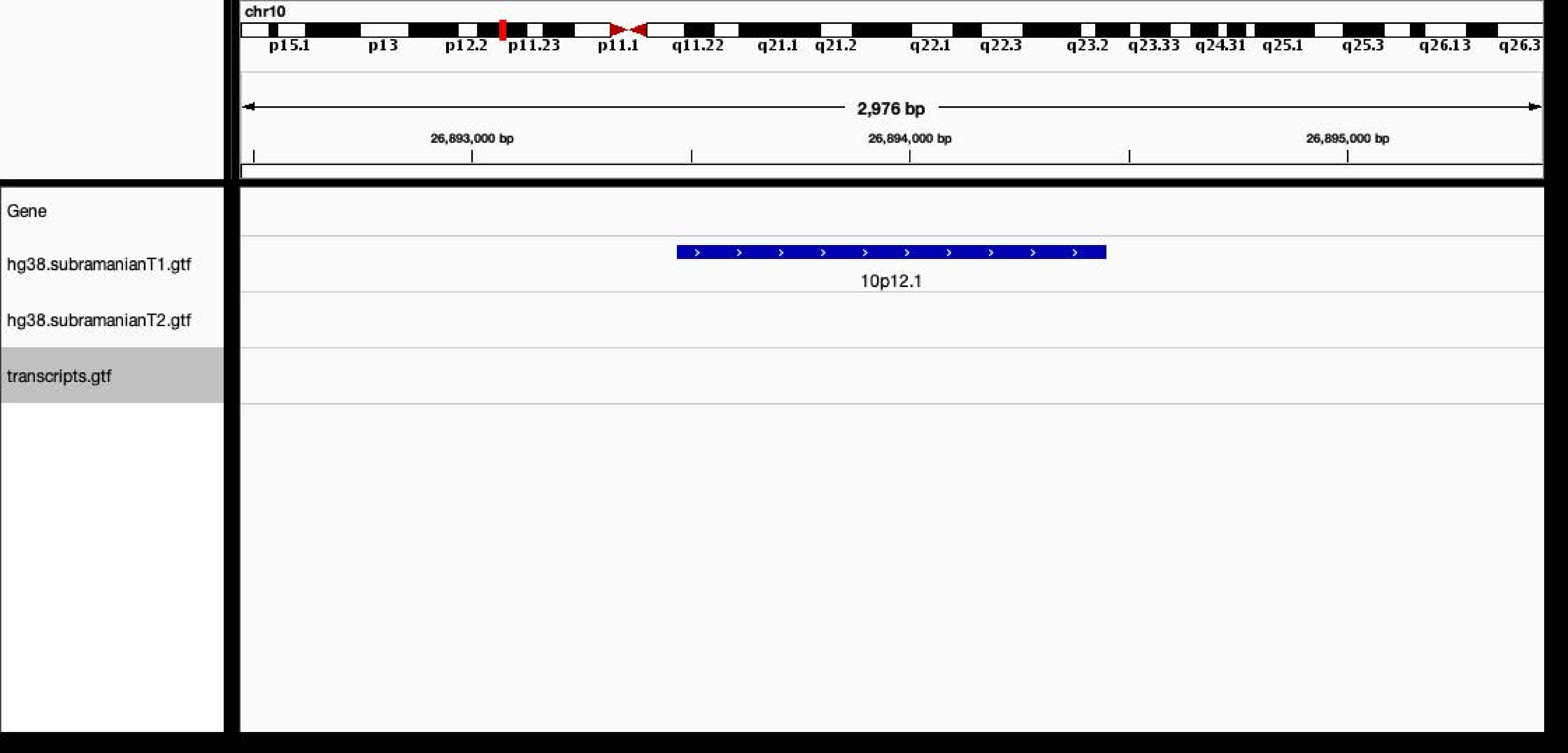
chr10
p15.1
p13
p12.2
p11.23
p11.1
q11.22
q21.1
q21.2
q22.1
q22.3
q23.2
q23.33
q24.31
q25.1
q25.3
q26.13
q26.3
2,976 bp
26,893,000 bp
26,894,000 bp
26,895,000 bp
Gene
hg38.subramanianT1.gtf
10p12.1
hg38.subramanianT2.gtf
transcripts.gtf

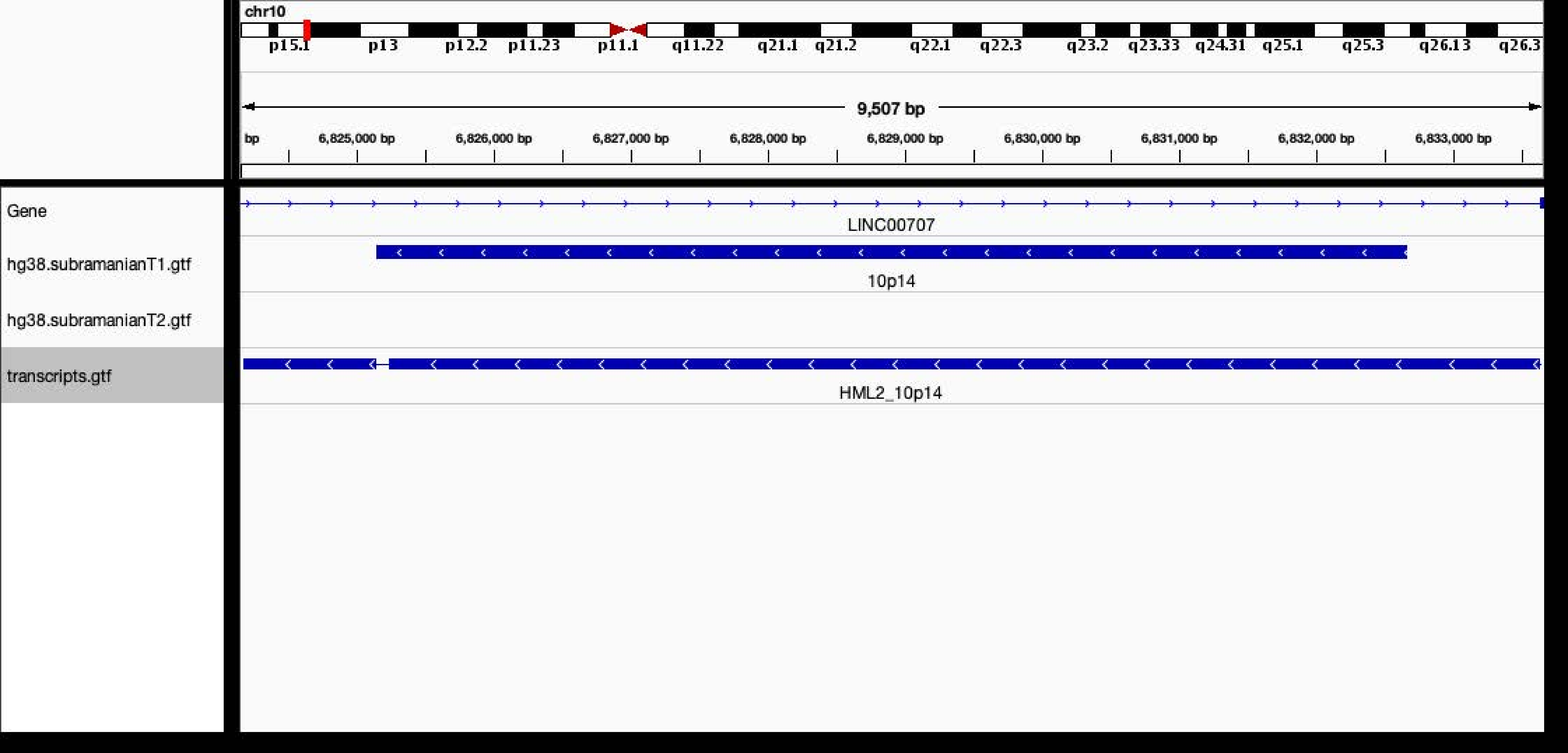

chr10
p15.1
p13
p12.2
p11.23
p11.1
q11.22
q21.1
q21.2
q22.1
q22.3
q23.2
q23.33
q24.31
q25.1
q25.3
q26.13
q26.3
9,507 bp
bp
6,825,000 bp
6,826,000 bp
6,827,000 bp
6,828,000 bp
6,829,000 bp
6,830,000 bp
6,831,000 bp
6,832,000 bp
6,833,000 bp
Gene
LINC00707
hg38.subramanianT1.gtf
10p14
hg38.subramanianT2.gtf
transcripts.gtf
HML2_10p14

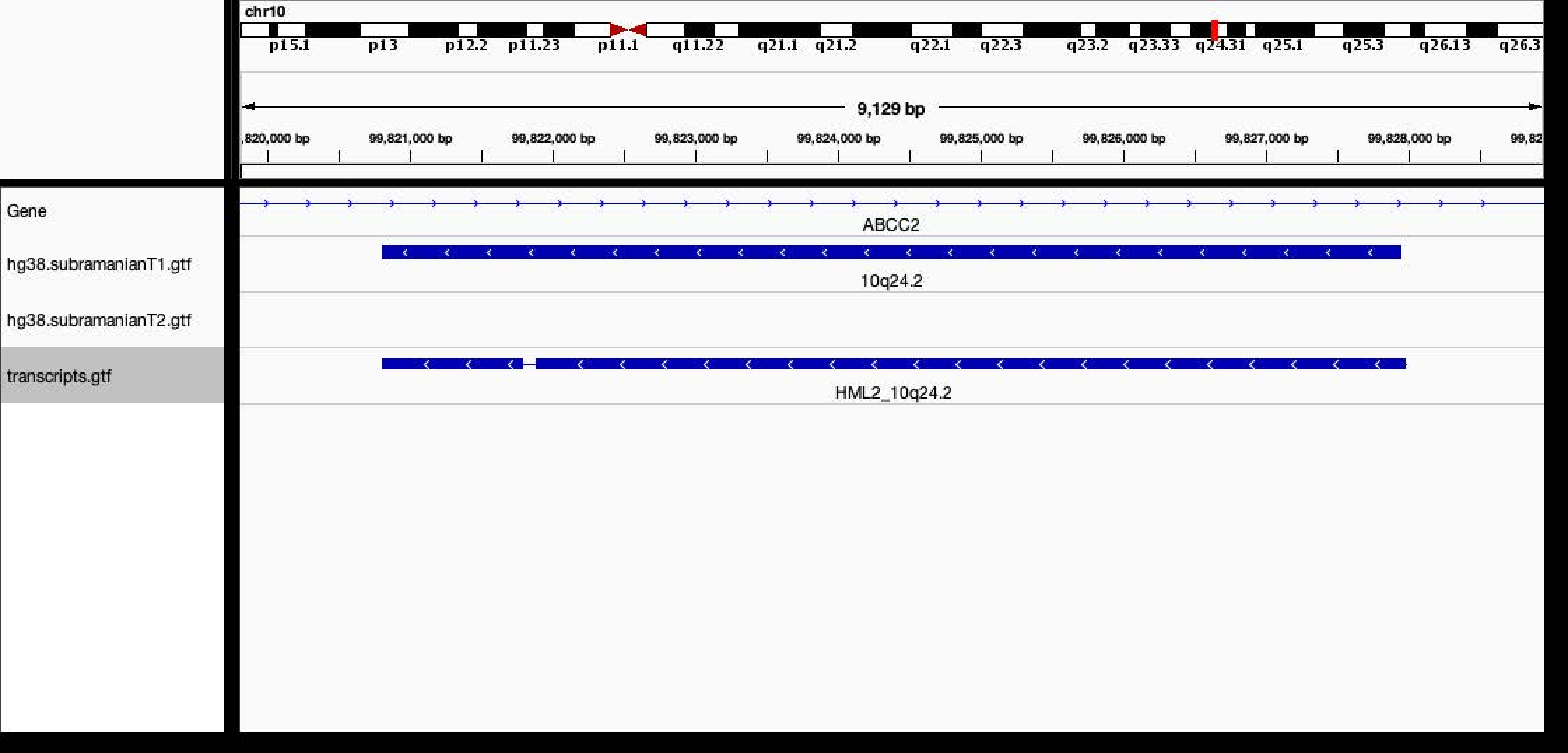
chr10
p15.1 p13 p12.2 p11.23 p11.1 q11.22 q21.1 q21.2 q22.1 q22.3 q23.2 q23.33 q24.31 q25.1 q25.3 q26.13 q26.3
9,129 bp
,820,000 bp 99,821,000 bp 99,822,000 bp 99,823,000 bp 99,824,000 bp 99,825,000 bp 99,826,000 bp 99,827,000 bp 99,828,000 bp
Gene
ABCC2
hg38.subramanianT1.gtf
10q24.2
hg38.subramanianT2.gtf
transcripts.gtf
HML2_10q24.2

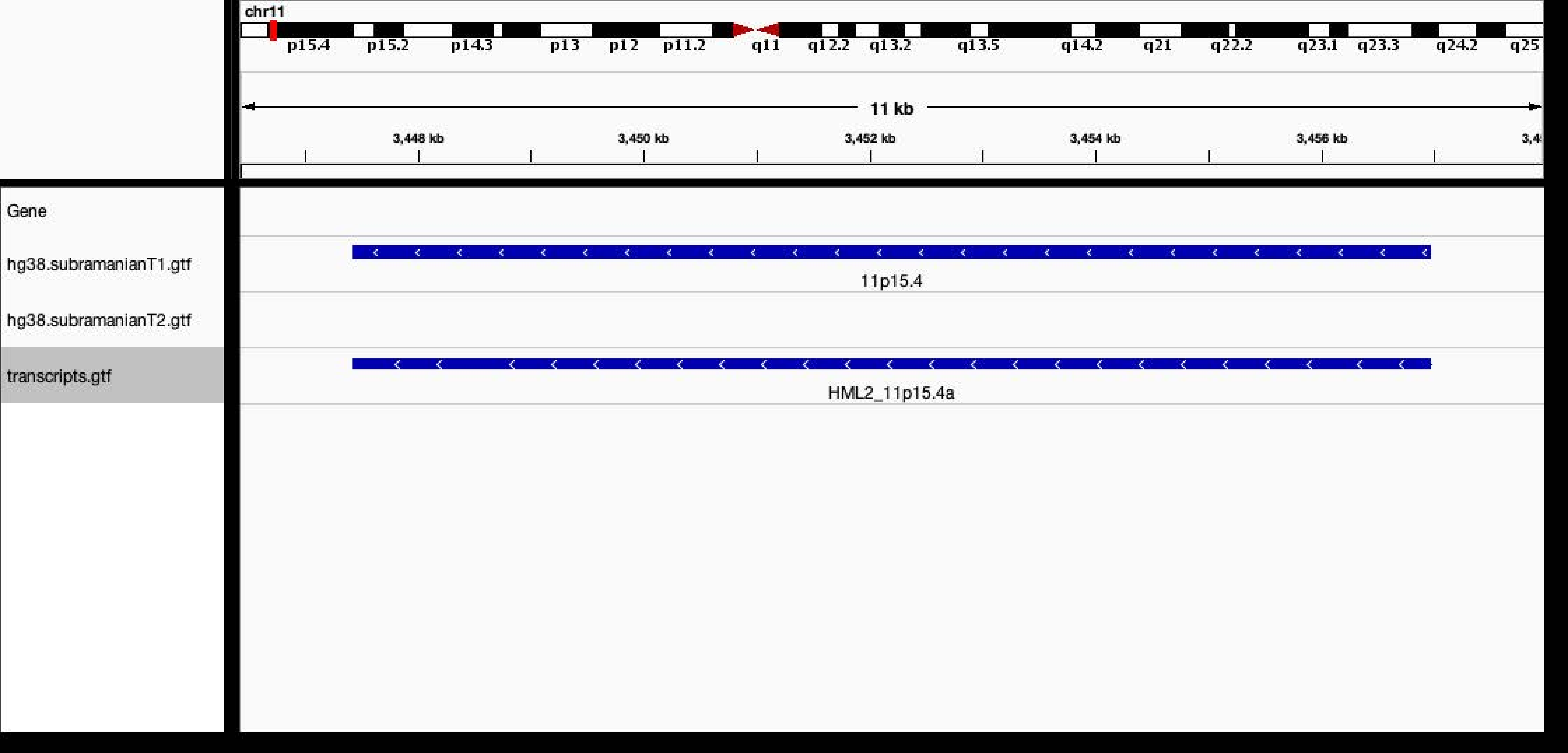
chr11
p15.4
p15.2
p14.3
p13
p12
p11.2
q11
q12.2
q13.2
q13.5
q14.2
q21
q22.2
q23.1
q23.3
q24.2
q25
11 kb
3,448 kb
3,450 kb
3,452 kb
3,454 kb
3,456 kb
Gene
hg38.subramanianT1.gtf
11p15.4
hg38.subramanianT2.gtf
transcripts.gtf
HML2_11p15.4a

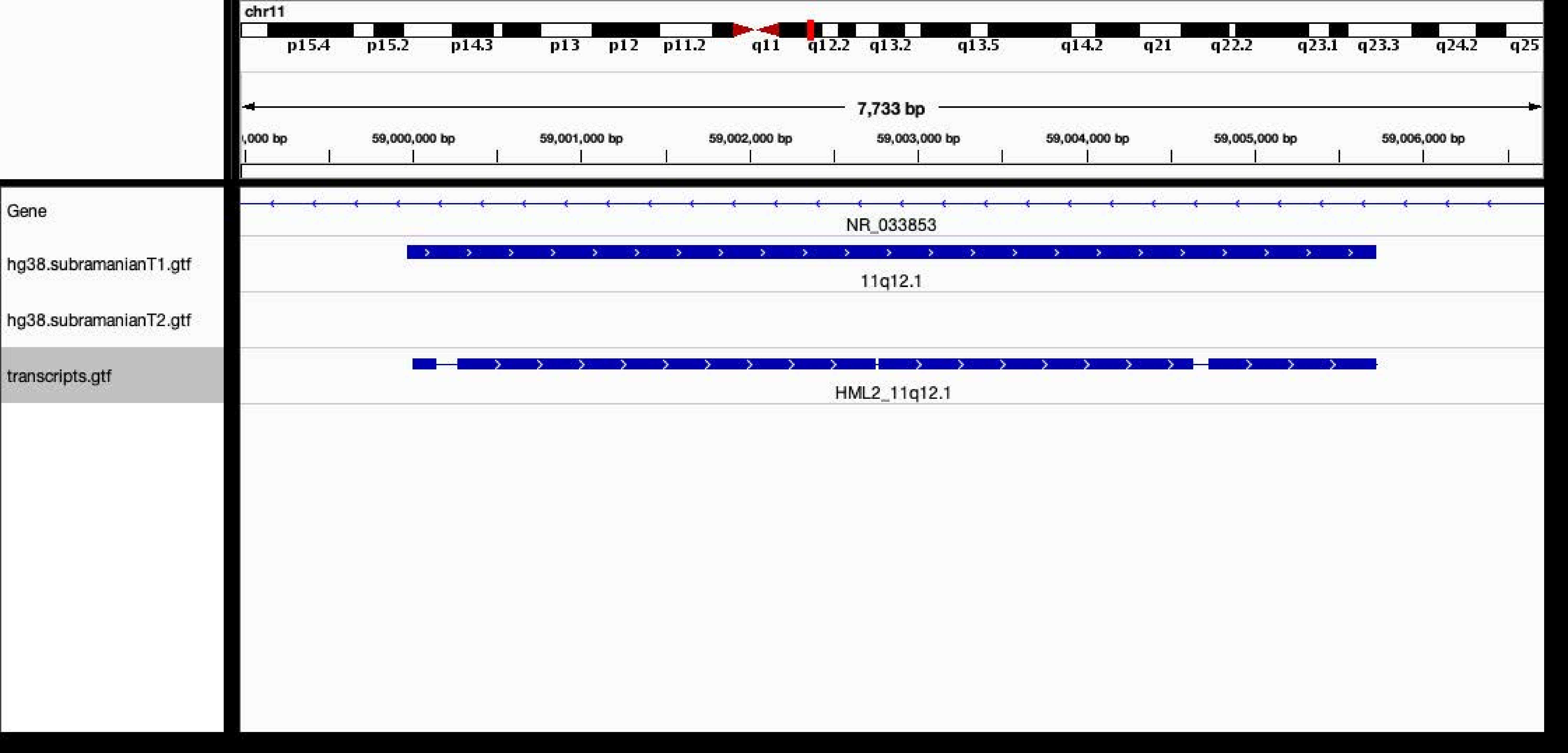
chr11
p15.4
p15.2
p14.3
p13
p12
p11.2
q11
q12.2
q13.2
q13.5
q14.2
q21
q22.2
q23.1
q23.3
q24.2
q25
7,733 bp
,000 bp
59,000,000 bp
59,001,000 bp
59,002,000 bp
59,003,000 bp
59,004,000 bp
59,005,000 bp
59,006,000 bp
Gene
NR_033853
hg38.subramanianT1.gtf
11q12.1
hg38.subramanianT2.gtf
transcripts.gtf
HML2_11q12.1

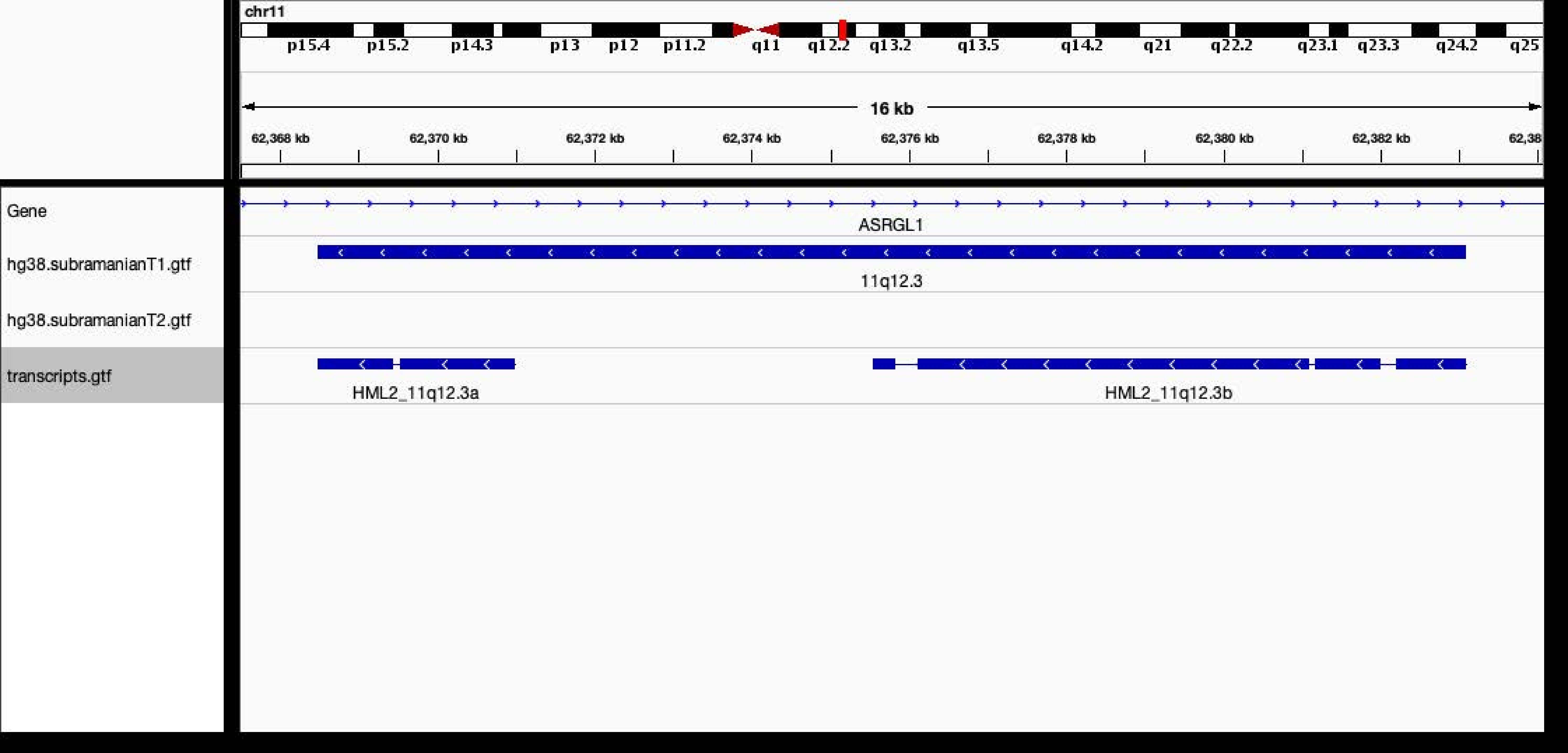

chr11
p15.4
p15.2
p14.3
p13
p12
p11.2
q11
q12.2
q13.2
q13.5
q14.2
q21
q22.2
q23.1
q23.3
q24.2
q25
16 kb
62,368 kb
62,370 kb
62,372 kb
62,374 kb
62,376 kb
62,378 kb
62,380 kb
62,382 kb
Gene
ASRGL1
hg38.subramanianT1.gtf
11q12.3
hg38.subramanianT2.gtf
transcripts.gtf
HML2_11q12.3a
HML2_11q12.3b

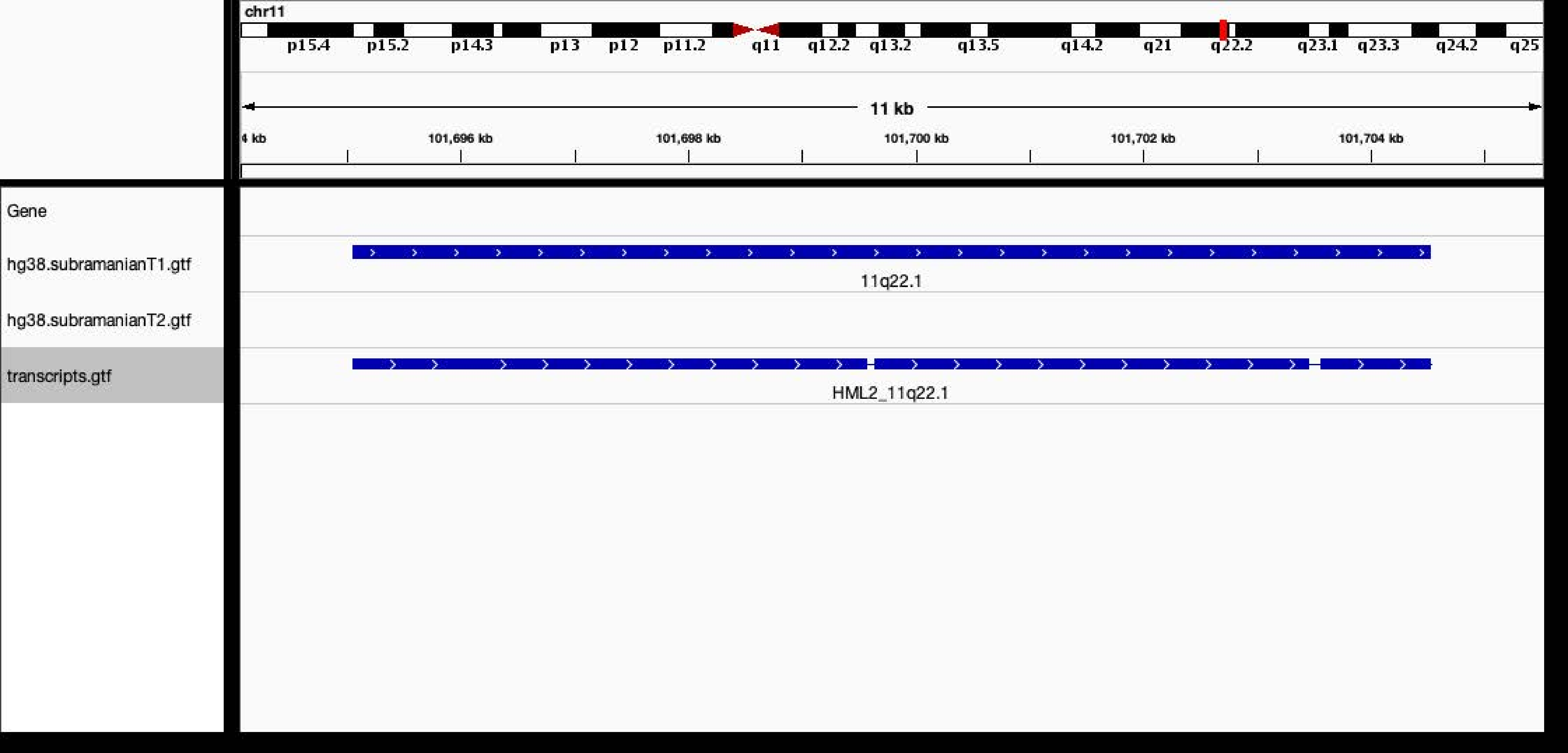

chr11
p15.4
p15.2
p14.3
p13
p12
p11.2
q11
q12.2
q13.2
q13.5
q14.2
q21
q22.2
q23.1
q23.3
q24.2
q25
11 kb
4 kb
101,696 kb
101,698 kb
101,700 kb
101,702 kb
101,704 kb
Gene
hg38.subramanianT1.gtf
11q22.1
hg38.subramanianT2.gtf
transcripts.gtf
HML2_11q22.1

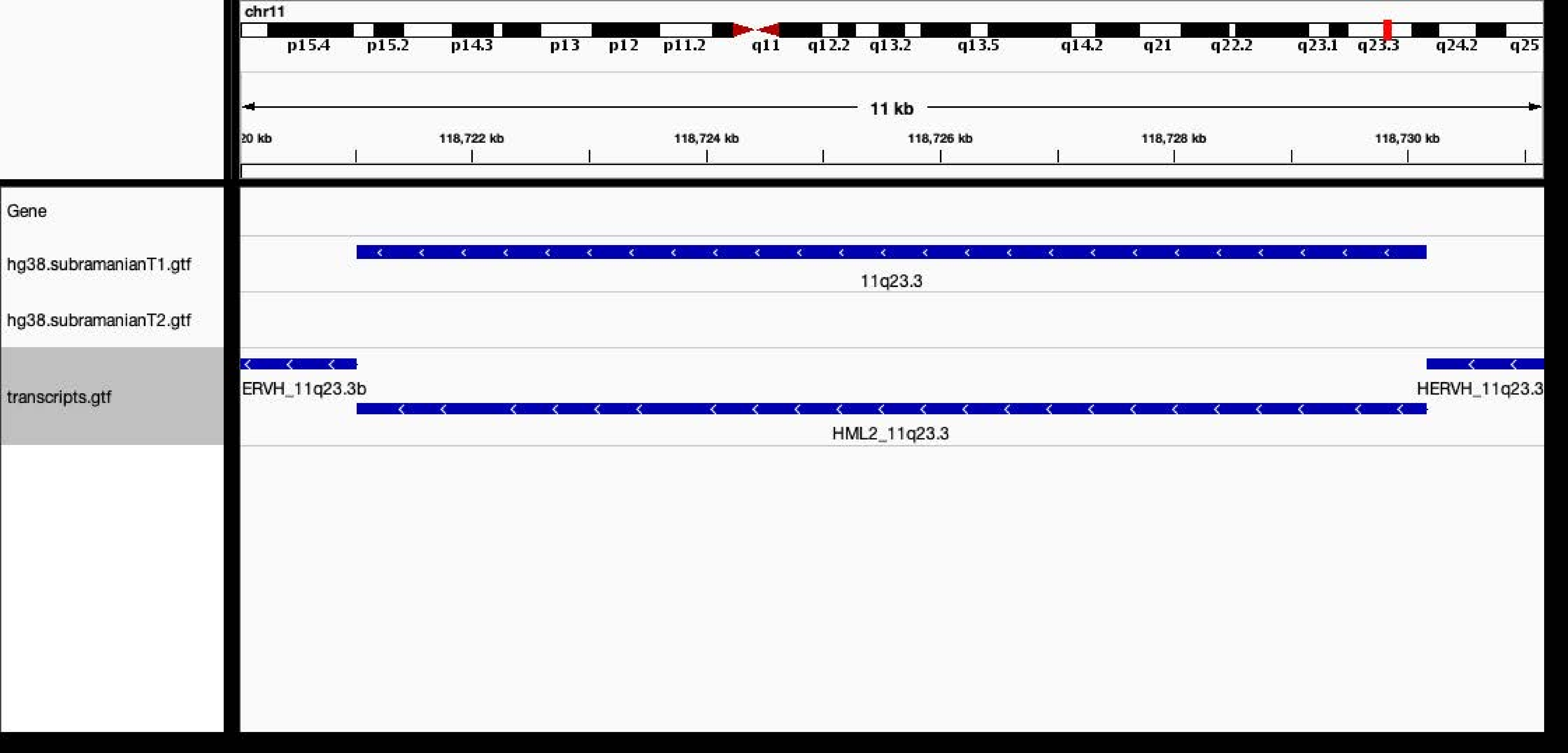

chr11
p15.4
p15.2
p14.3
p13
p12
p11.2
q11
q12.2
q13.2
q13.5
q14.2
q21
q22.2
q23.1
q23.3
q24.2
q25
11 kb
20 kb
118,722 kb
118,724 kb
118,726 kb
118,728 kb
118,730 kb
Gene
hg38.subramanianT1.gtf
11q23.3
hg38.subramanianT2.gtf
transcripts.gtf
ERVH_11q23.3b
HERVH_11q23.3
HML2_11q23.3

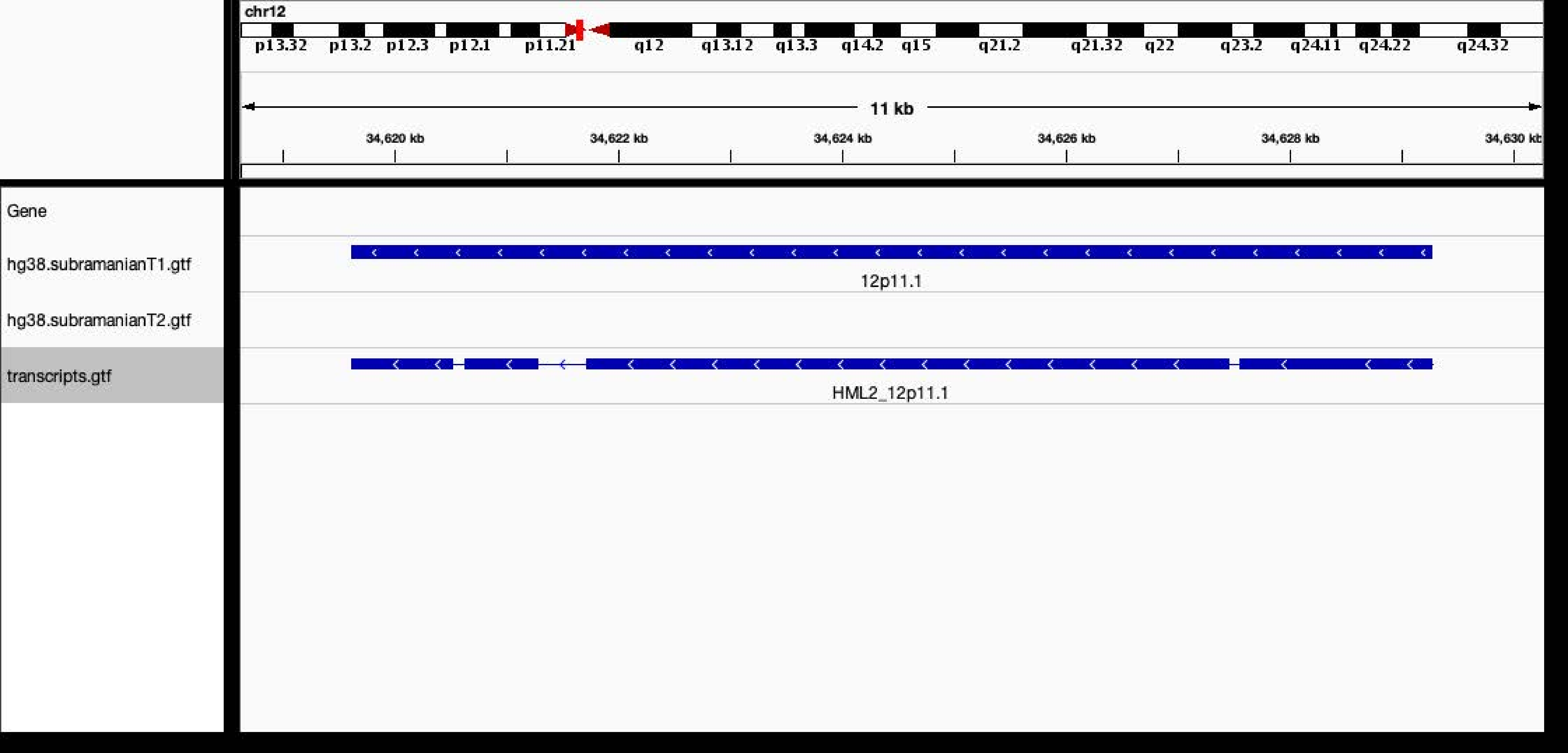
chr12
p13.32
p13.2
p12.3
p12.1
p11.21
q12
q13.12
q13.3
q14.2
q15
q21.2
q21.32
q22
q23.2
q24.11
q24.22
q24.32
11 kb
34,620 kb
34,622 kb
34,624 kb
34,626 kb
34,628 kb
34,630 kb
Gene
hg38.subramanianT1.gtf
12p11.1
hg38.subramanianT2.gtf
transcripts.gtf
HML2_12p11.1

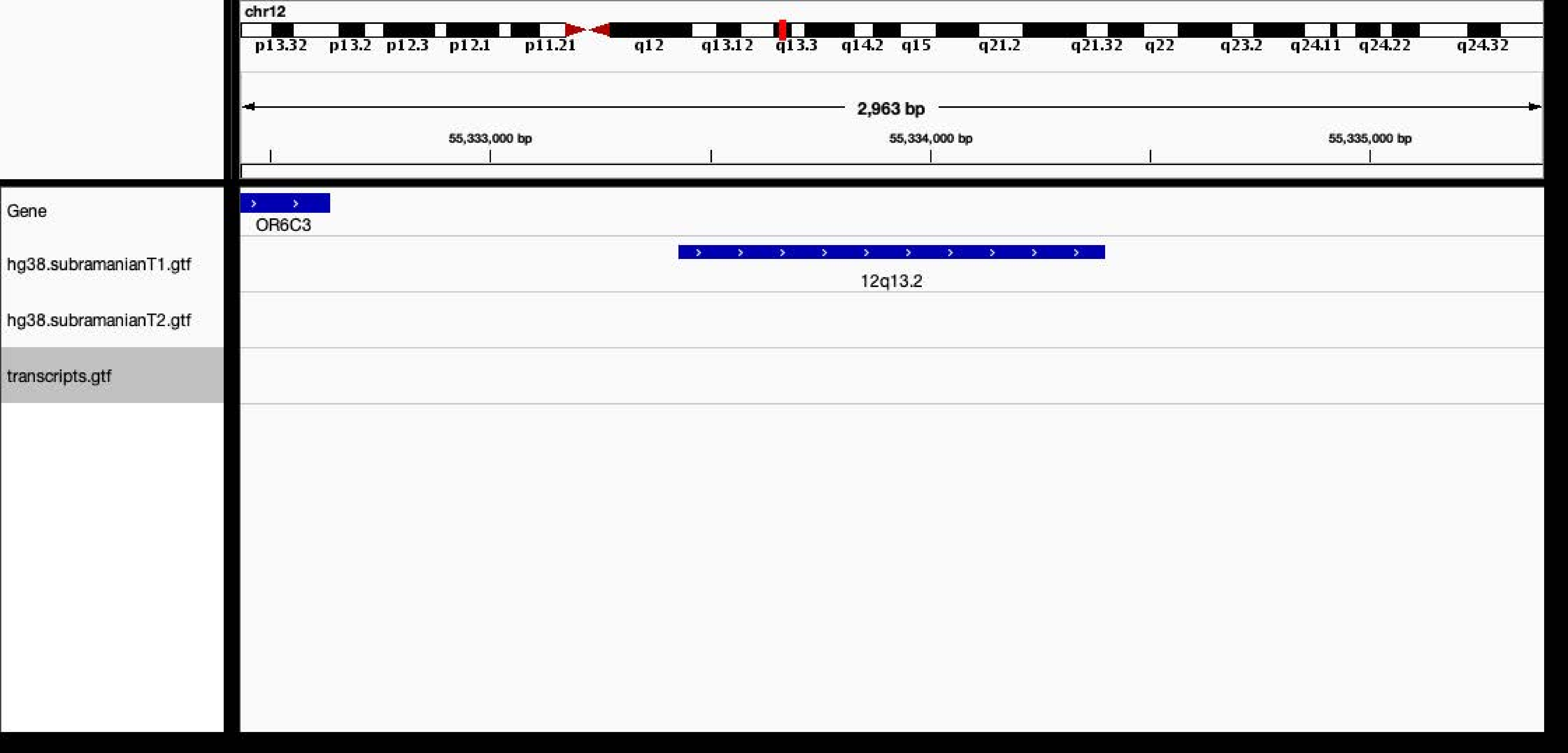
chr12
p13.32
p13.2
p12.3
p12.1
p11.21
q12
q13.12
q13.3
q14.2
q15
q21.2
q21.32
q22
q23.2
q24.11
q24.22
q24.32
2,963 bp
55,333,000 bp
55,334,000 bp
55,335,000 bp
Gene
OR6C3
hg38.subramanianT1.gtf
12q13.2
hg38.subramanianT2.gtf
transcripts.gtf

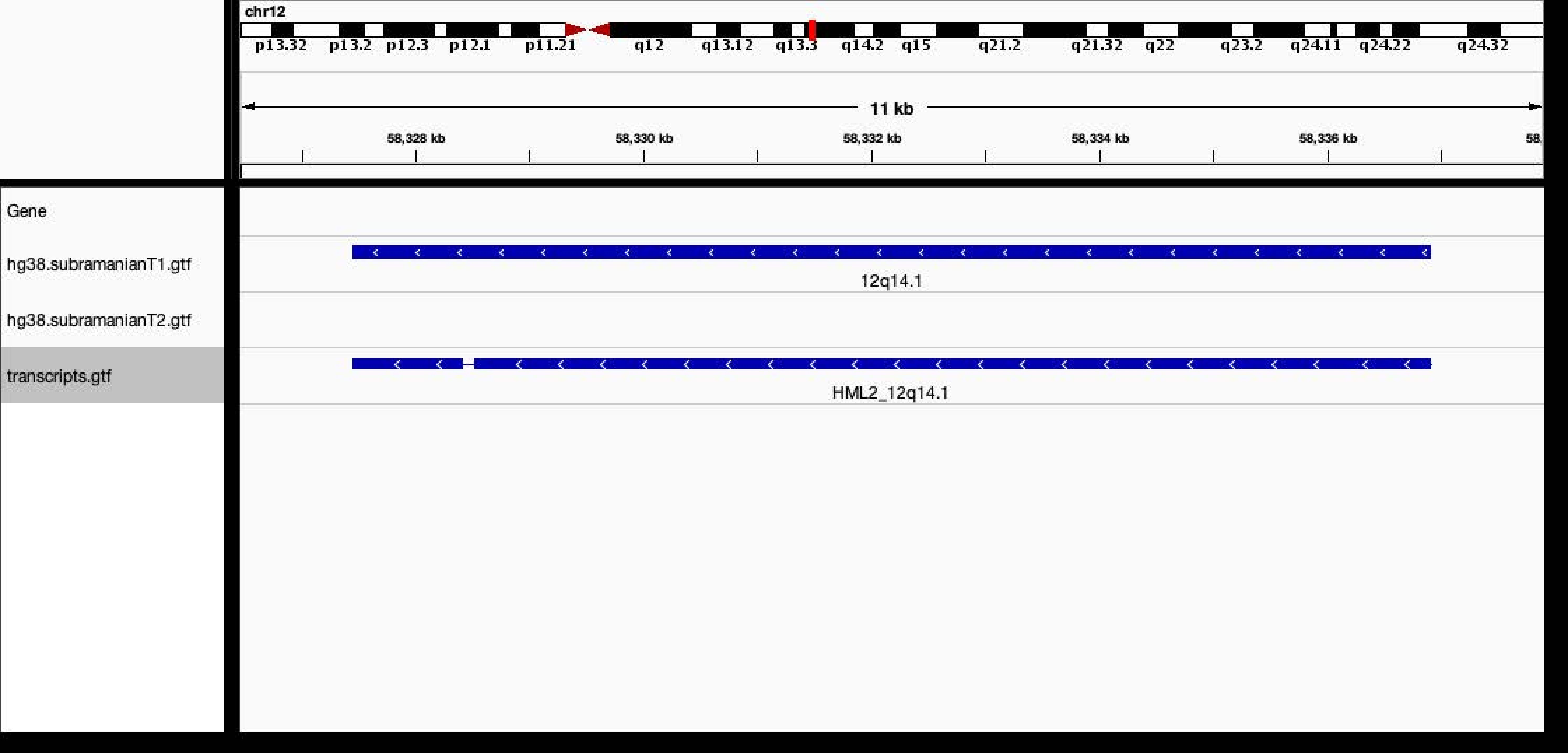
chr12
p13.32 p13.2 p12.3 p12.1 p11.21 q12 q13.12 q13.3 q14.2 q15 q21.2 q21.32 q22 q23.2 q24.11 q24.22 q24.32
11 kb
58,328 kb
58,330 kb
58,332 kb
58,334 kb
58,336 kb
Gene
hg38.subramanianT1.gtf
12q14.1
hg38.subramanianT2.gtf
transcripts.gtf
HML2_12q14.1

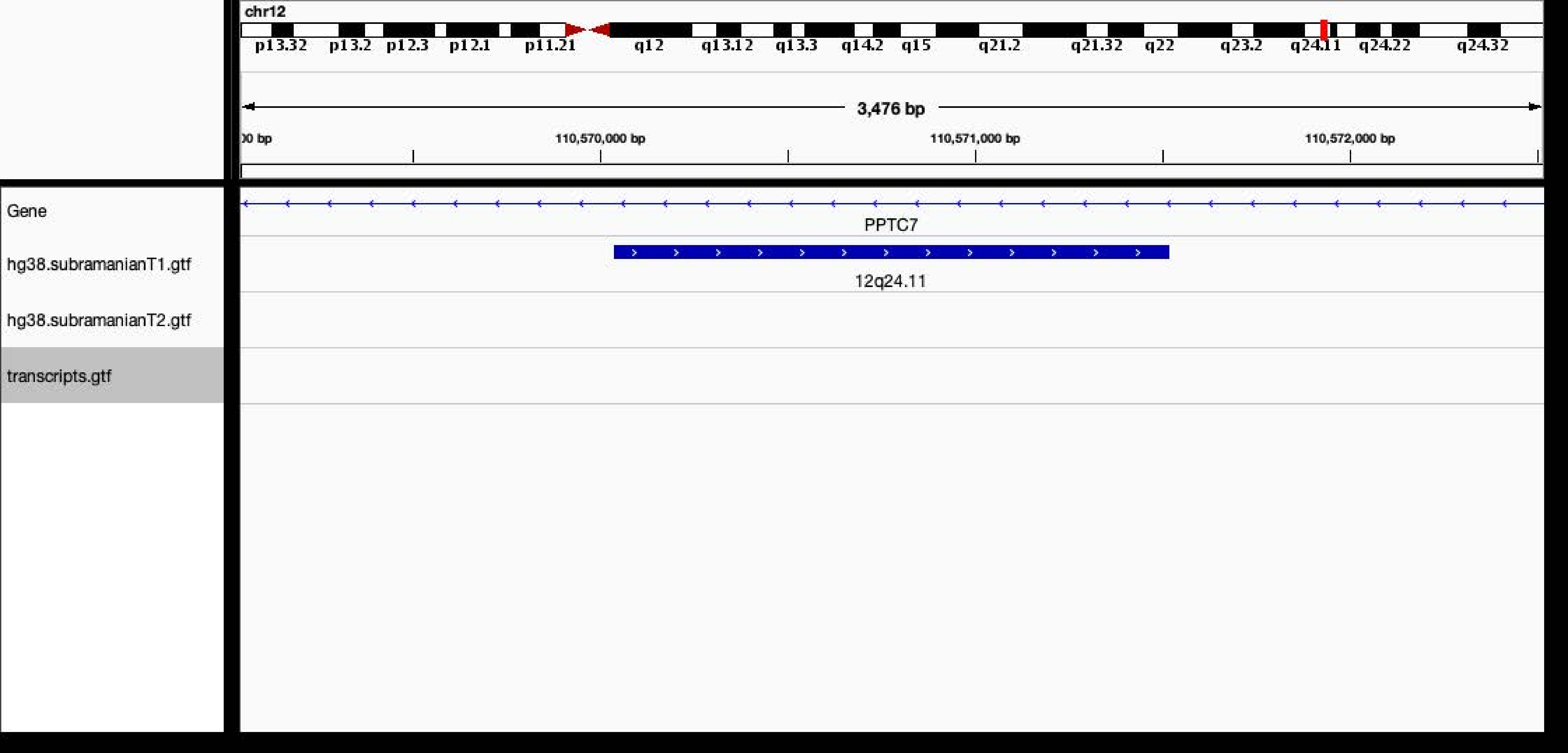
chr12
p13.32 p13.2 p12.3 p12.1 p11.21 q12 q13.12 q13.3 q14.2 q15 q21.2 q21.32 q22 q23.2 q24.11 q24.22 q24.32
3,476 bp
110,570,000 bp
110,571,000 bp
110,572,000 bp
Gene
PPTC7
hg38.subramanianT1.gtf
12q24.11
hg38.subramanianT2.gtf
transcripts.gtf

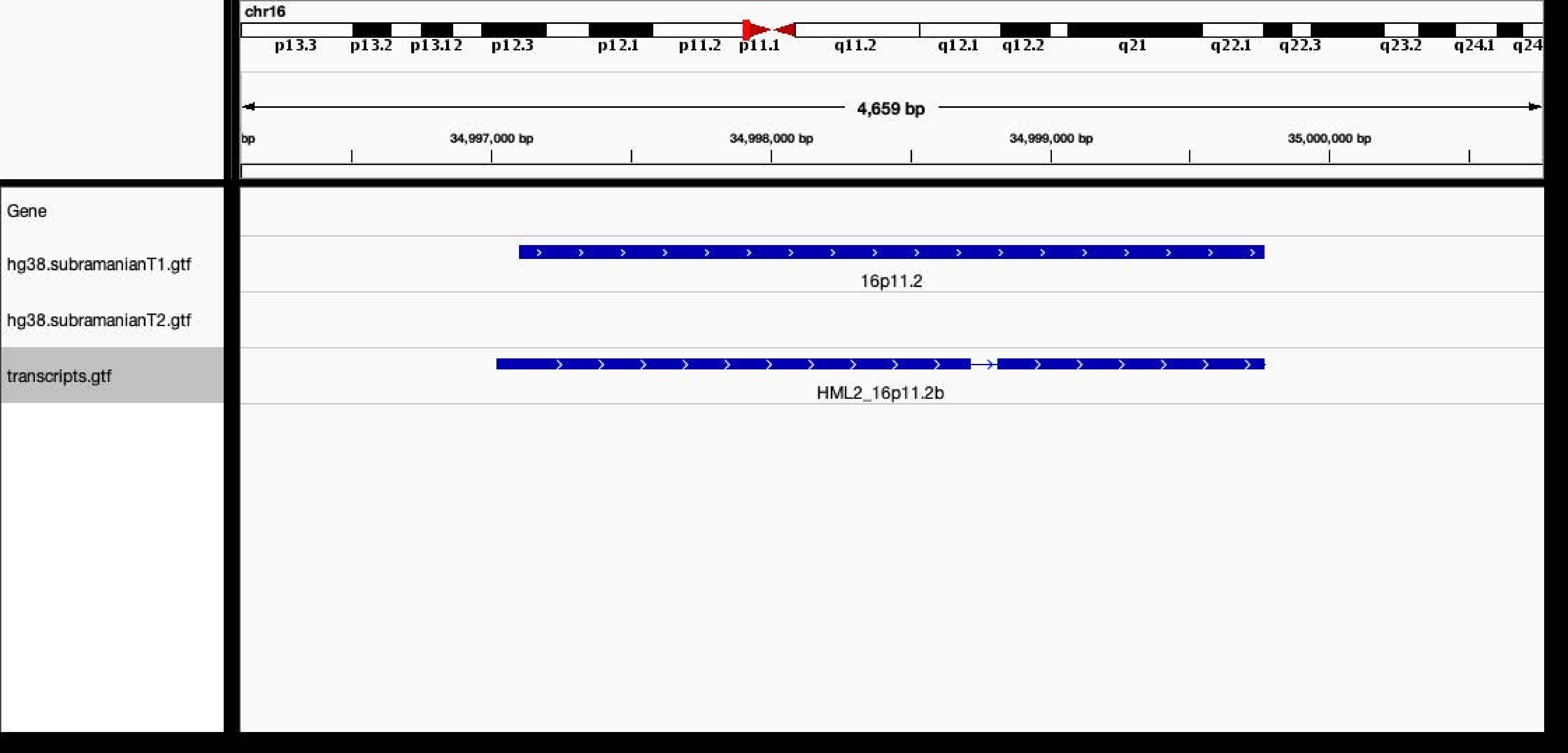

chr16
p13.3
p13.2
p13.12
p12.3
p12.1
p11.2
p11.1
q11.2
q12.1
q12.2
q21
q22.1
q22.3
q23.2
q24.1
q24
4,659 bp
34,997,000 bp
34,998,000 bp
34,999,000 bp
35,000,000 bp
Gene
hg38.subramanianT1.gtf
16p11.2
hg38.subramanianT2.gtf
transcripts.gtf
HML2_16p11.2b

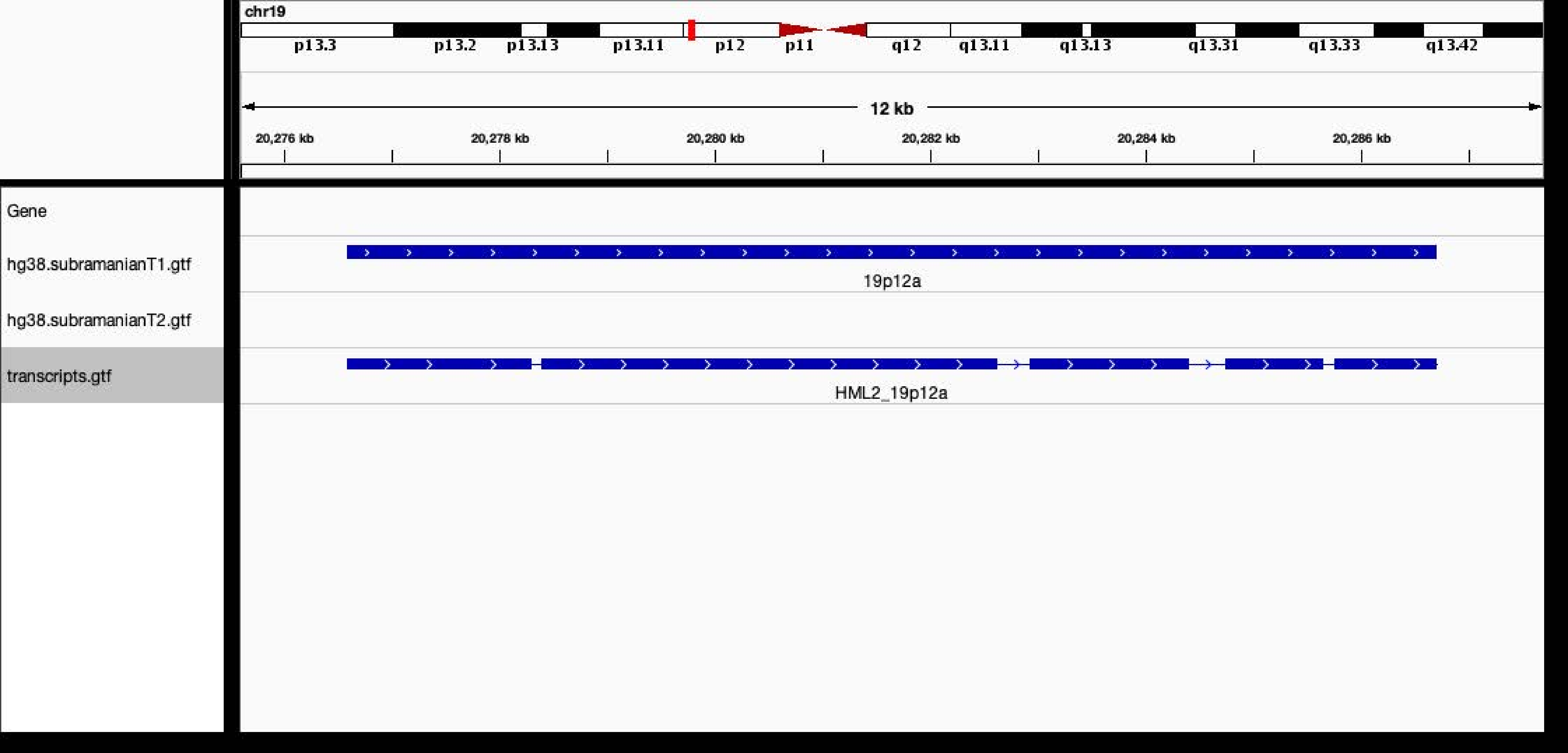

chr19
p13.3
p13.2
p13.13
p13.11
p12
p11
q12
q13.11
q13.13
q13.31
q13.33
q13.42
12 kb
20,276 kb
20,278 kb
20,280 kb
20,282 kb
20,284 kb
20,286 kb
Gene
hg38.subramanianT1.gtf
19p12a
hg38.subramanianT2.gtf
transcripts.gtf
HML2_19p12a

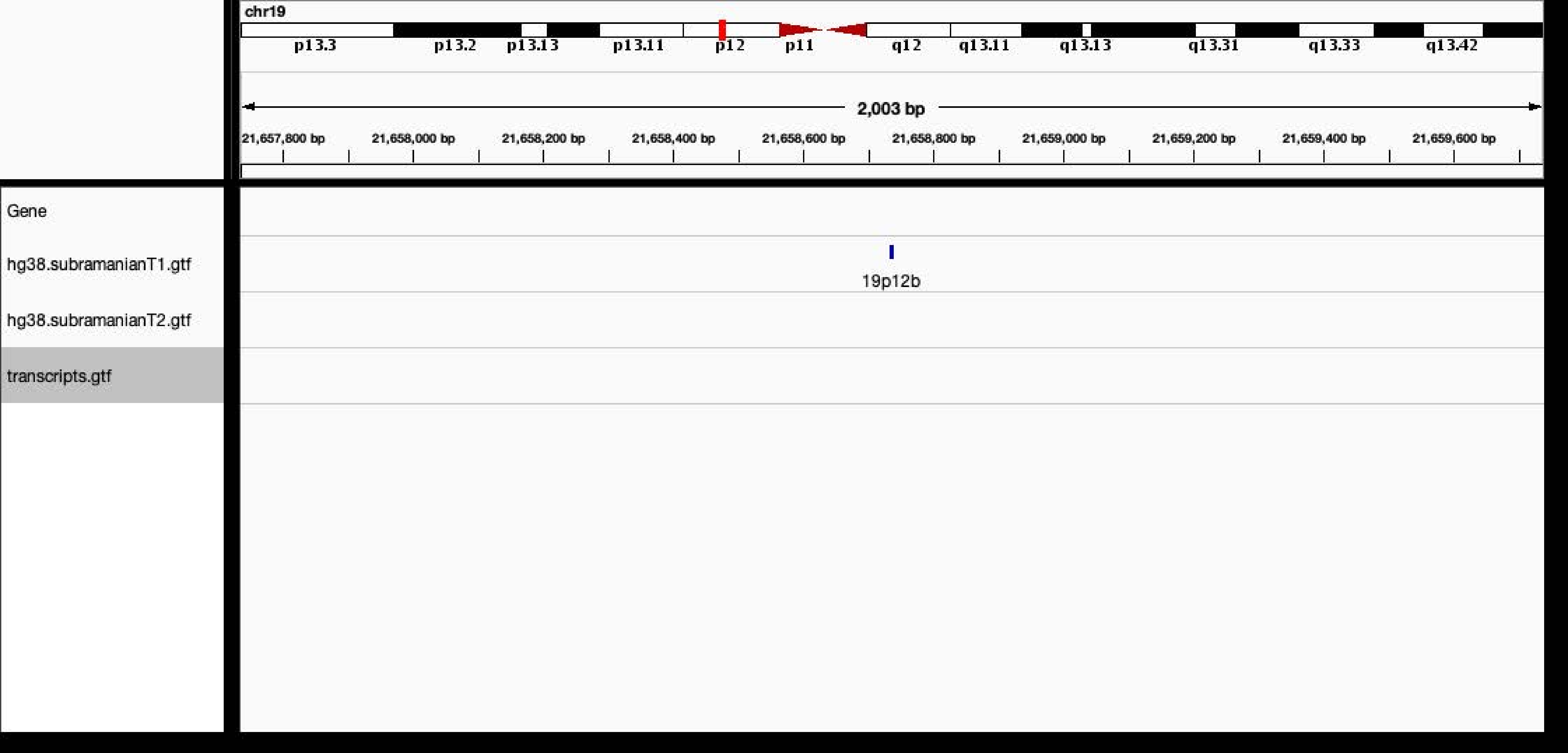
chr19
p13.3
p13.2
p13.13
p13.11
p12
p11
q12
q13.11
q13.13
q13.31
q13.33
q13.42
2,003 bp
21,657,800 bp
21,658,000 bp
21,658,200 bp
21,658,400 bp
21,658,600 bp
21,658,800 bp
21,659,000 bp
21,659,200 bp
21,659,400 bp
21,659,600 bp
Gene
hg38.subramanianT1.gtf
19p12b
hg38.subramanianT2.gtf
transcripts.gtf

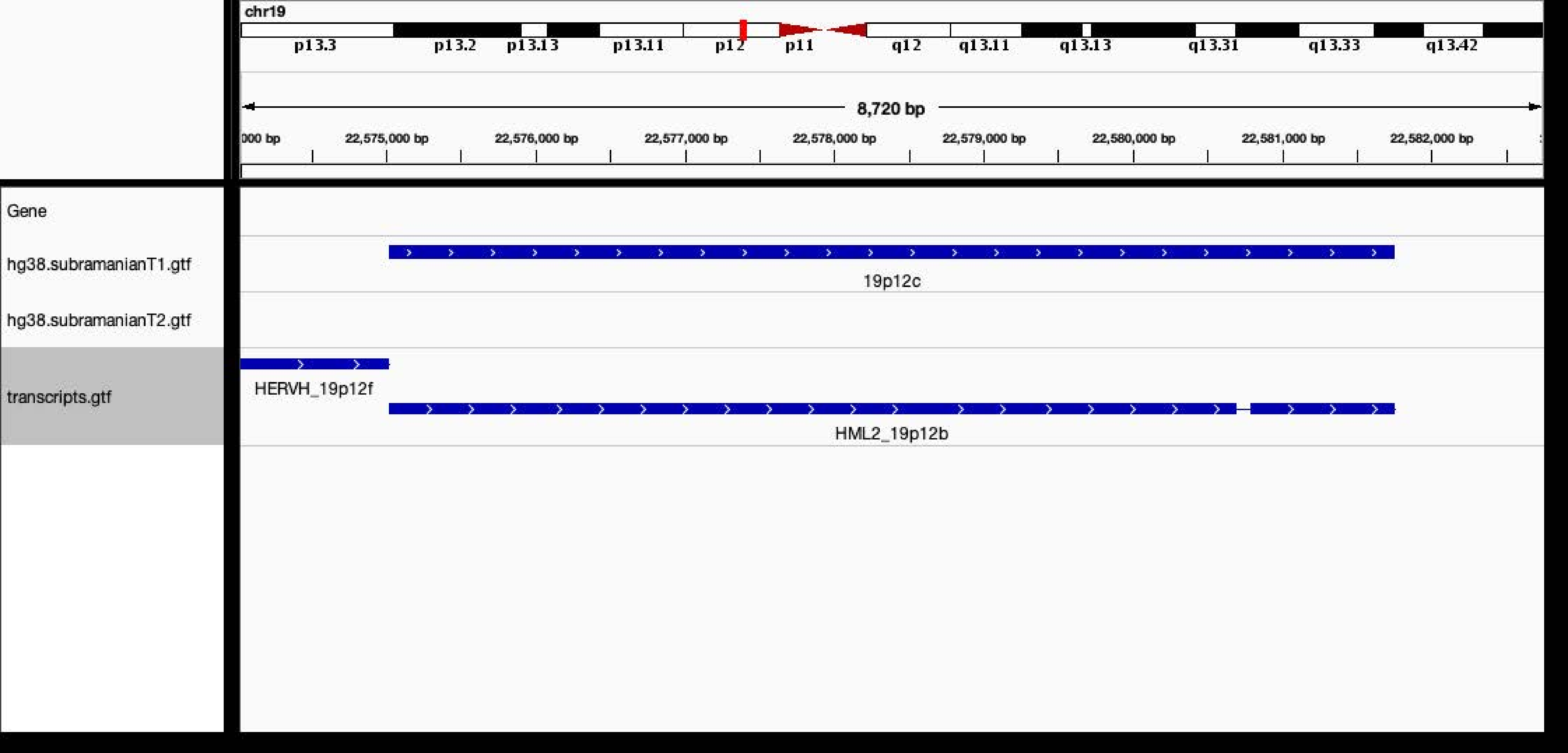
chr19
p13.3
p13.2
p13.13
p13.11
p12
p11
q12
q13.11
q13.13
q13.31
q13.33
q13.42
8,720 bp
22,575,000 bp
22,576,000 bp
22,577,000 bp
22,578,000 bp
22,579,000 bp
22,580,000 bp
22,581,000 bp
22,582,000 bp
Gene
hg38.subramanianT1.gtf
19p12c
hg38.subramanianT2.gtf
transcripts.gtf
HERVH_19p12f
HML2_19p12b

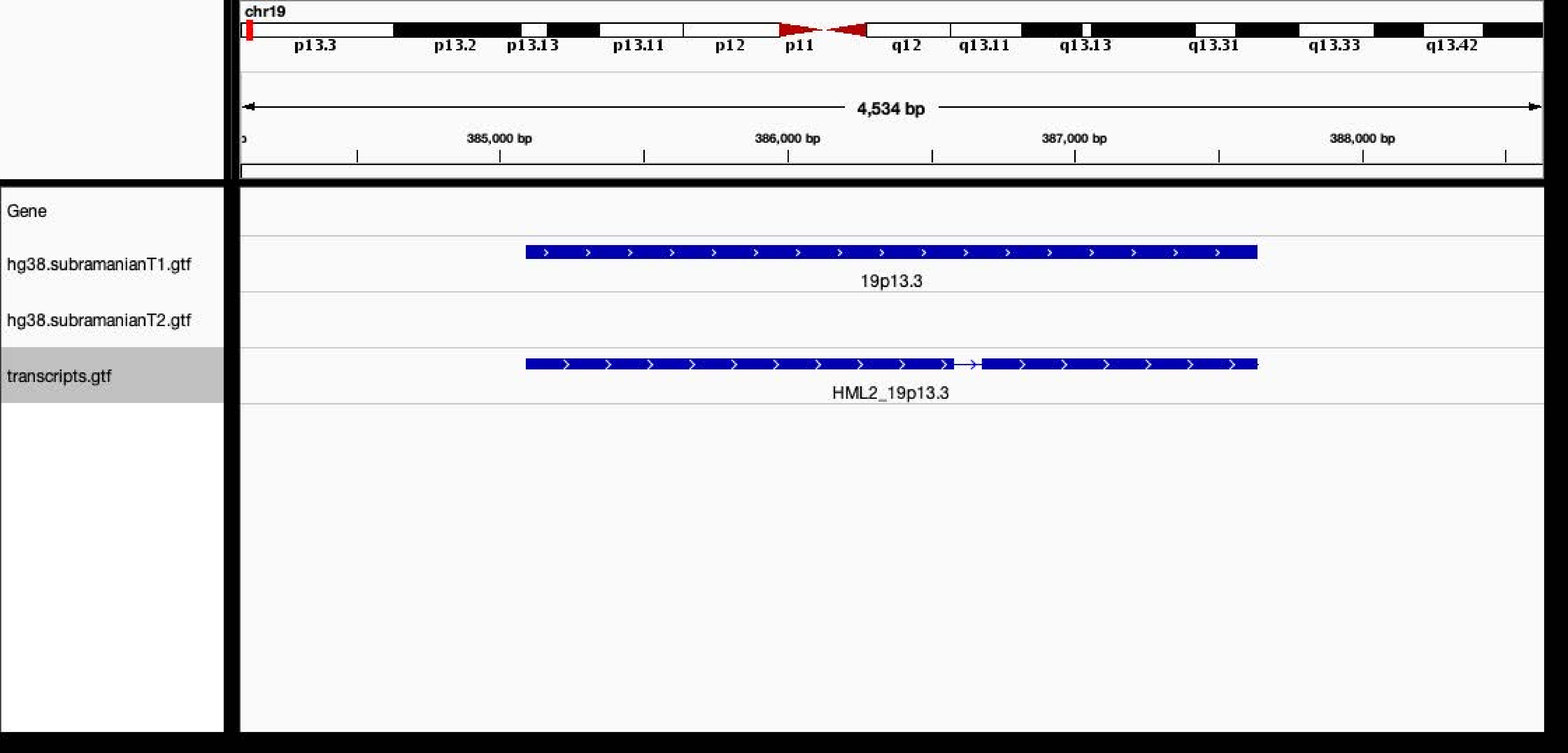

chr19
p13.3
p13.2
p13.13
p13.11
p12
p11
q12
q13.11
q13.13
q13.31
q13.33
q13.42
4,534 bp
385,000 bp
386,000 bp
387,000 bp
388,000 bp
Gene
hg38.subramanianT1.gtf
19p13.3
hg38.subramanianT2.gtf
transcripts.gtf
HML2_19p13.3

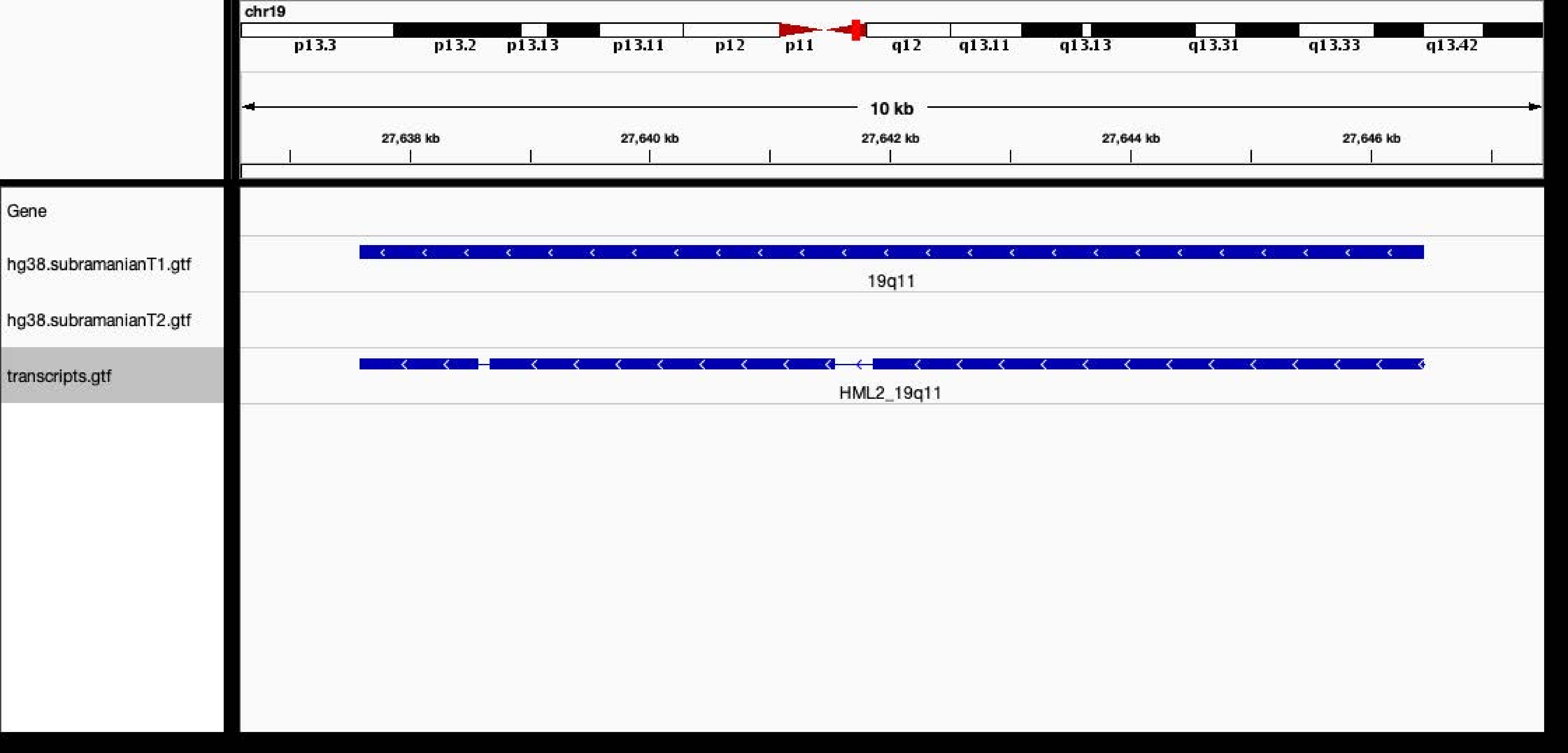
chr19
p13.3
p13.2
p13.13
p13.11
p12
p11
q12
q13.11
q13.13
q13.31
q13.33
q13.42
10 kb
27,638 kb
27,640 kb
27,642 kb
27,644 kb
27,646 kb
Gene
hg38.subramanianT1.gtf
19q11
hg38.subramanianT2.gtf
transcripts.gtf
HML2_19q11

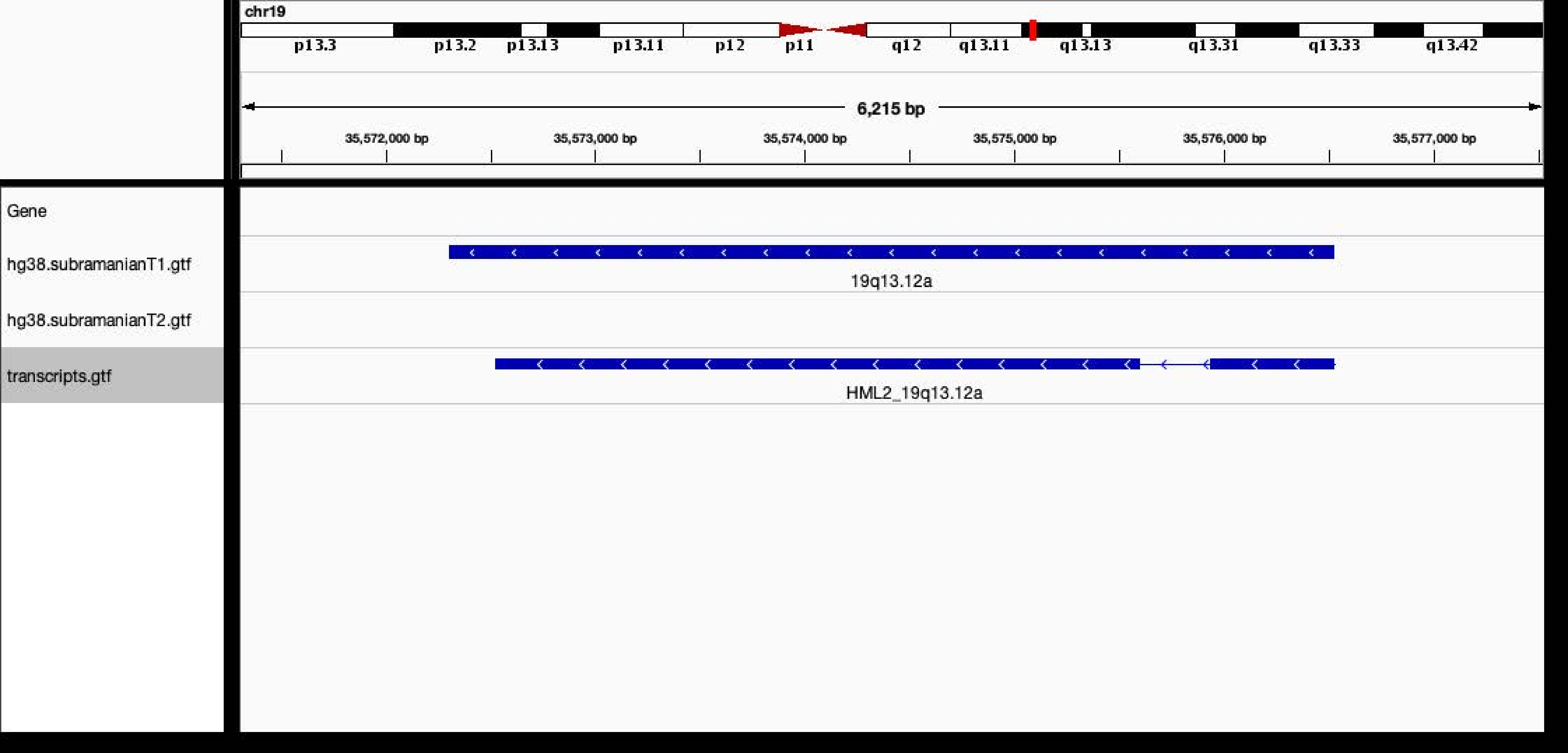
chr19
p13.3
p13.2
p13.13
p13.11
p12
p11
q12
q13.11
q13.13
q13.31
q13.33
q13.42
6,215 bp
35,572,000 bp
35,573,000 bp
35,574,000 bp
35,575,000 bp
35,576,000 bp
35,577,000 bp
Gene
hg38.subramanianT1.gtf
hg38.subramanianT2.gtf
transcripts.gtf
19q13.12a
HML2_19q13.12a

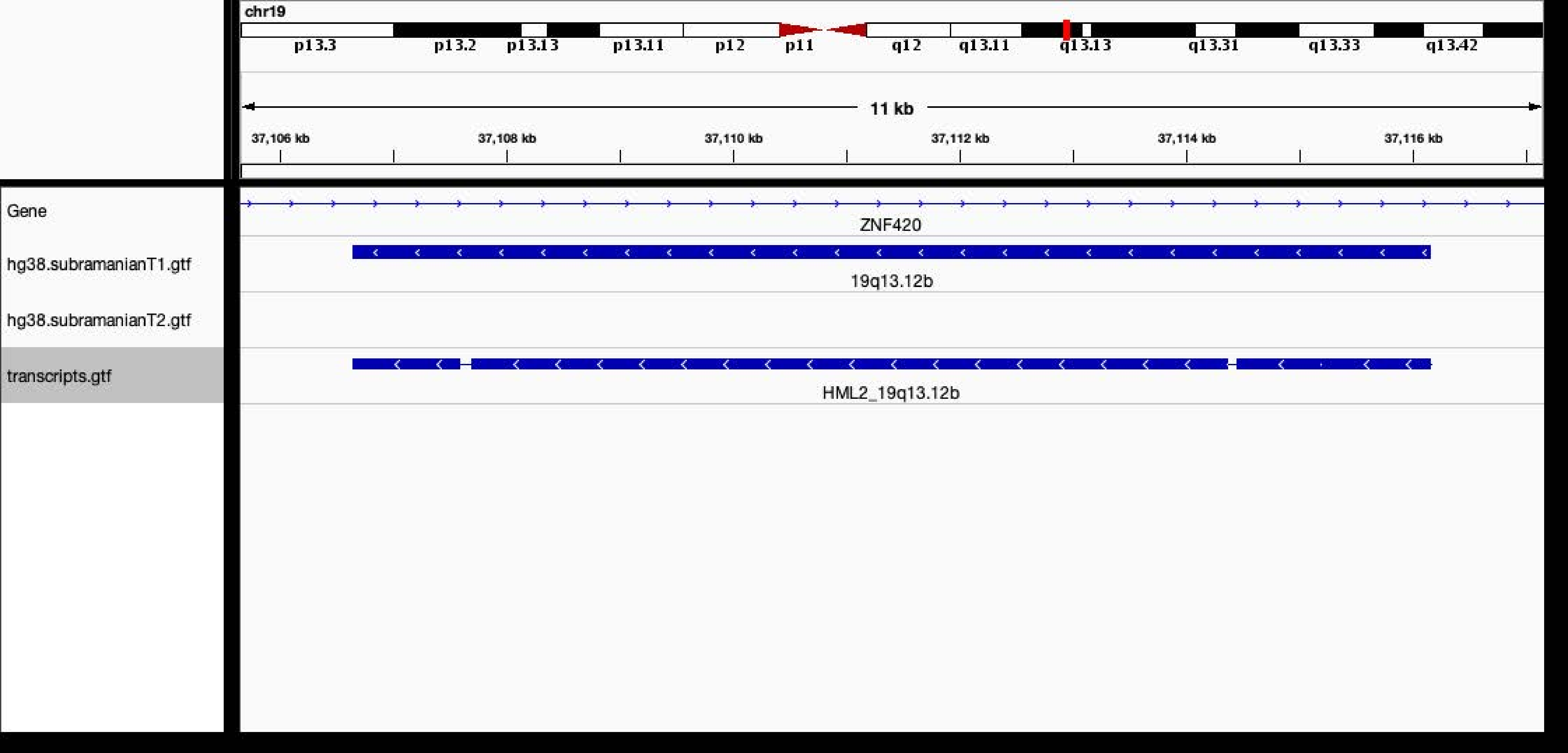
chr19
p13.3
p13.2
p13.13
p13.11
p12
p11
q12
q13.11
q13.13
q13.31
q13.33
q13.42
11 kb
37,106 kb
37,108 kb
37,110 kb
37,112 kb
37,114 kb
37,116 kb
Gene
ZNF420
hg38.subramanianT1.gtf
19q13.12b
hg38.subramanianT2.gtf
transcripts.gtf
HML2_19q13.12b

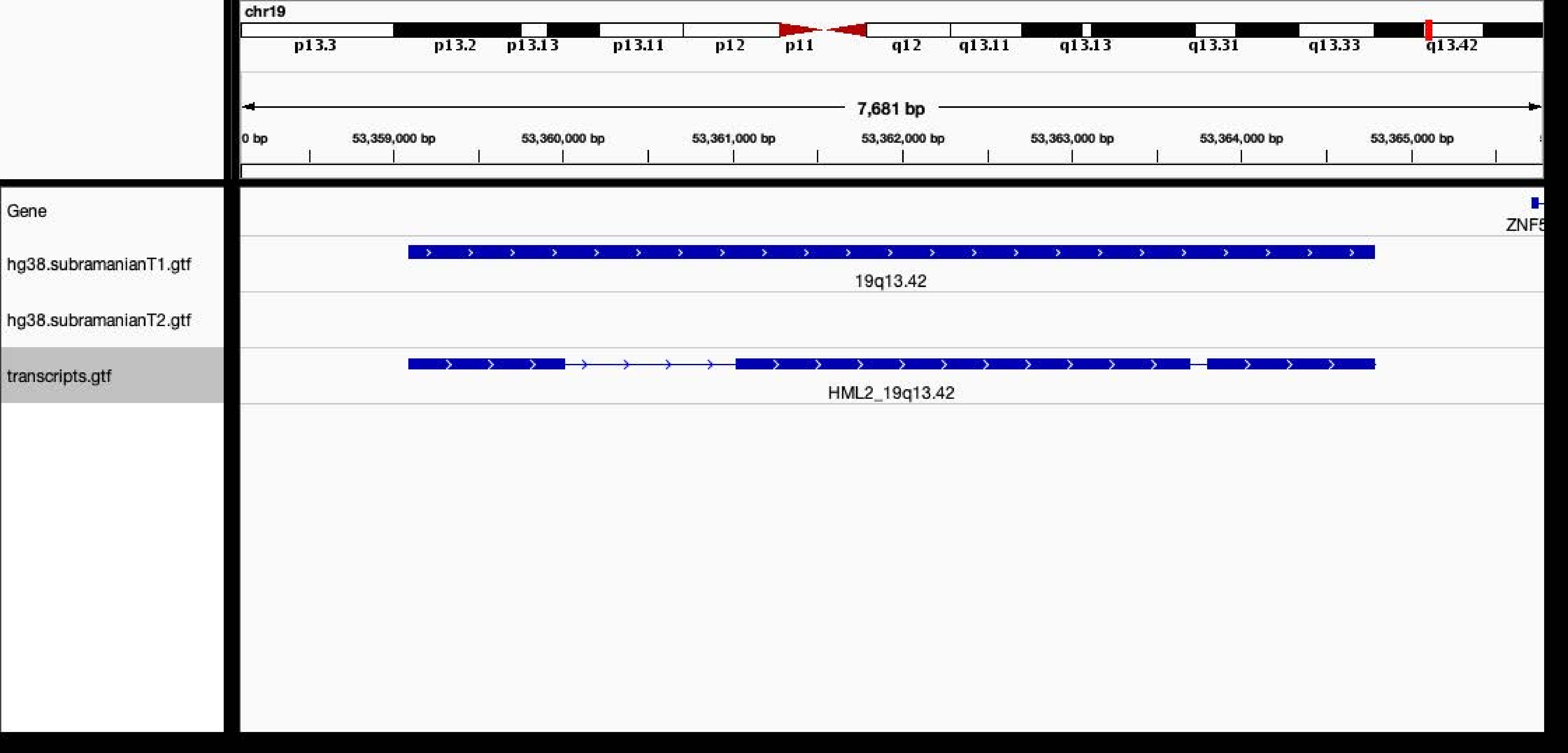
chr19
p13.3
p13.2
p13.13
p13.11
p12
p11
q12
q13.11
q13.13
q13.31
q13.33
q13.42
7,681 bp
0 bp
53,359,000 bp
53,360,000 bp
53,361,000 bp
53,362,000 bp
53,363,000 bp
53,364,000 bp
53,365,000 bp
Gene
hg38.subramanianT1.gtf
hg38.subramanianT2.gtf
transcripts.gtf
19q13.42
HML2_19q13.42

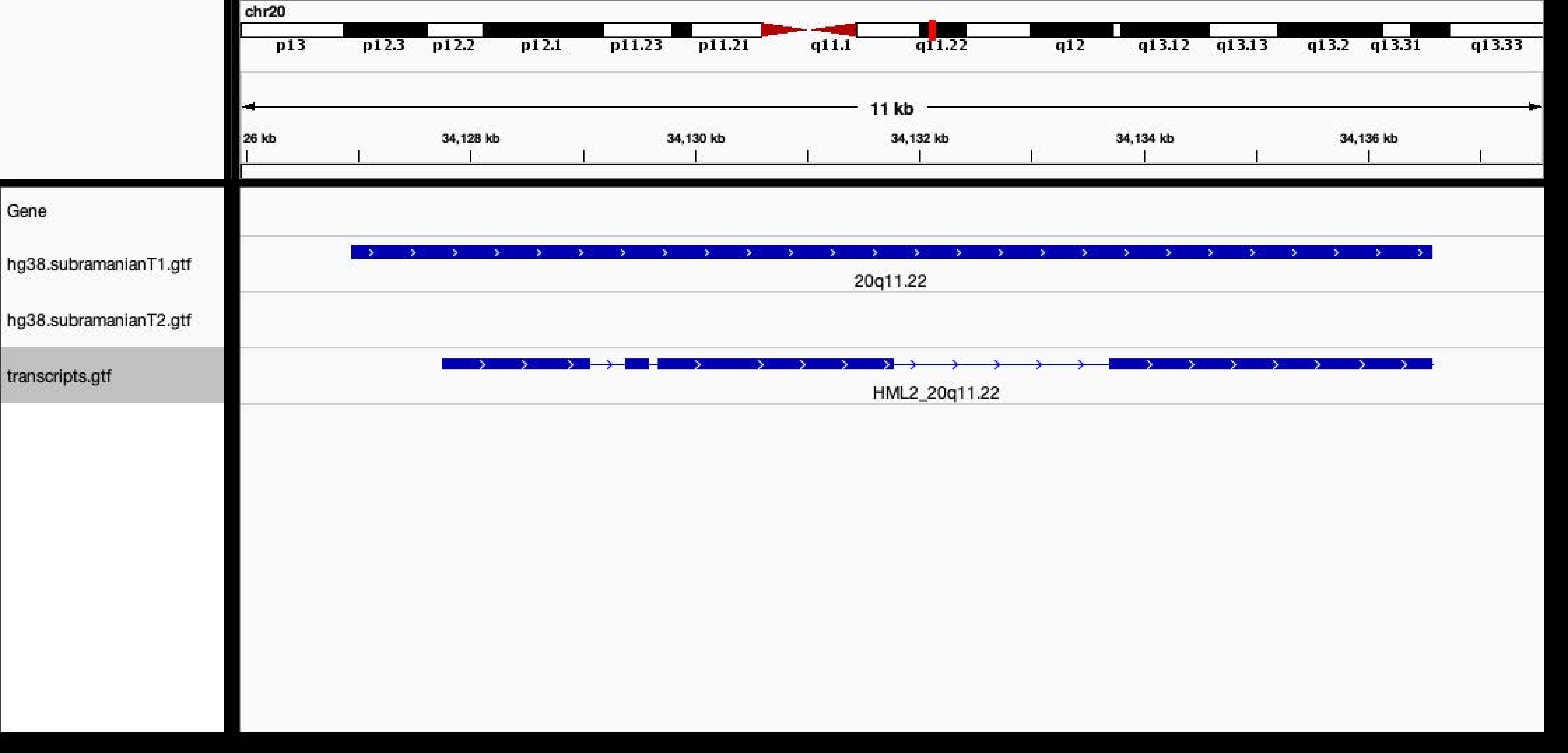
chr20
p13
p12.3
p12.2
p12.1
p11.23
p11.21
q11.1
q11.22
q12
q13.12
q13.13
q13.2
q13.31
q13.33
11 kb
26 kb
34,128 kb
34,130 kb
34,132 kb
34,134 kb
34,136 kb
Gene
hg38.subramanianT1.gtf
20q11.22
hg38.subramanianT2.gtf
transcripts.gtf
HML2_20q11.22

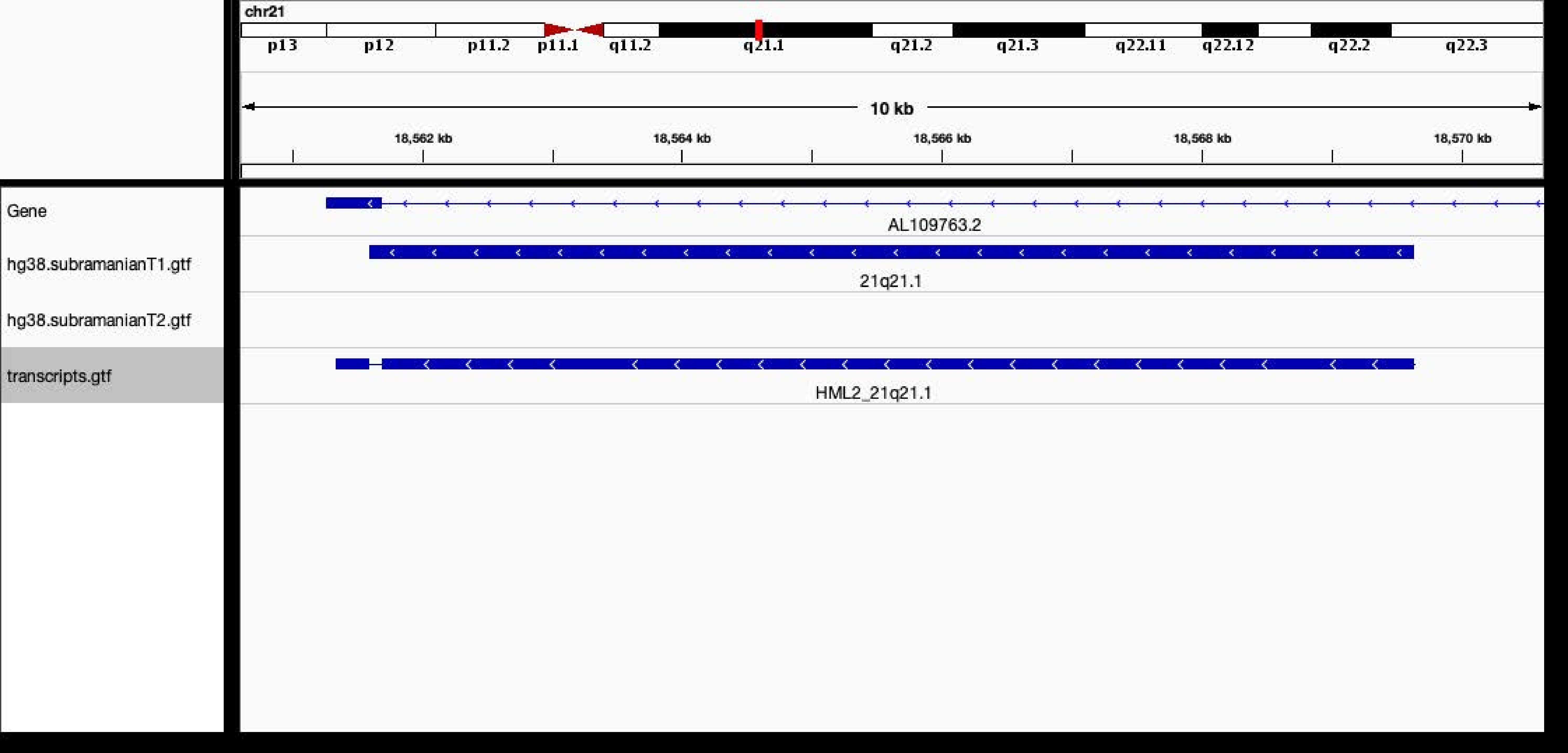

chr21
p13
p12
p11.2
p11.1
q11.2
q21.1
q21.2
q21.3
q22.11
q22.12
q22.2
q22.3
10 kb
18,562 kb
18,564 kb
18,566 kb
18,568 kb
18,570 kb
Gene
AL109763.2
hg38.subramanianT1.gtf
21q21.1
hg38.subramanianT2.gtf
transcripts.gtf
HML2_21q21.1

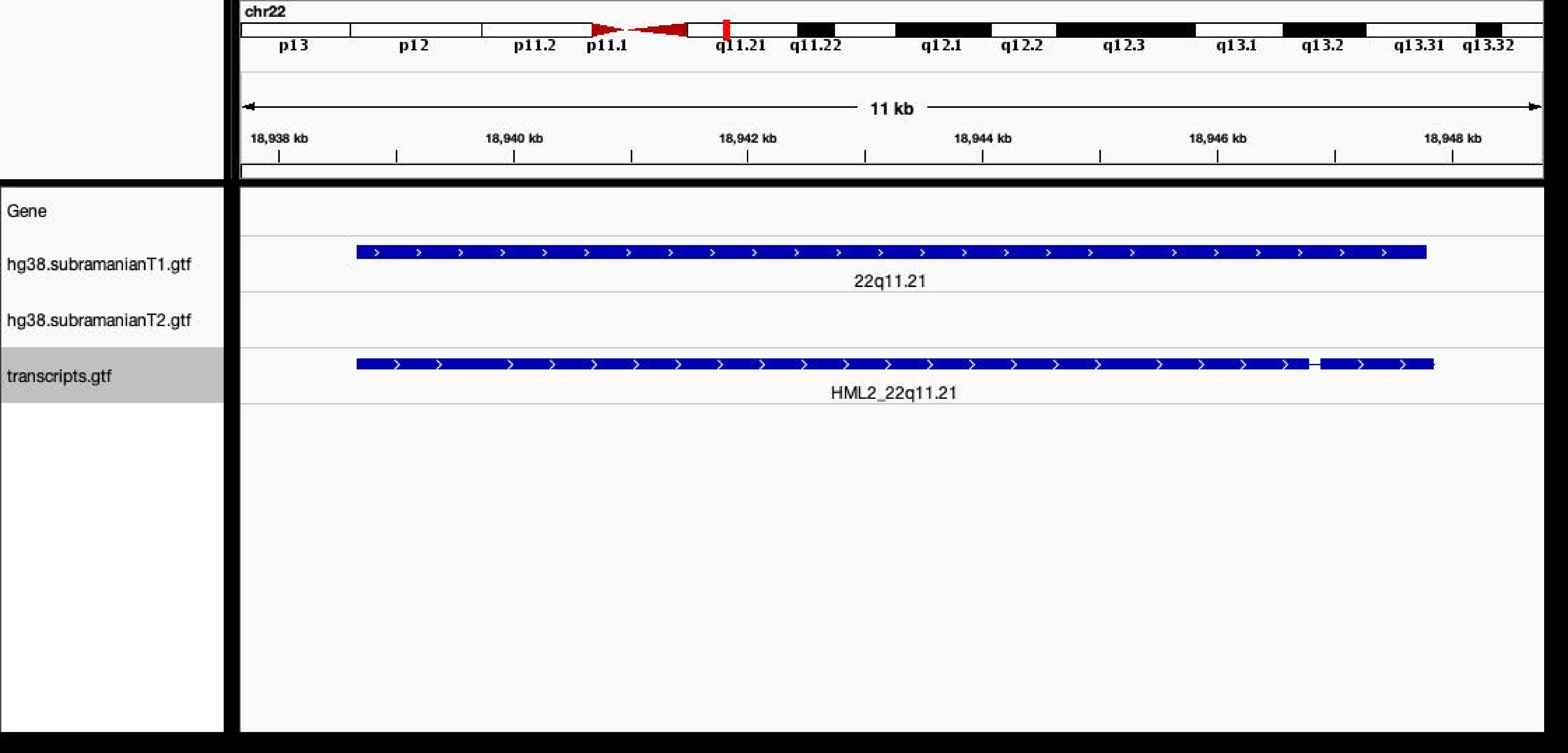
chr22
p13
p12
p11.2
p11.1
q11.21
q11.22
q12.1
q12.2
q12.3
q13.1
q13.2
q13.31
q13.32
11 kb
18,938 kb
18,940 kb
18,942 kb
18,944 kb
18,946 kb
18,948 kb
Gene
hg38.subramanianT1.gtf
22q11.21
hg38.subramanianT2.gtf
transcripts.gtf
HML2_22q11.21

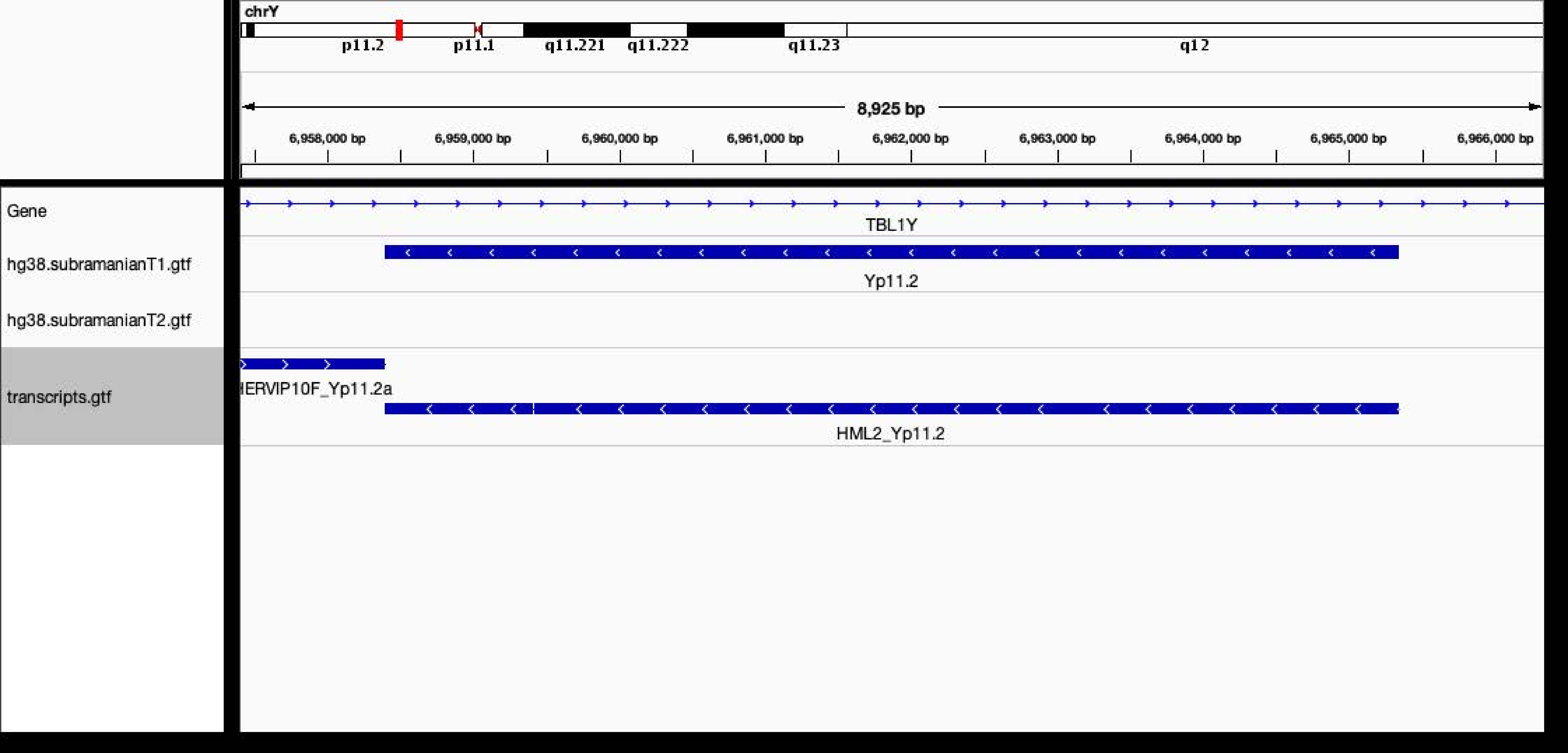

chrY
p11.2
p11.1
q11.221
q11.222
q11.23
q12
8,925 bp
6,958,000 bp
6,959,000 bp
6,960,000 bp
6,961,000 bp
6,962,000 bp
6,963,000 bp
6,964,000 bp
6,965,000 bp
6,966,000 bp
Gene
TBL1Y
hg38.subramanianT1.gtf
Yp11.2
hg38.subramanianT2.gtf
transcripts.gtf
HERVIP10F_Yp11.2a
HML2_Yp11.2

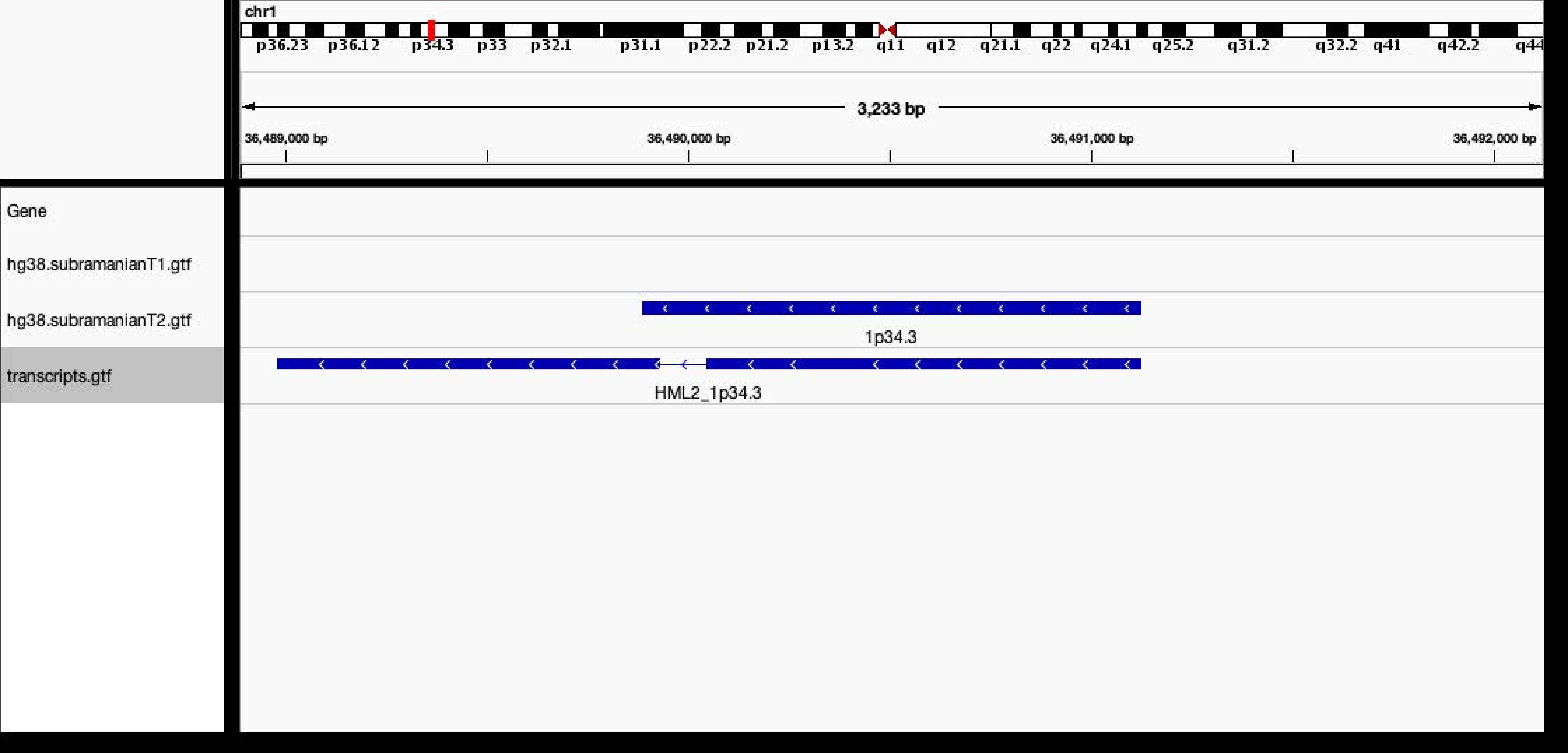
chr1
p36.23
p36.12
p34.3
p33
p32.1
p31.1
p22.2
p21.2
p13.2
q11
q12
q21.1
q22
q24.1
q25.2
q31.2
q32.2
q41
q42.2
3,233 bp
36,489,000 bp
36,490,000 bp
36,491,000 bp
36,492,000 bp
Gene
hg38.subramanianT1.gtf
hg38.subramanianT2.gtf
transcripts.gtf
1p34.3
HML2_1p34.3

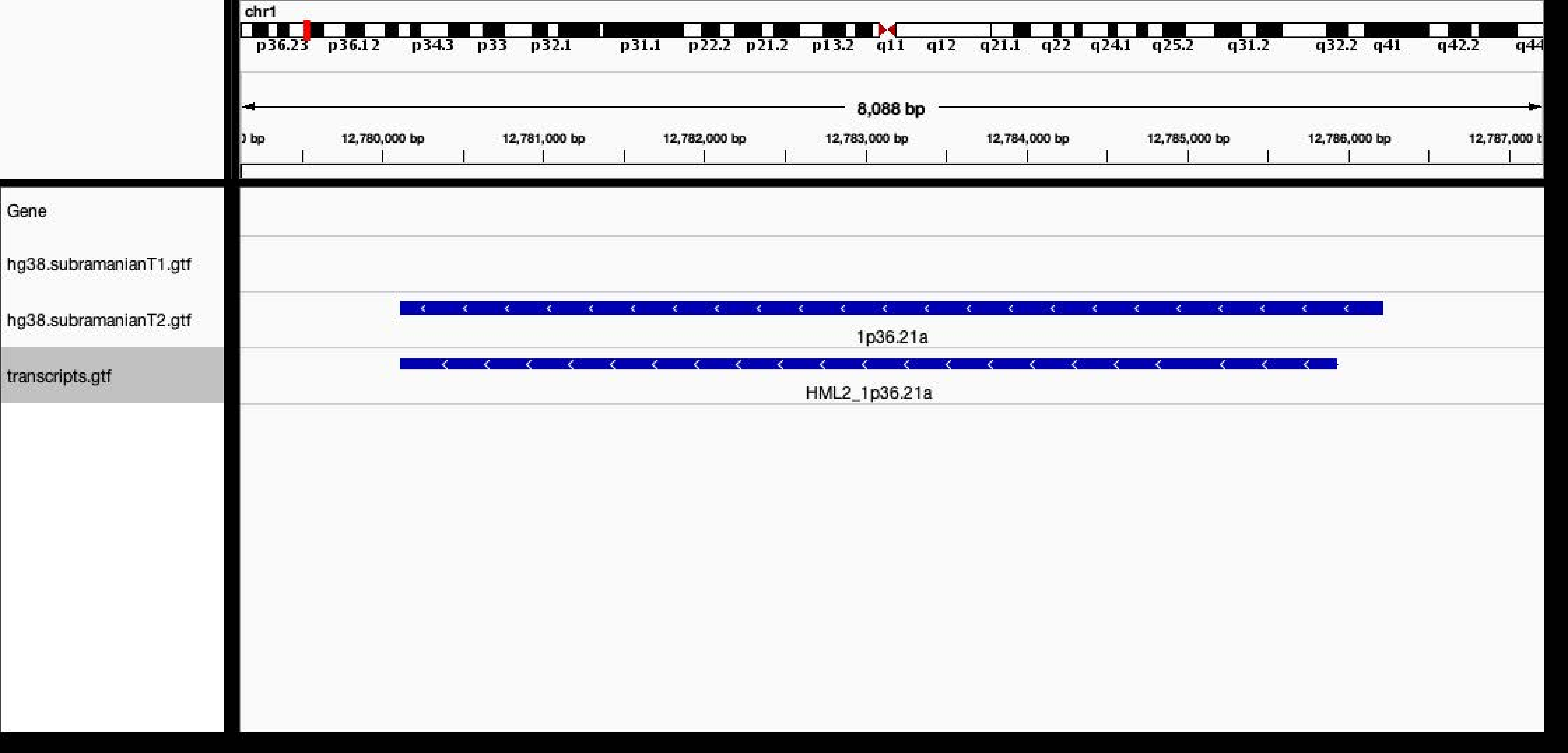
chr1
p36.23
p36.12
p34.3
p33
p32.1
p31.1
p22.2
p21.2
p13.2
q11
q12
q21.1
q22
q24.1
q25.2
q31.2
q32.2
q41
q42.2
8,088 bp
12,780,000 bp
12,781,000 bp
12,782,000 bp
12,783,000 bp
12,784,000 bp
12,785,000 bp
12,786,000 bp
Gene
hg38.subramanianT1.gtf
hg38.subramanianT2.gtf
transcripts.gtf
1p36.21a
HML2_1p36.21a

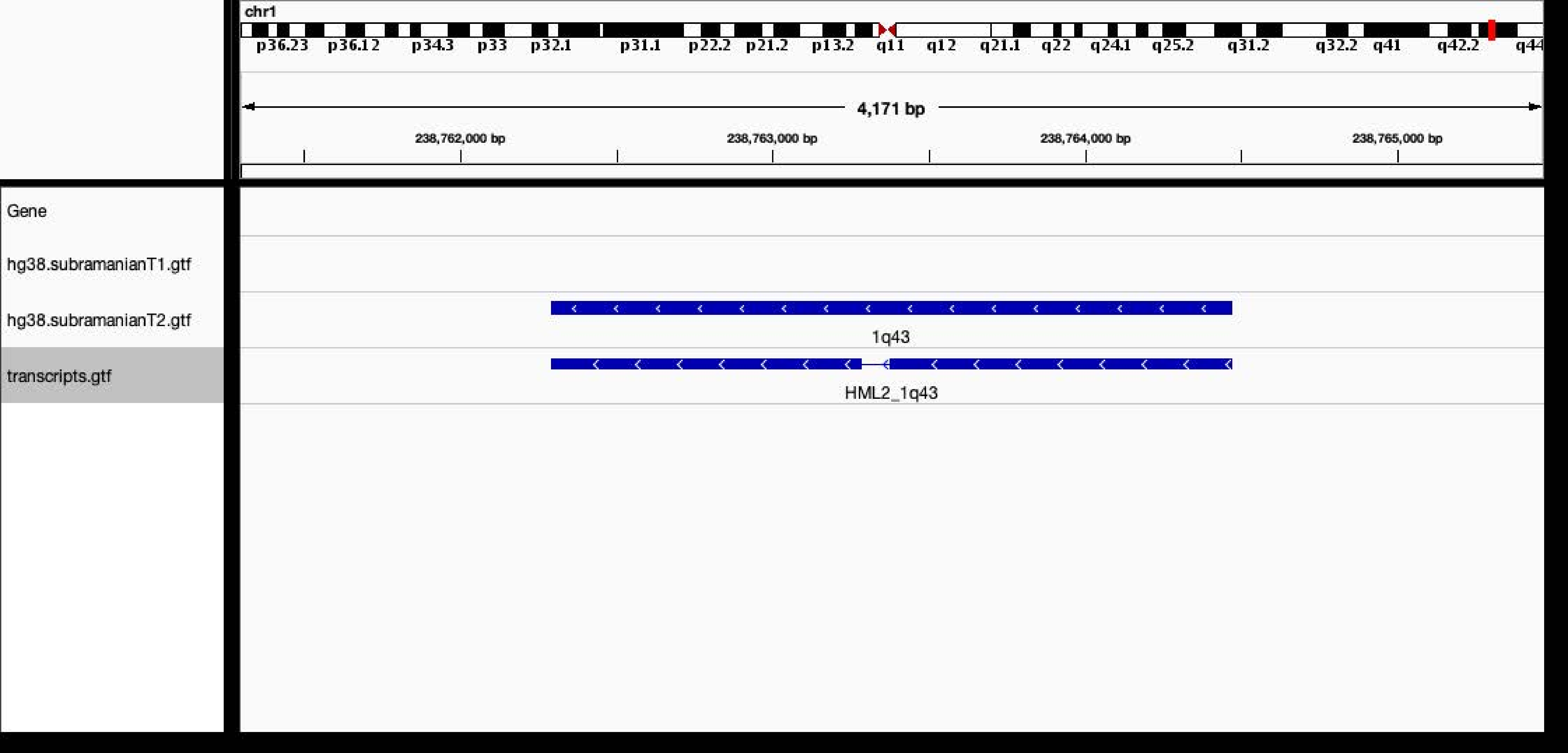

chr1
p36.23
p36.12
p34.3
p33
p32.1
p31.1
p22.2
p21.2
p13.2
q11
q12
q21.1
q22
q24.1
q25.2
q31.2
q32.2
q41
q42.2
4,171 bp
238,762,000 bp
238,763,000 bp
238,764,000 bp
238,765,000 bp
Gene
hg38.subramanianT1.gtf
hg38.subramanianT2.gtf
transcripts.gtf
1q43
HML2_1q43

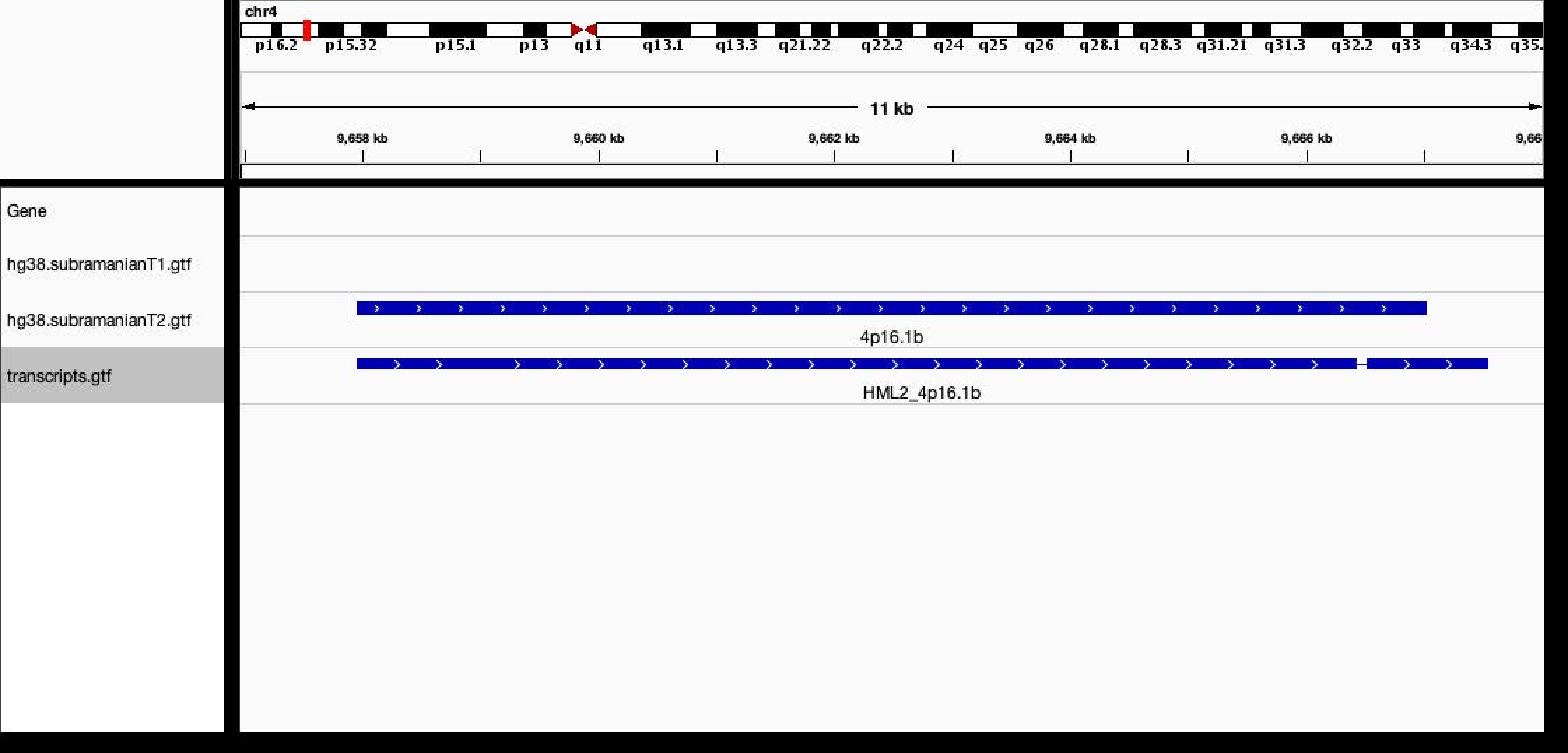

chr4
p16.2
p15.32
p15.1
p13
q11
q13.1
q13.3
q21.22
q22.2
q24
q25
q26
q28.1
q28.3
q31.21
q31.3
q32.2
q33
q34.3
11 kb
9,658 kb
9,660 kb
9,662 kb
9,664 kb
9,666 kb
Gene
hg38.subramanianT1.gtf
hg38.subramanianT2.gtf
transcripts.gtf
4p16.1b
HML2_4p16.1b

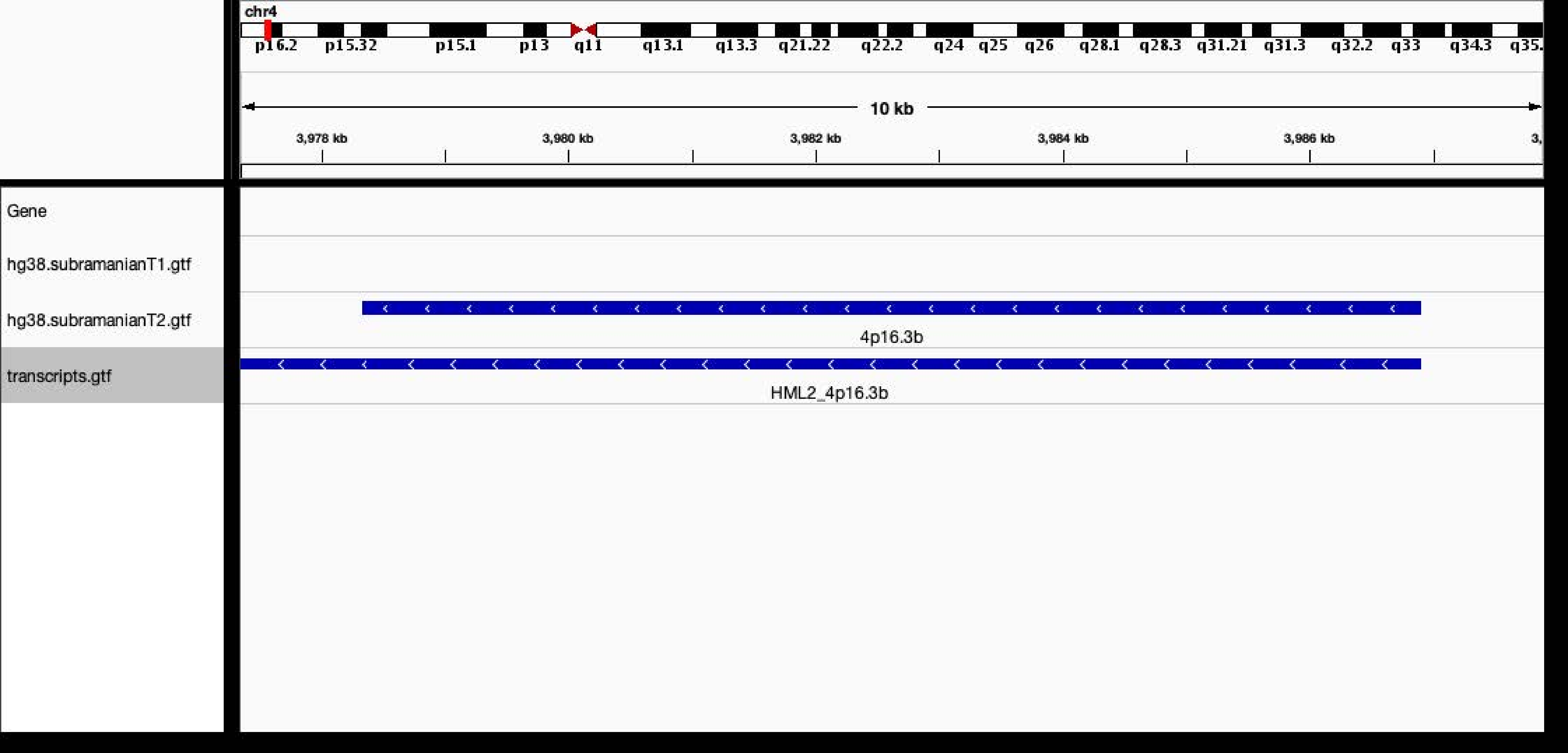
chr4
p16.2
p15.32
p15.1
p13
q11
q13.1
q13.3
q21.22
q22.2
q24
q25
q26
q28.1
q28.3
q31.21
q31.3
q32.2
q33
q34.3
10 kb
3,978 kb
3,980 kb
3,982 kb
3,984 kb
3,986 kb
Gene
hg38.subramanianT1.gtf
hg38.subramanianT2.gtf
transcripts.gtf
4p16.3b
HML2_4p16.3b

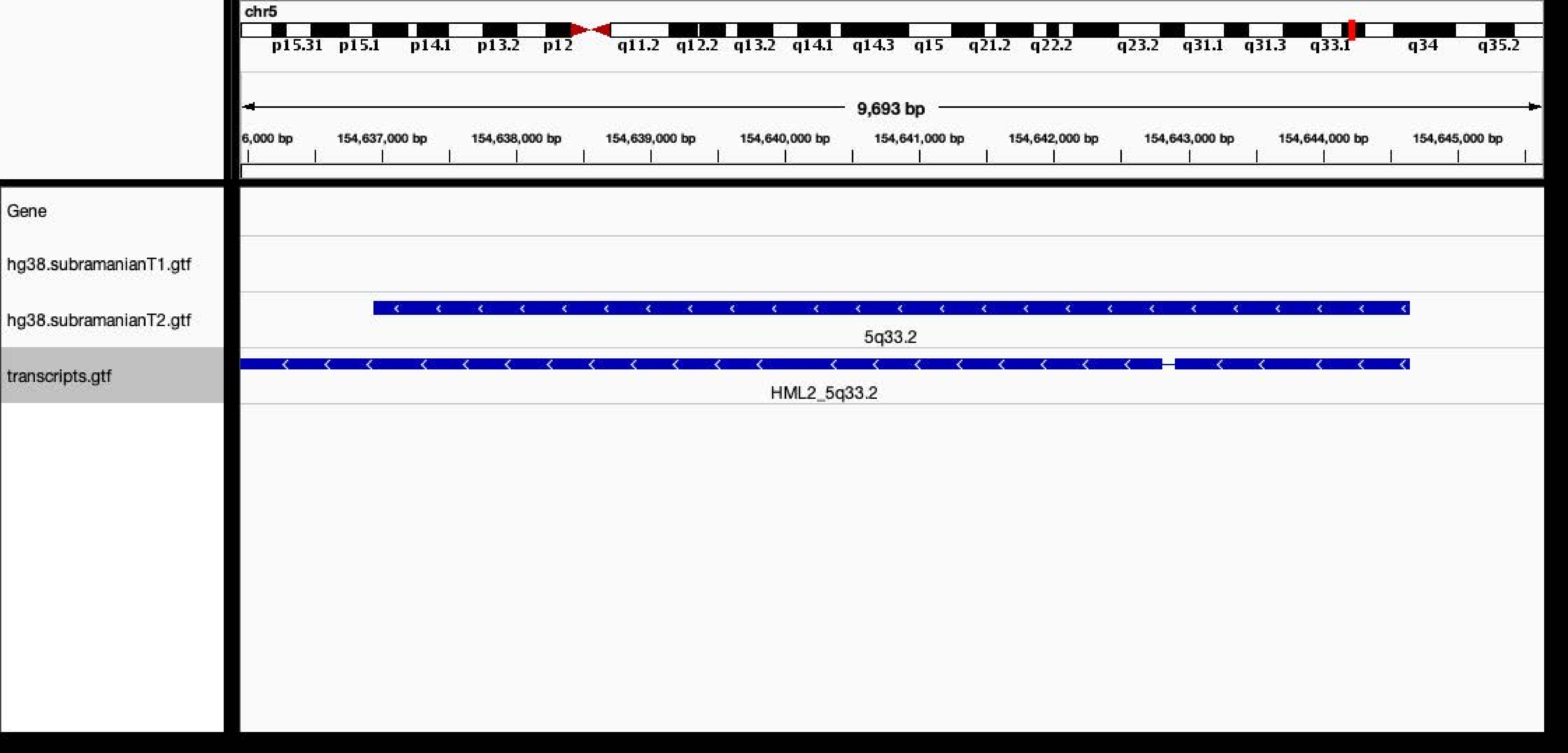
chr5
p15.31 p15.1 p14.1 p13.2 p12 q11.2 q12.2 q13.2 q14.1 q14.3 q15 q21.2 q22.2 q23.2 q31.1 q31.3 q33.1 q34 q35.2
9,693 bp
6,000 bp 154,637,000 bp 154,638,000 bp 154,639,000 bp 154,640,000 bp 154,641,000 bp 154,642,000 bp 154,643,000 bp 154,644,000 bp 154,645,000 bp
Gene
hg38.subramanianT1.gtf
hg38.subramanianT2.gtf
5q33.2
transcripts.gtf
HML2_5q33.2

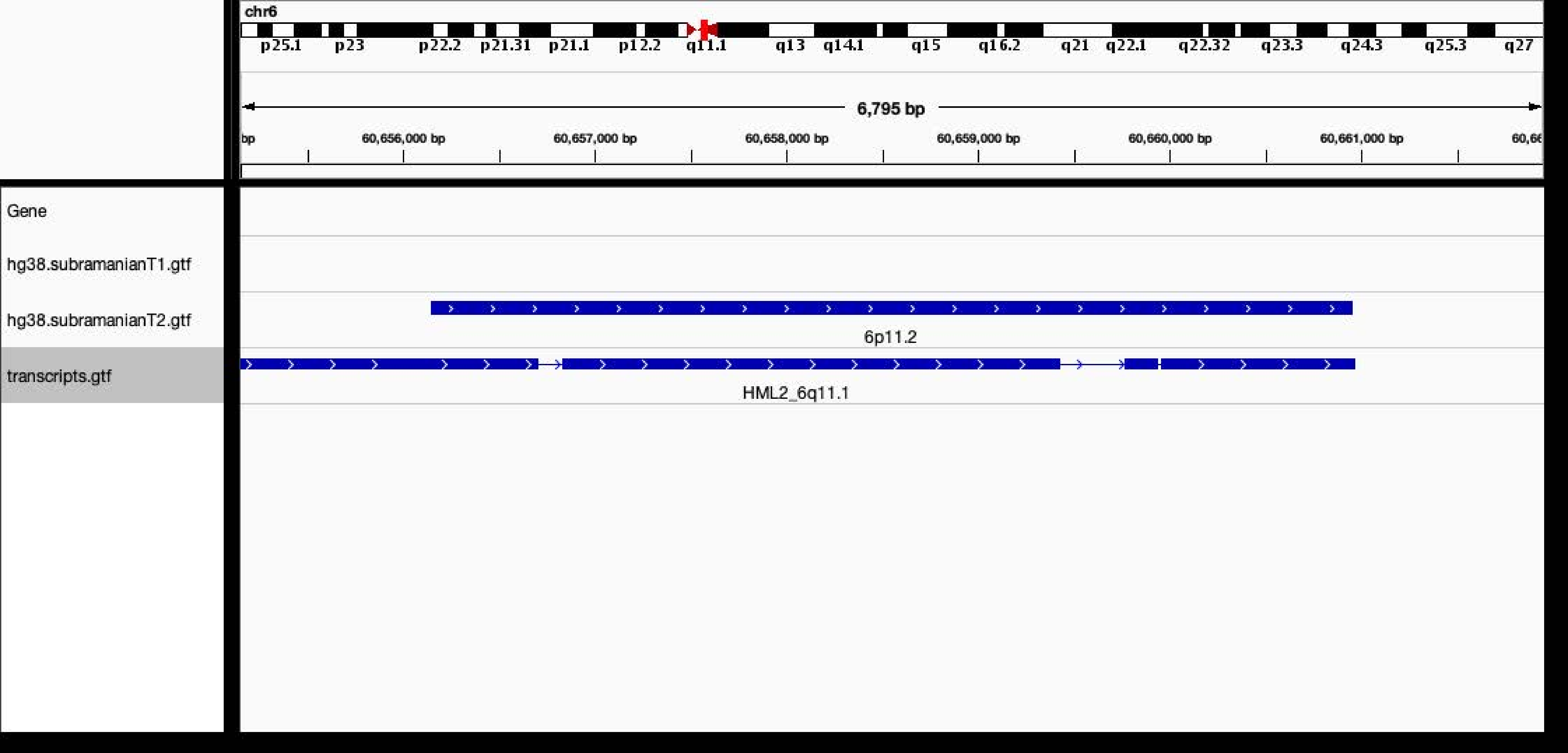

chr6
p25.1
p23
p22.2
p21.31
p21.1
p12.2
q11.1
q13
q14.1
q15
q16.2
q21
q22.1
q22.32
q23.3
q24.3
q25.3
q27
6,795 bp
60,656,000 bp
60,657,000 bp
60,658,000 bp
60,659,000 bp
60,660,000 bp
60,661,000 bp
Gene
hg38.subramanianT1.gtf
hg38.subramanianT2.gtf
transcripts.gtf
6p11.2
HML2_6q11.1

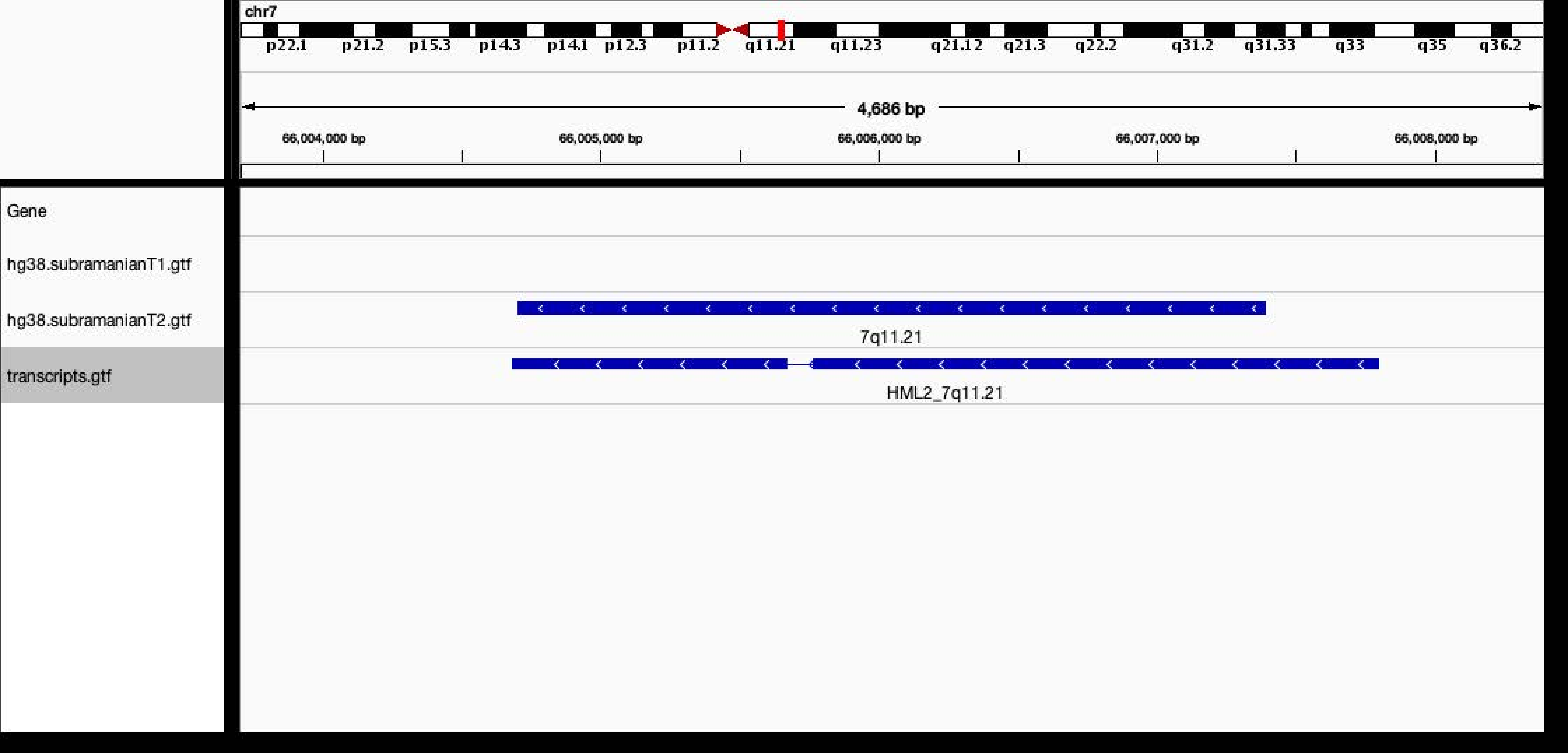

chr7
p22.1
p21.2
p15.3
p14.3
p14.1
p12.3
p11.2
q11.21
q11.23
q21.12
q21.3
q22.2
q31.2
q31.33
q33
q35
q36.2
4,686 bp
66,004,000 bp
66,005,000 bp
66,006,000 bp
66,007,000 bp
66,008,000 bp
Gene
hg38.subramanianT1.gtf
hg38.subramanianT2.gtf
transcripts.gtf
7q11.21
HML2_7q11.21

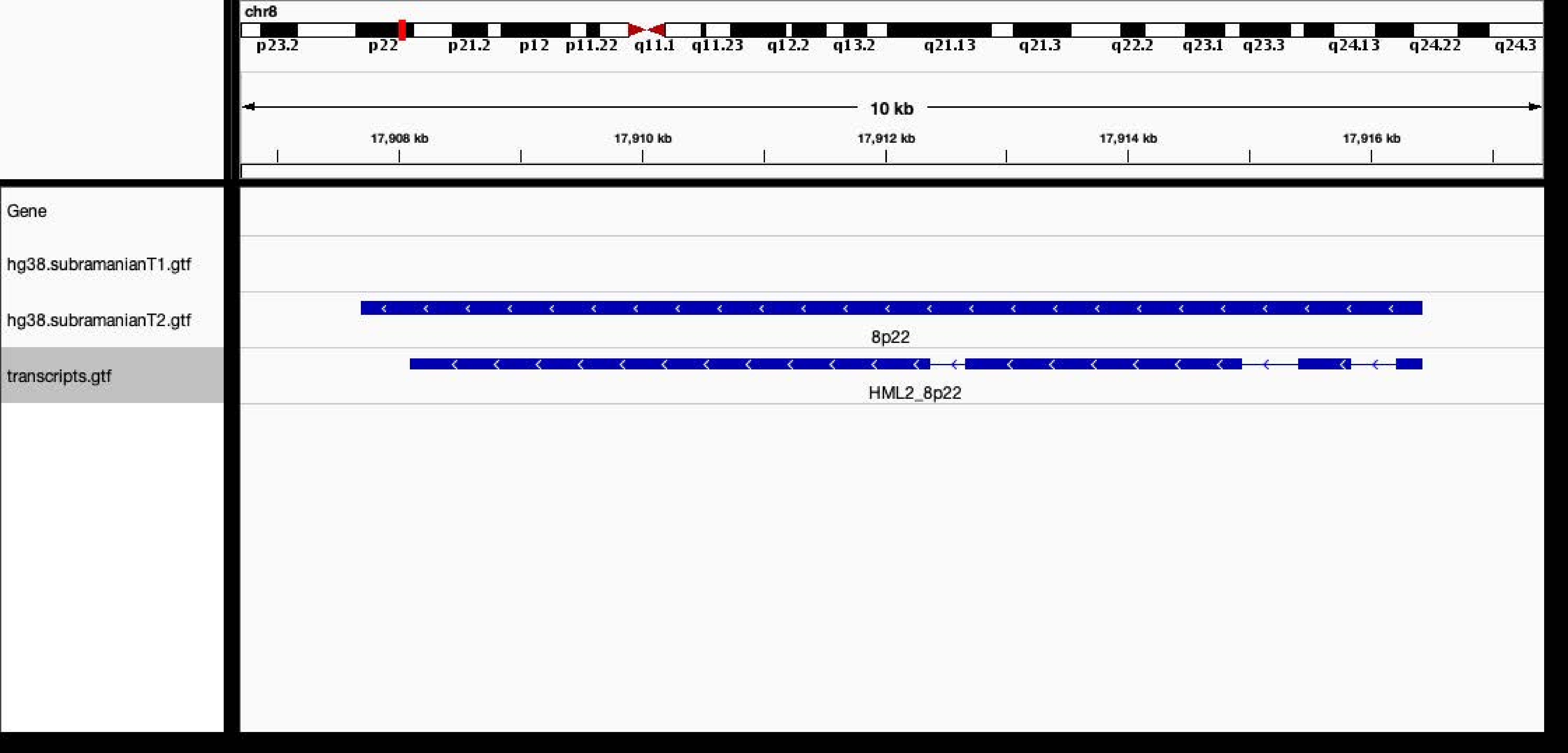

chr8
p23.2
p22
p21.2
p12
p11.22
q11.1
q11.23
q12.2
q13.2
q21.13
q21.3
q22.2
q23.1
q23.3
q24.13
q24.22
q24.3
10 kb
17,908 kb
17,910 kb
17,912 kb
17,914 kb
17,916 kb
Gene
hg38.subramanianT1.gtf
hg38.subramanianT2.gtf
transcripts.gtf
8p22
HML2_8p22

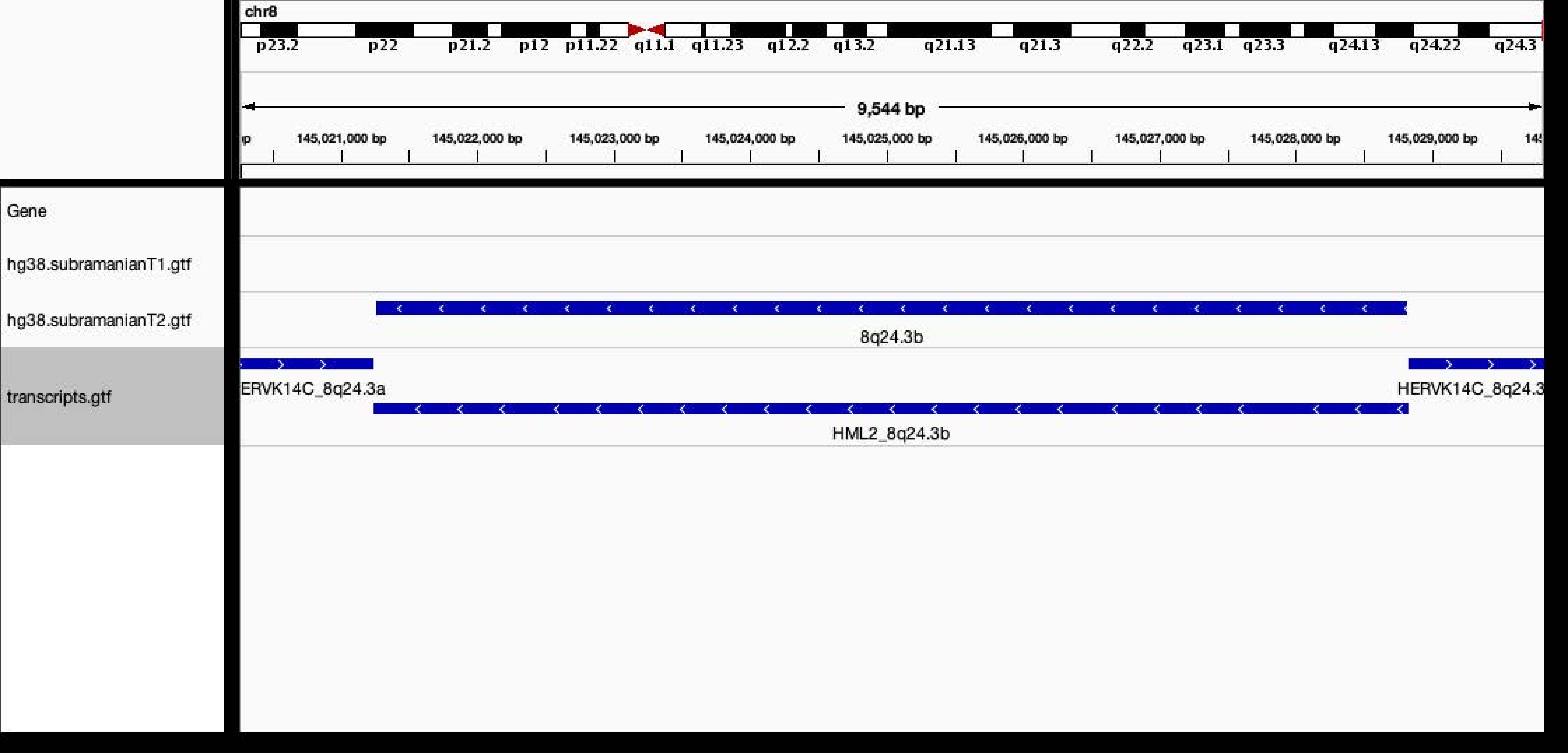

chr8
p23.2
p22
p21.2
p12
p11.22
q11.1
q11.23
q12.2
q13.2
q21.13
q21.3
q22.2
q23.1
q23.3
q24.13
q24.22
q24.3
9,544 bp
145,021,000 bp
145,022,000 bp
145,023,000 bp
145,024,000 bp
145,025,000 bp
145,026,000 bp
145,027,000 bp
145,028,000 bp
145,029,000 bp
Gene
hg38.subramanianT1.gtf
hg38.subramanianT2.gtf
8q24.3b
transcripts.gtf
ERVK14C_8q24.3a
HERVK14C_8q24.3
HML2_8q24.3b

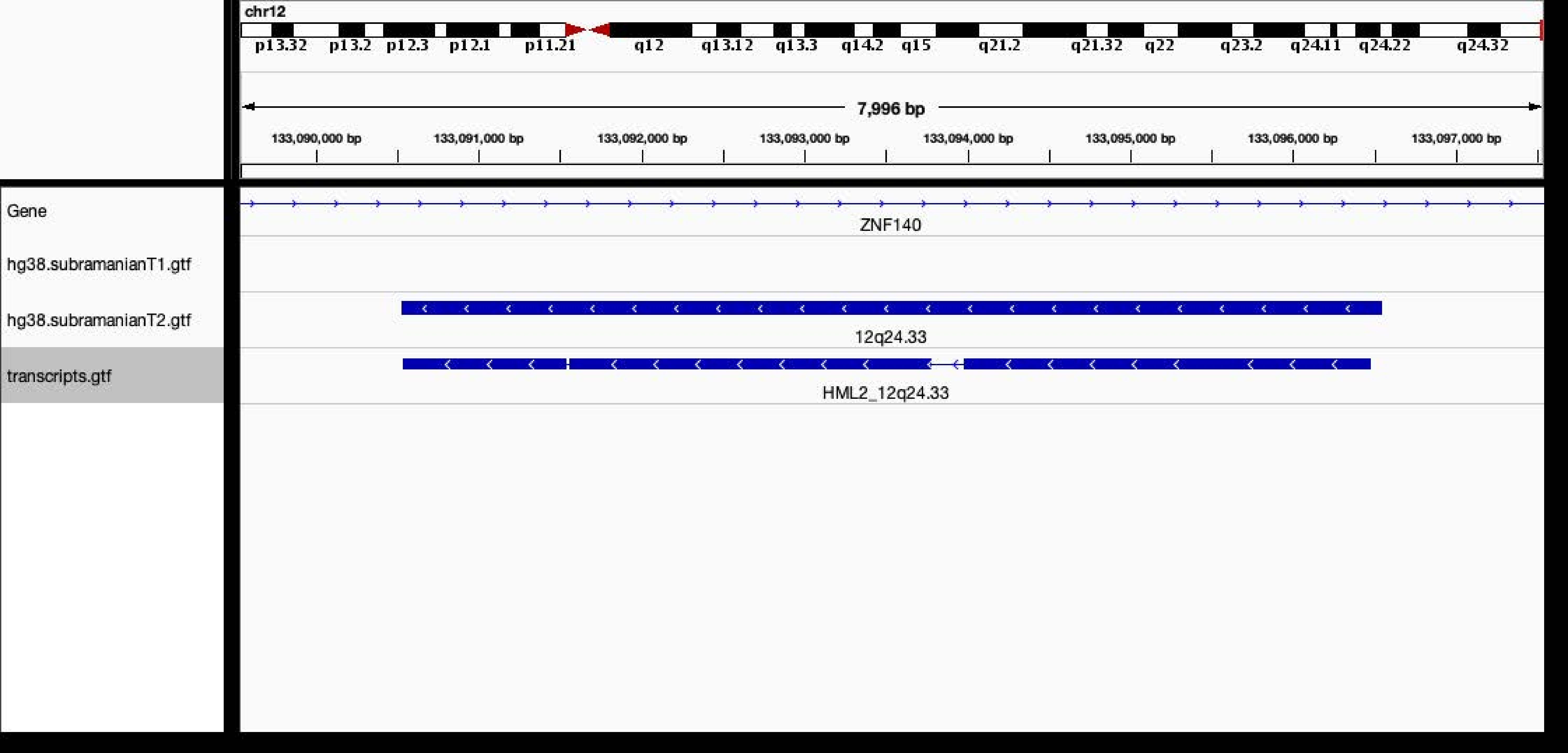
chr12
p13.32
p13.2
p12.3
p12.1
p11.21
q12
q13.12
q13.3
q14.2
q15
q21.2
q21.32
q22
q23.2
q24.11
q24.22
q24.32
7,996 bp
133,090,000 bp
133,091,000 bp
133,092,000 bp
133,093,000 bp
133,094,000 bp
133,095,000 bp
133,096,000 bp
133,097,000 bp
Gene
ZNF140
hg38.subramanianT1.gtf
hg38.subramanianT2.gtf
12q24.33
transcripts.gtf
HML2_12q24.33

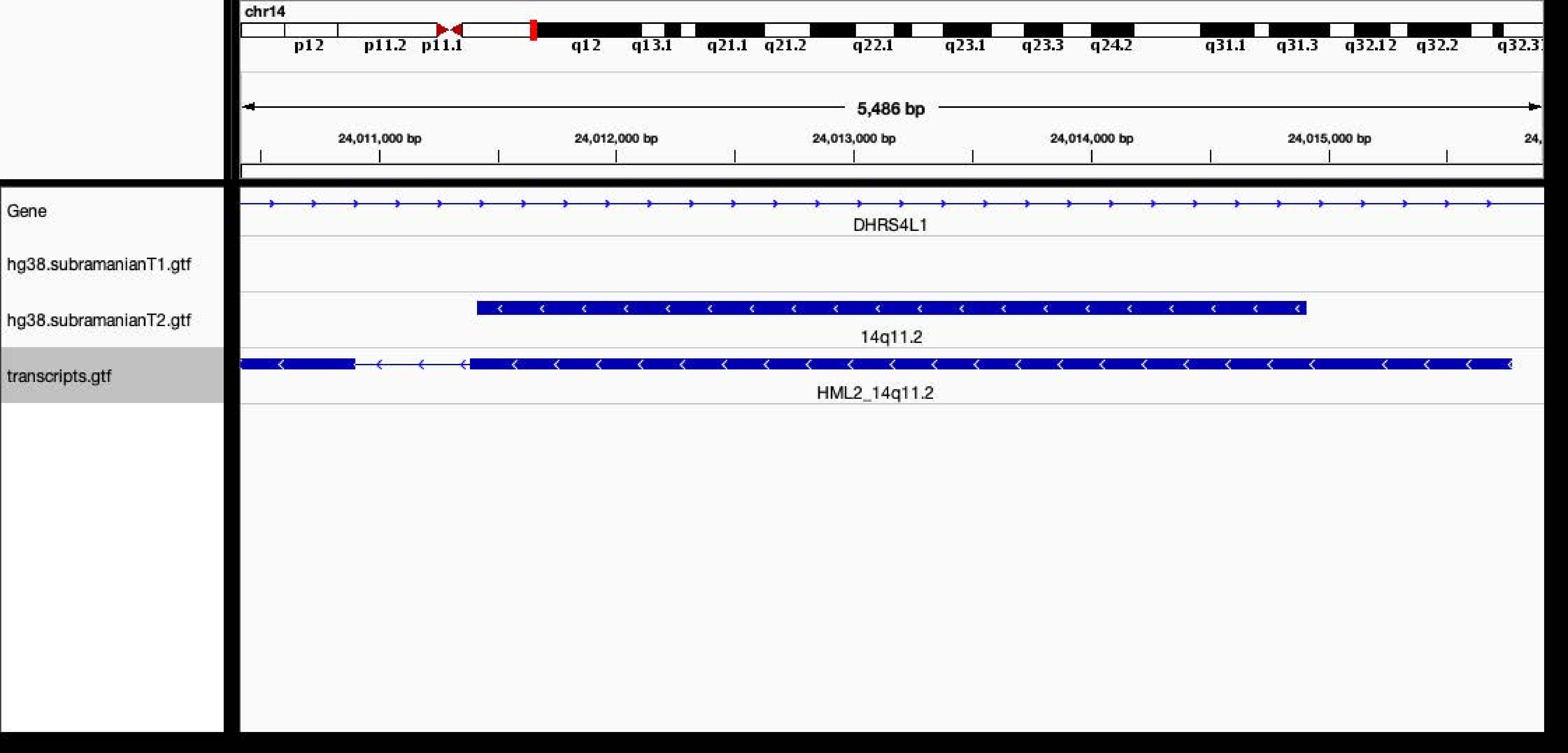
chr14
p12
p11.2
p11.1
q12
q13.1
q21.1
q21.2
q22.1
q23.1
q23.3
q24.2
q31.1
q31.3
q32.12
q32.2
5,486 bp
24,011,000 bp
24,012,000 bp
24,013,000 bp
24,014,000 bp
24,015,000 bp
Gene
hg38.subramanianT1.gtf
hg38.subramanianT2.gtf
transcripts.gtf
DHRS4L1
14q11.2
HML2_14q11.2

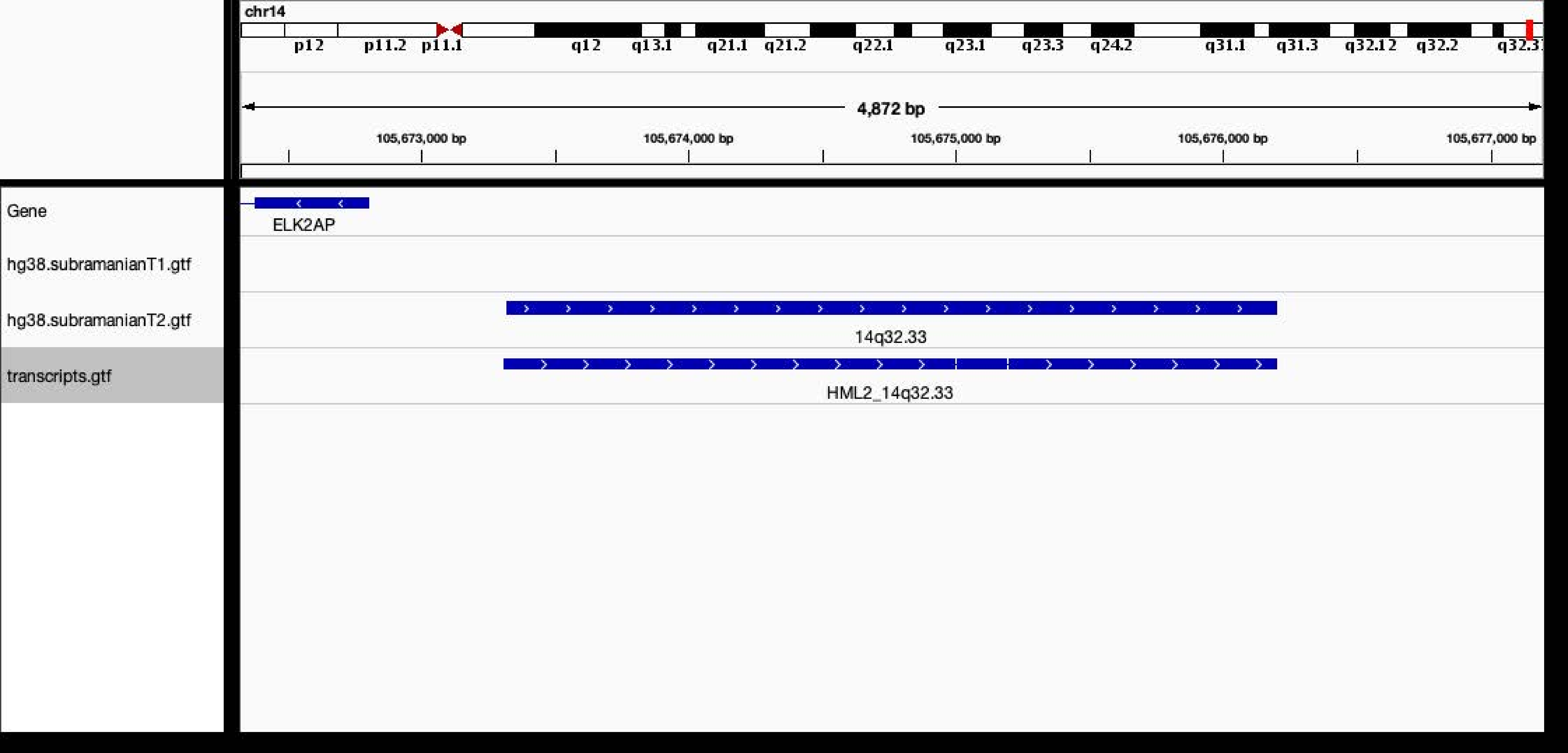

chr14
p12
p11.2
p11.1
q12
q13.1
q21.1
q21.2
q22.1
q23.1
q23.3
q24.2
q31.1
q31.3
q32.12
q32.2
4,872 bp
105,673,000 bp
105,674,000 bp
105,675,000 bp
105,676,000 bp
105,677,000 bp
Gene
ELK2AP
hg38.subramanianT1.gtf
hg38.subramanianT2.gtf
14q32.33
transcripts.gtf
HML2_14q32.33

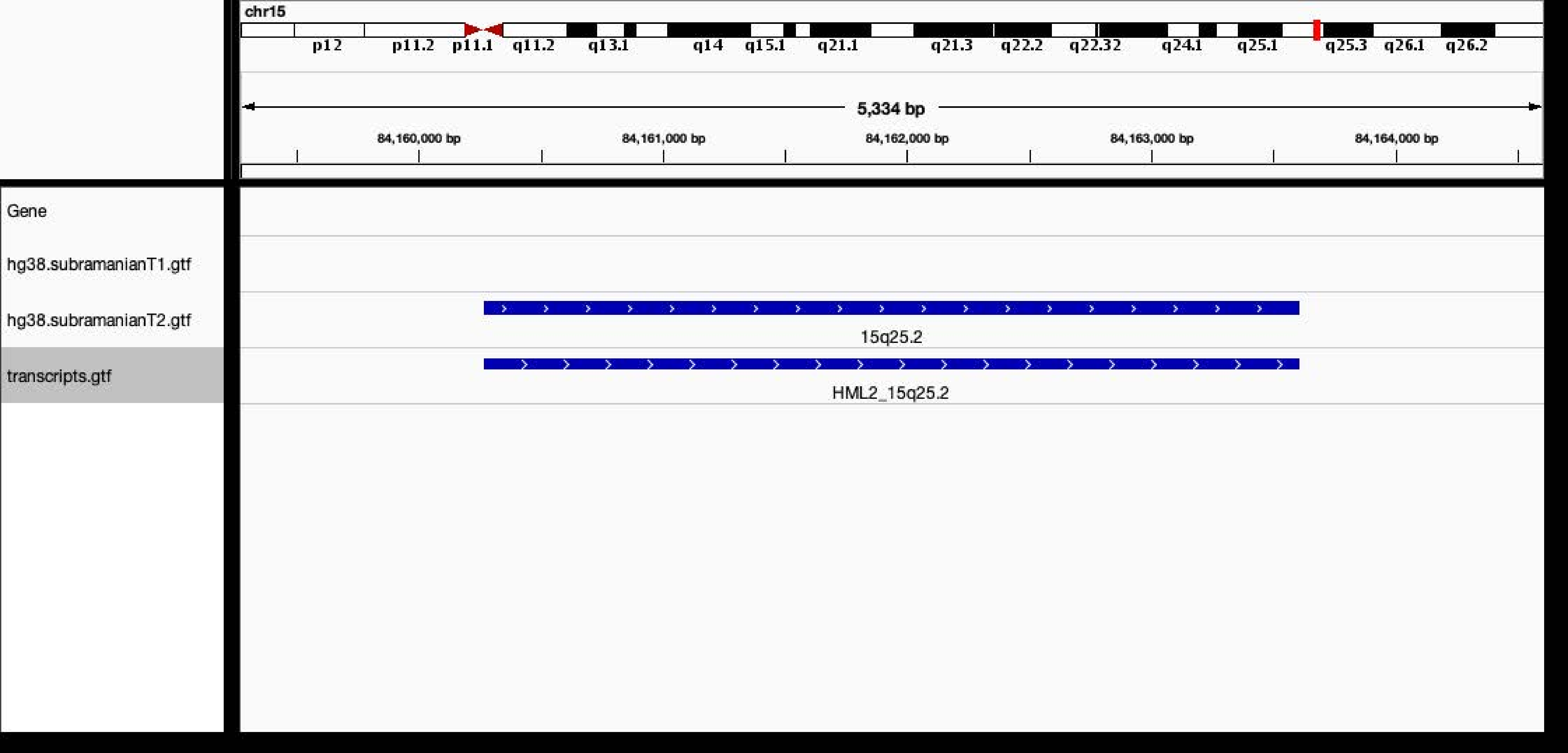
chr15
p12
p11.2
p11.1
q11.2
q13.1
q14
q15.1
q21.1
q21.3
q22.2
q22.32
q24.1
q25.1
q25.3
q26.1
q26.2
5,334 bp
84,160,000 bp
84,161,000 bp
84,162,000 bp
84,163,000 bp
84,164,000 bp
Gene
hg38.subramanianT1.gtf
hg38.subramanianT2.gtf
transcripts.gtf
15q25.2
HML2_15q25.2

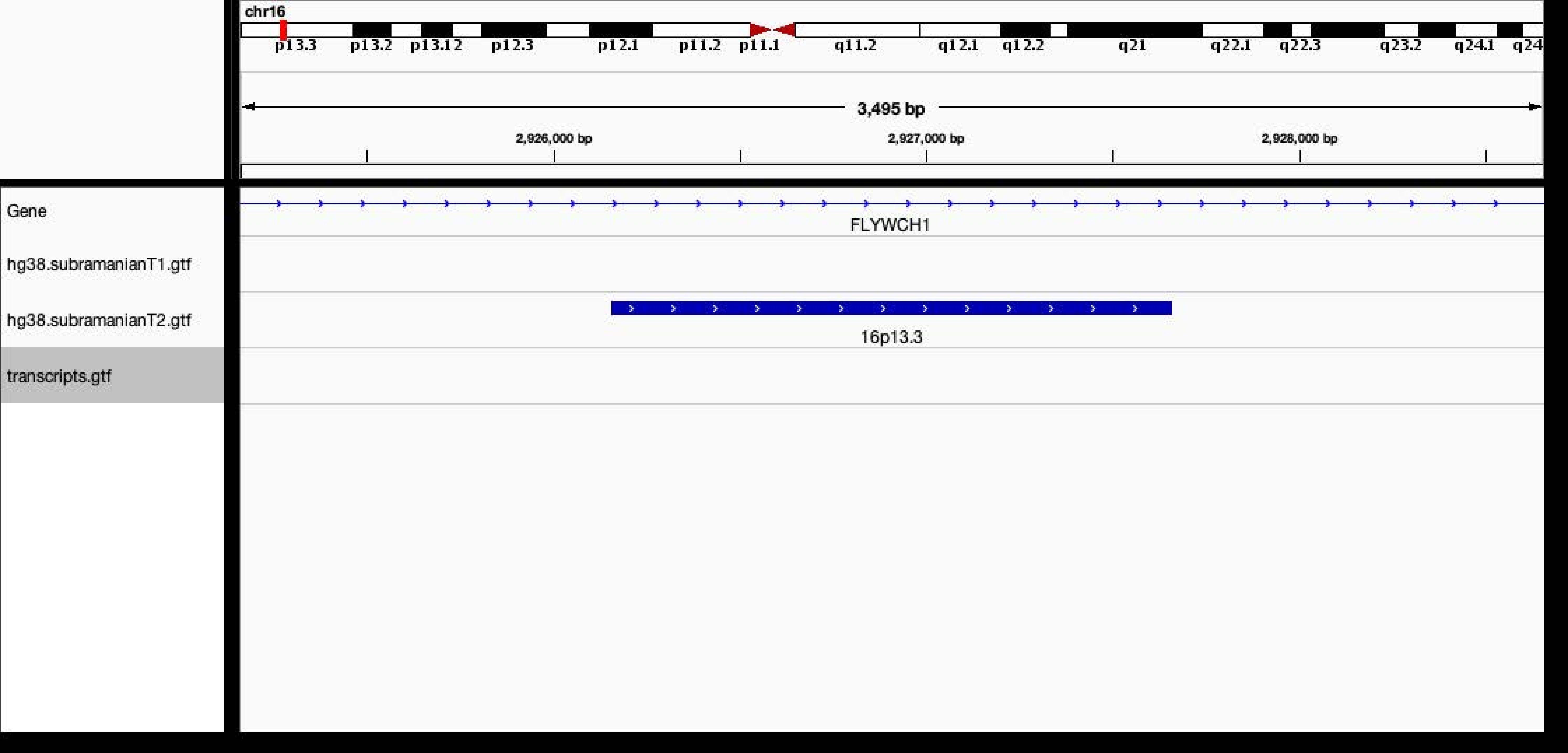
chr16
p13.3 p13.2 p13.12 p12.3 p12.1 p11.2 p11.1 q11.2 q12.1 q12.2 q21 q22.1 q22.3 q23.2 q24.1 q24
3,495 bp
2,926,000 bp
2,927,000 bp
2,928,000 bp
Gene
FLYWCH1
hg38.subramanianT1.gtf
hg38.subramanianT2.gtf
16p13.3
transcripts.gtf

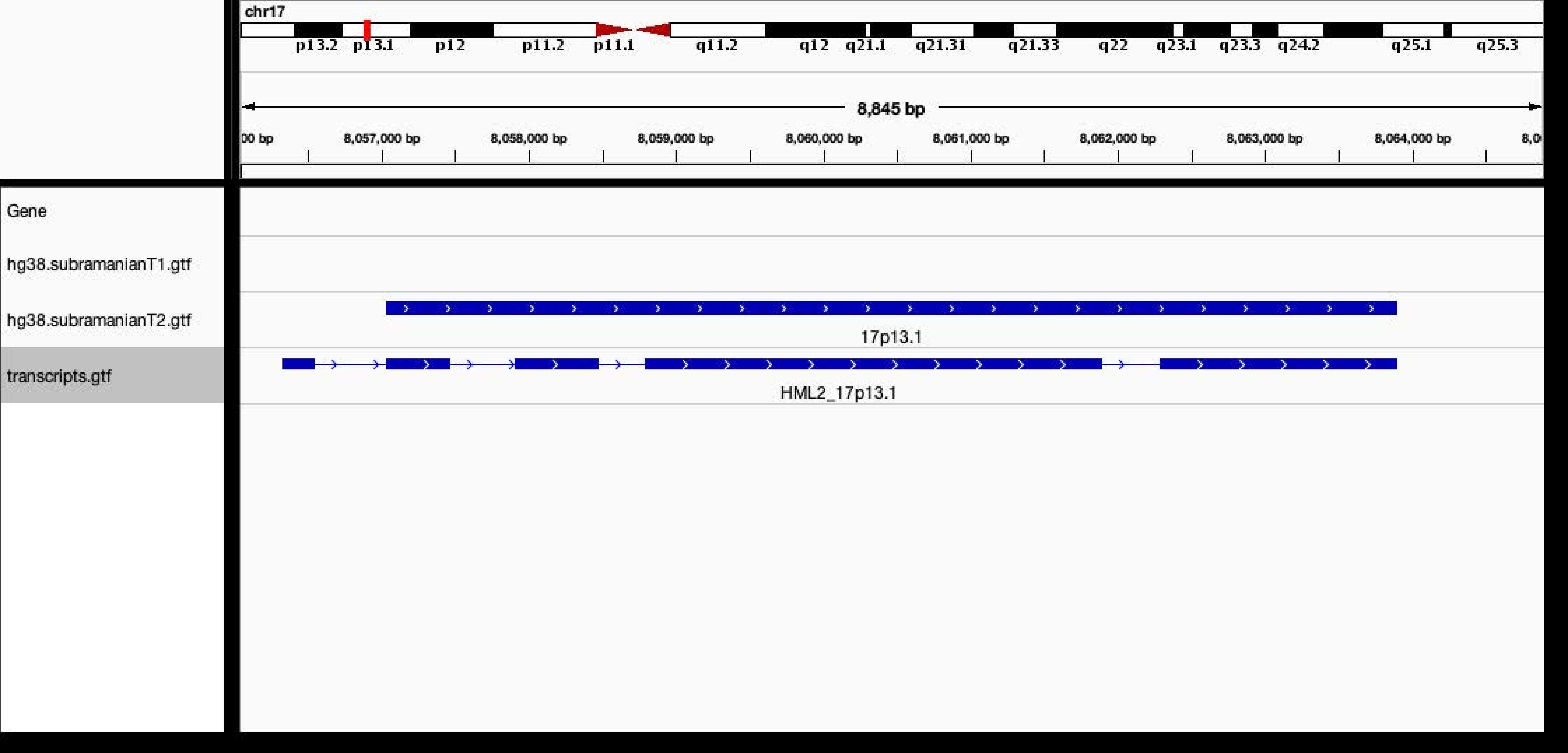
chr17
p13.2 p13.1 p12 p11.2 p11.1 q11.2 q12 q21.1 q21.31 q21.33 q22 q23.1 q23.3 q24.2 q25.1 q25.3
8,845 bp
8,057,000 bp
8,058,000 bp
8,059,000 bp
8,060,000 bp
8,061,000 bp
8,062,000 bp
8,063,000 bp
8,064,000 bp
Gene
hg38.subramanianT1.gtf
hg38.subramanianT2.gtf
17p13.1
transcripts.gtf
HML2_17p13.1

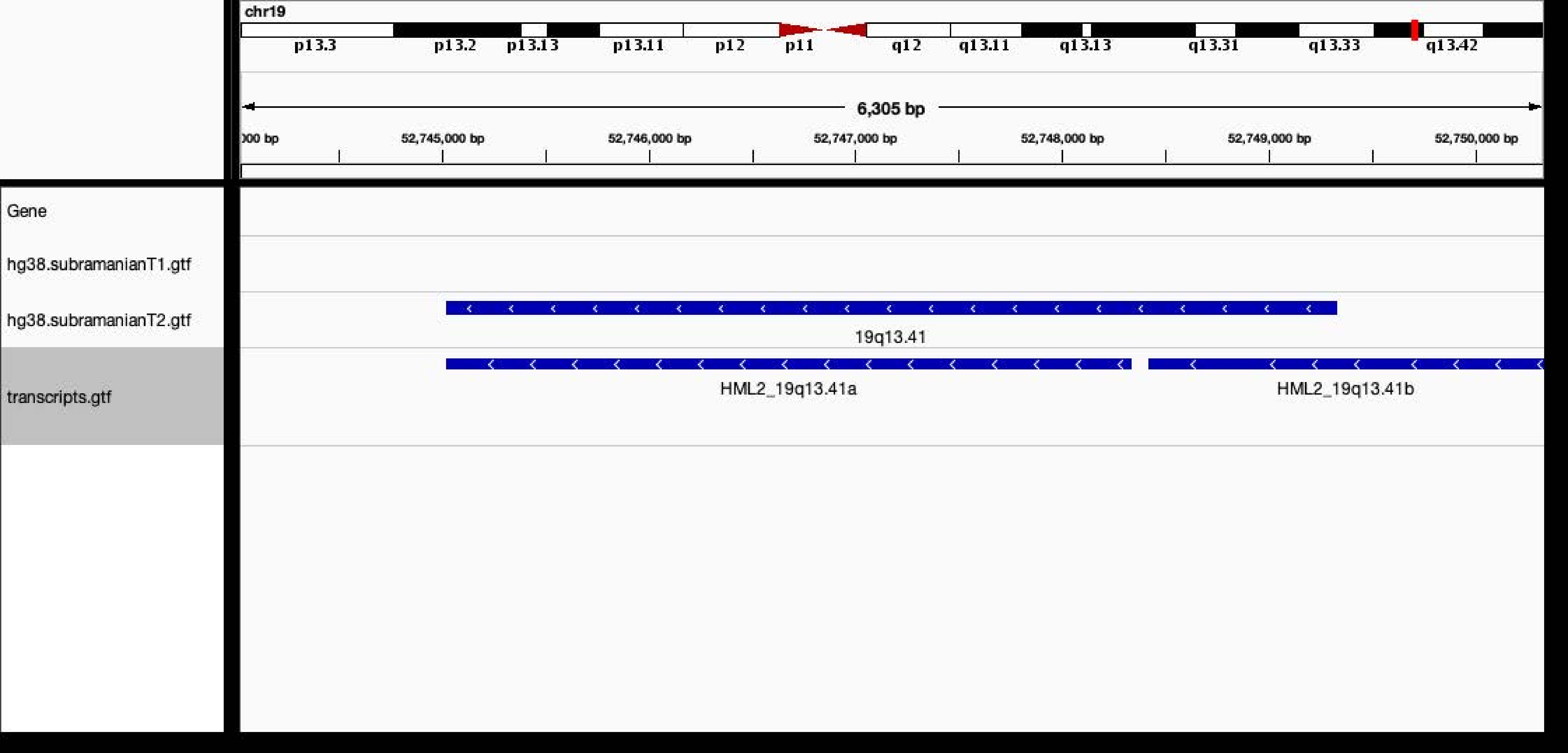
chr19
p13.3
p13.2
p13.13
p13.11
p12
p11
q12
q13.11
q13.13
q13.31
q13.33
q13.42
6,305 bp
52,745,000 bp
52,746,000 bp
52,747,000 bp
52,748,000 bp
52,749,000 bp
52,750,000 bp
Gene
hg38.subramanianT1.gtf
hg38.subramanianT2.gtf
transcripts.gtf
19q13.41
HML2_19q13.41a
HML2_19q13.41b

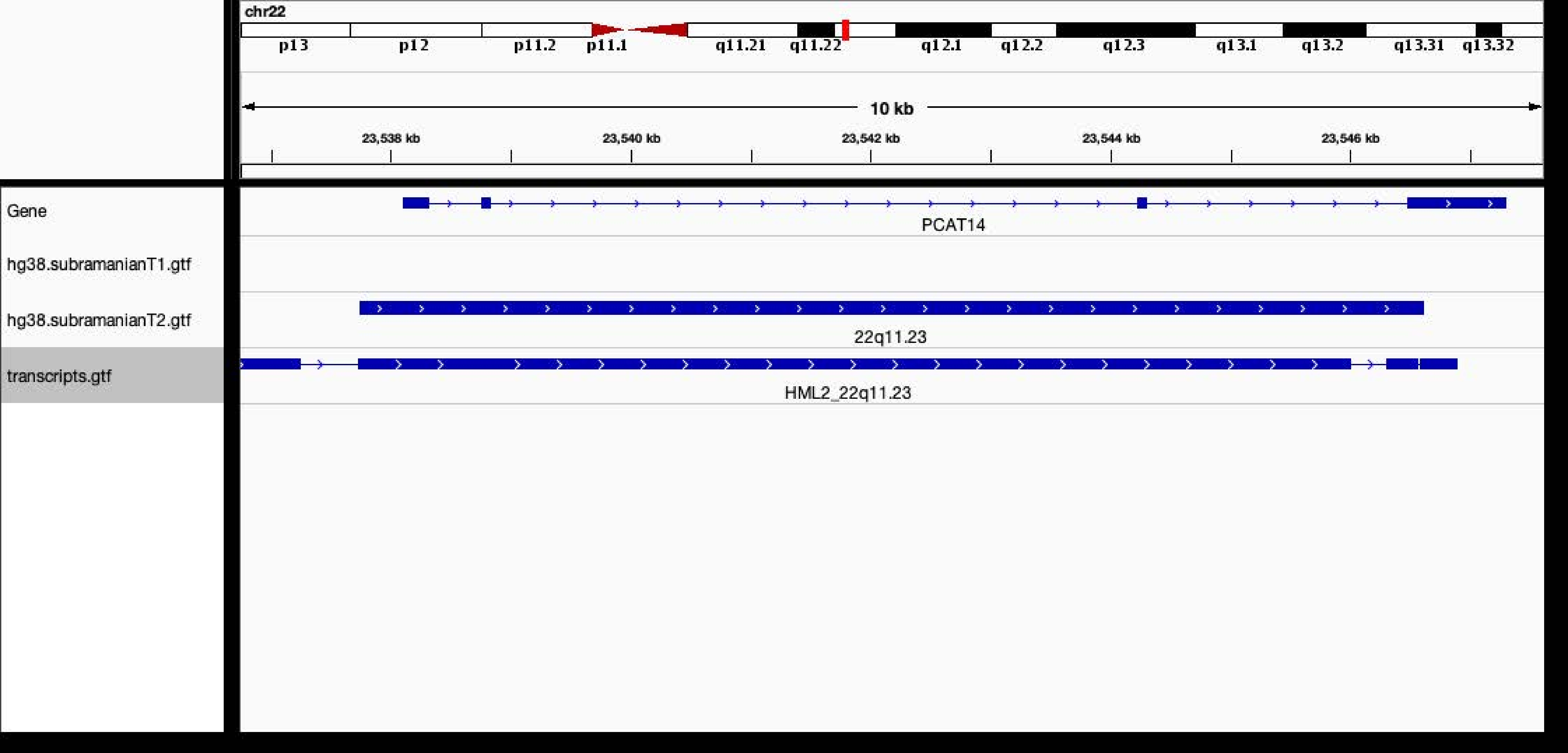
chr22
p13
p12
p11.2
p11.1
q11.21
q11.22
q12.1
q12.2
q12.3
q13.1
q13.2
q13.31
q13.32
10 kb
23,538 kb
23,540 kb
23,542 kb
23,544 kb
23,546 kb
Gene
PCAT14
hg38.subramanianT1.gtf
hg38.subramanianT2.gtf
22q11.23
transcripts.gtf
HML2_22q11.23

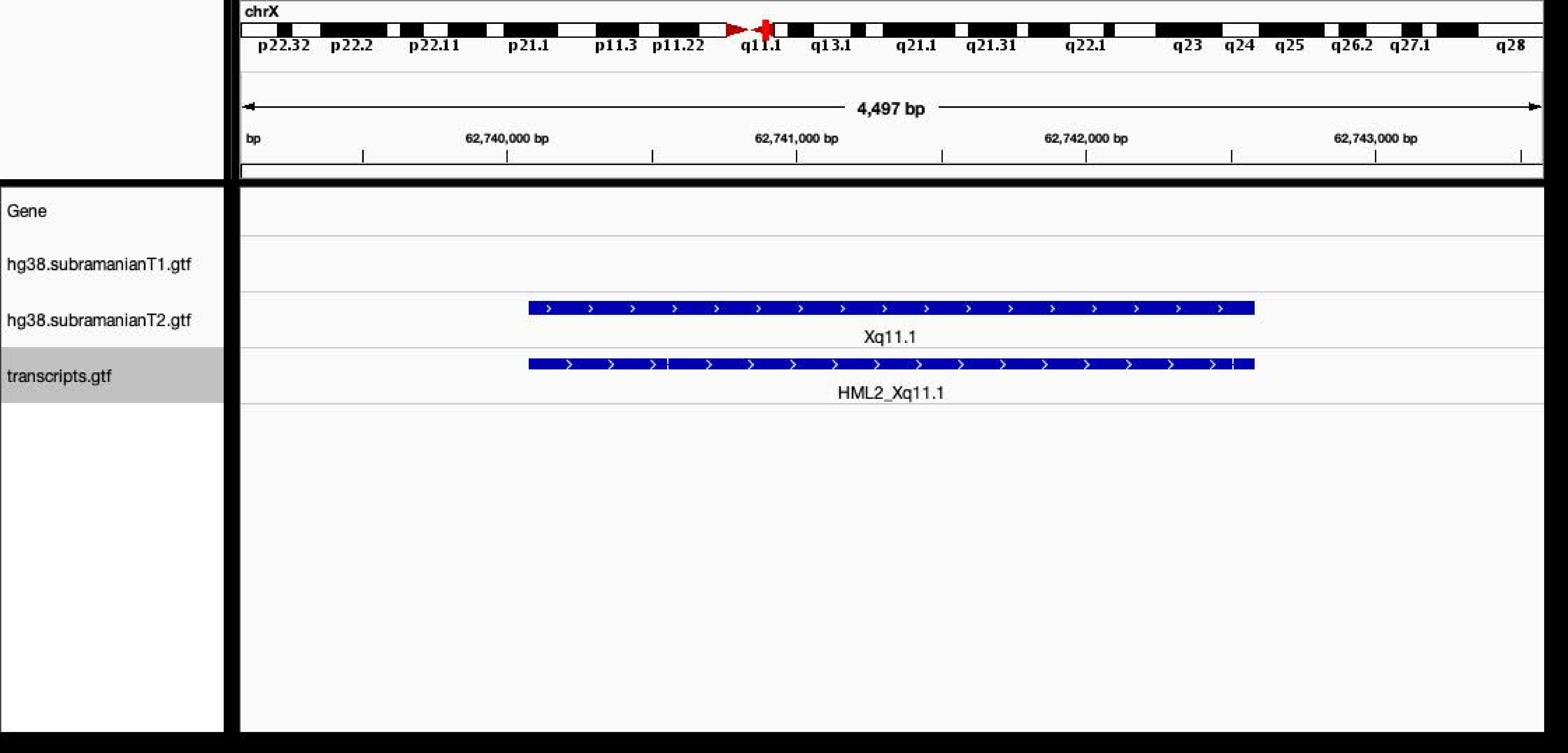
chrX
p22.32
p22.2
p22.11
p21.1
p11.3
p11.22
q11.1
q13.1
q21.1
q21.31
q22.1
q23
q24
q25
q26.2
q27.1
q28
4,497 bp
bp
62,740,000 bp
62,741,000 bp
62,742,000 bp
62,743,000 bp
Gene
hg38.subramanianT1.gtf
hg38.subramanianT2.gtf
Xq11.1
transcripts.gtf
HML2_Xq11.1

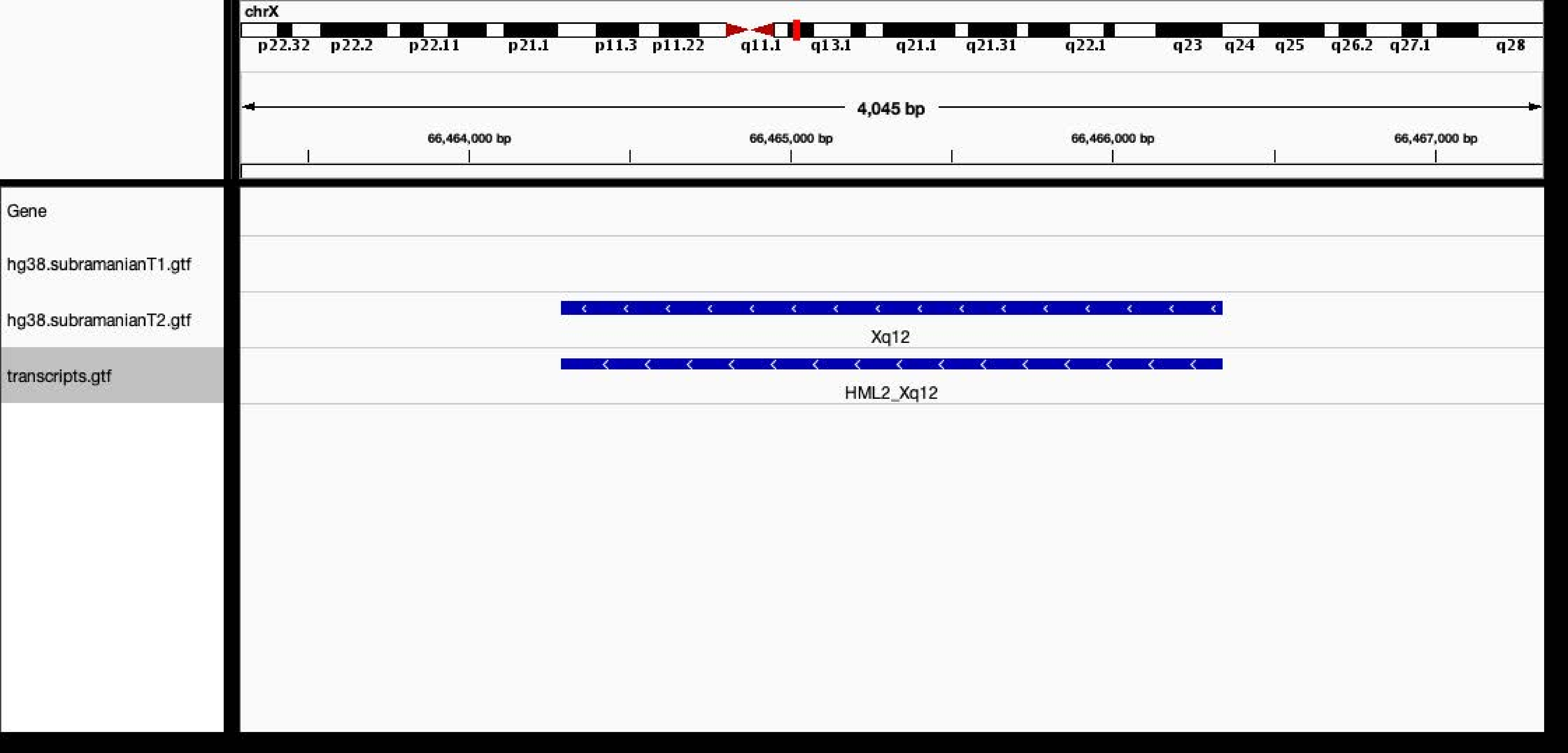
chrX
p22.32
p22.2
p22.11
p21.1
p11.3
p11.22
q11.1
q13.1
q21.1
q21.31
q22.1
q23
q24
q25
q26.2
q27.1
q28
4,045 bp
66,464,000 bp
66,465,000 bp
66,466,000 bp
66,467,000 bp
Gene
hg38.subramanianT1.gtf
hg38.subramanianT2.gtf
Xq12
transcripts.gtf
HML2_Xq12

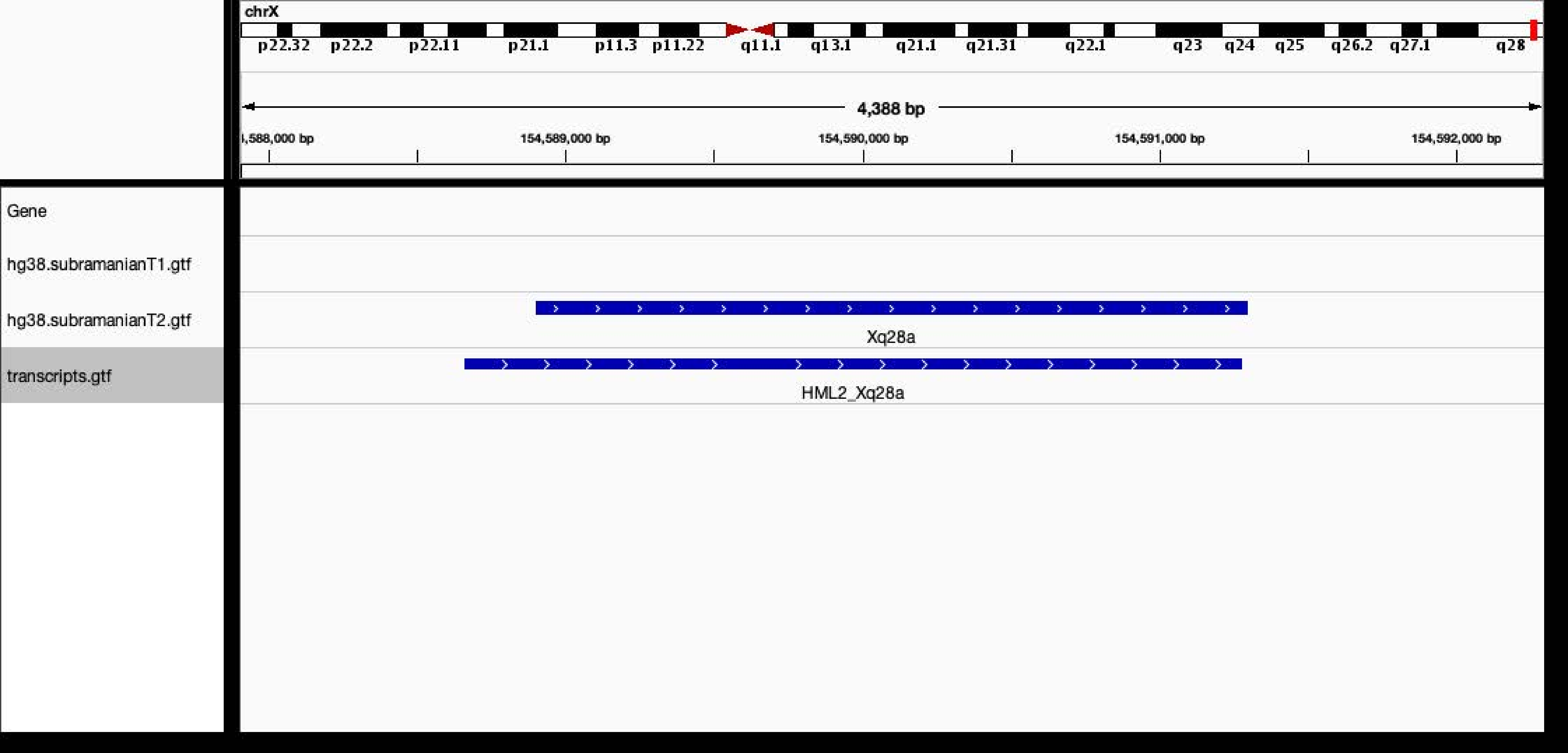
chrX
p22.32 p22.2 p22.11 p21.1 p11.3 p11.22 q11.1 q13.1 q21.1 q21.31 q22.1 q23 q24 q25 q26.2 q27.1 q28
4,388 bp
,588,000 bp
154,589,000 bp
154,590,000 bp
154,591,000 bp
154,592,000 bp
Gene
hg38.subramanianT1.gtf
hg38.subramanianT2.gtf
transcripts.gtf
Xq28a
HML2_Xq28a

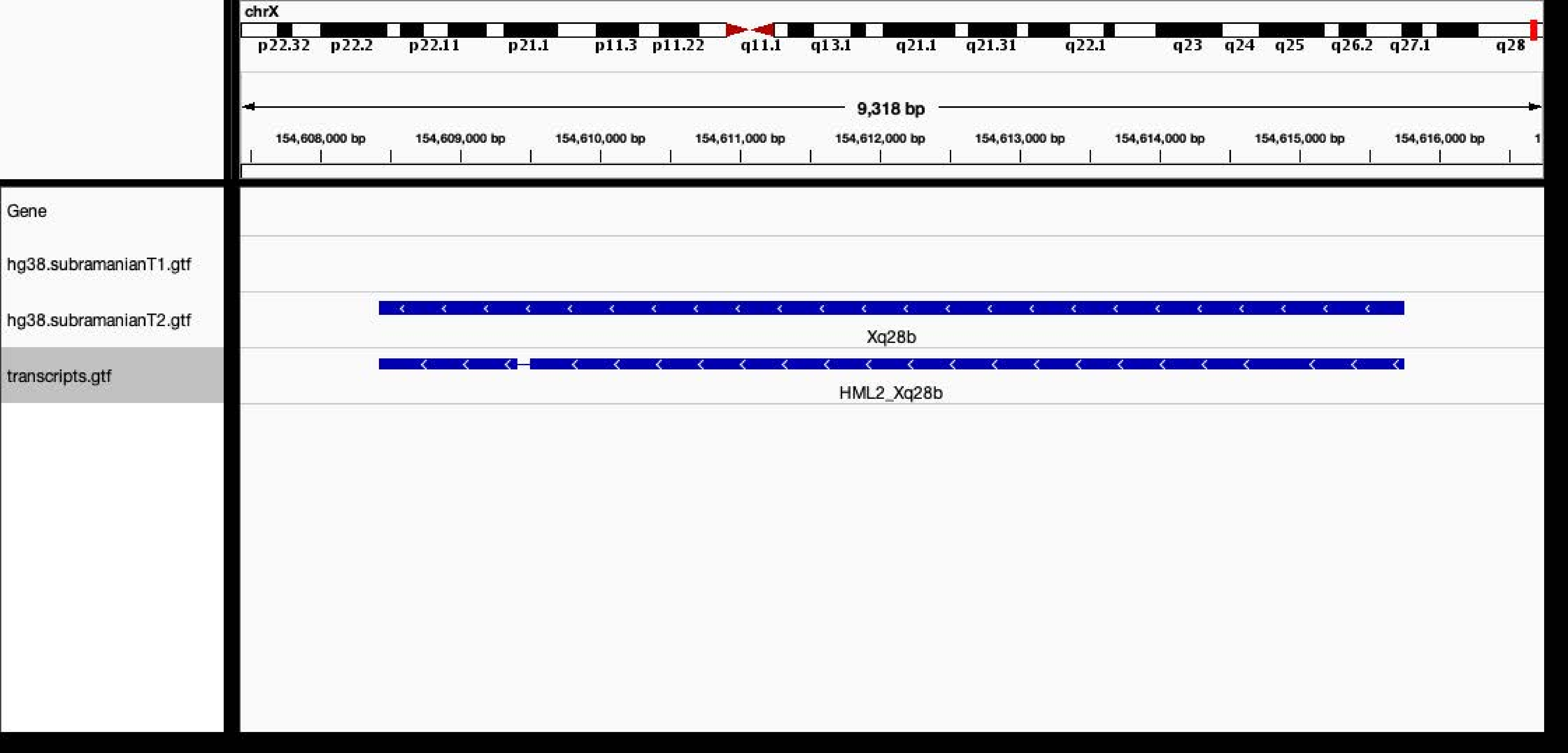

chrX
p22.32 p22.2 p22.11 p21.1 p11.3 p11.22 q11.1 q13.1 q21.1 q21.31 q22.1 q23 q24 q25 q26.2 q27.1 q28
9,318 bp
154,608,000 bp 154,609,000 bp 154,610,000 bp 154,611,000 bp 154,612,000 bp 154,613,000 bp 154,614,000 bp 154,615,000 bp 154,616,000 bp
Gene
hg38.subramanianT1.gtf
hg38.subramanianT2.gtf
transcripts.gtf
Xq28b
HML2_Xq28b

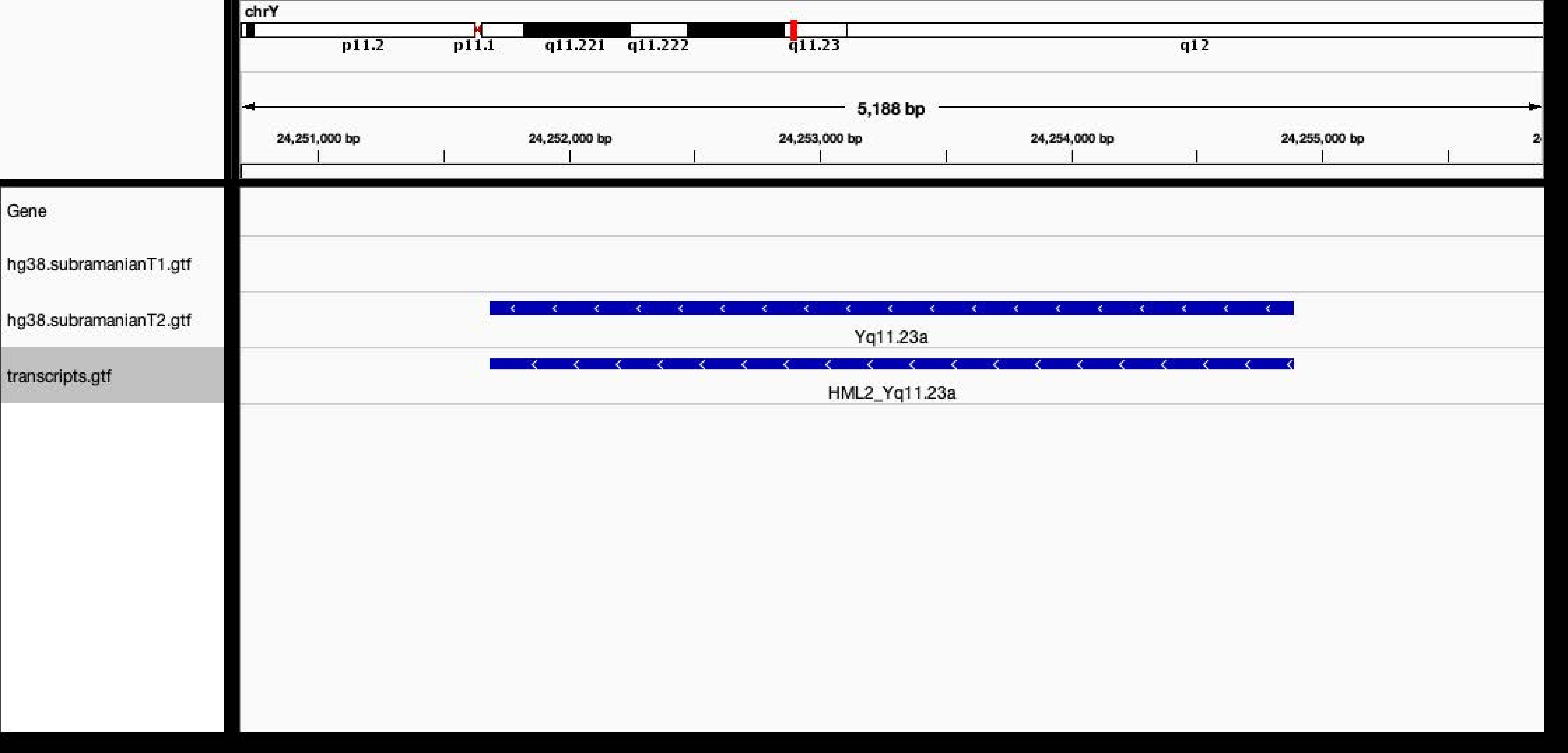
chrY
p11.2
p11.1
q11.221
q11.222
q11.23
q12
5,188 bp
24,251,000 bp
24,252,000 bp
24,253,000 bp
24,254,000 bp
24,255,000 bp
Gene
hg38.subramanianT1.gtf
hg38.subramanianT2.gtf
Yq11.23a
transcripts.gtf
HML2_Yq11.23a

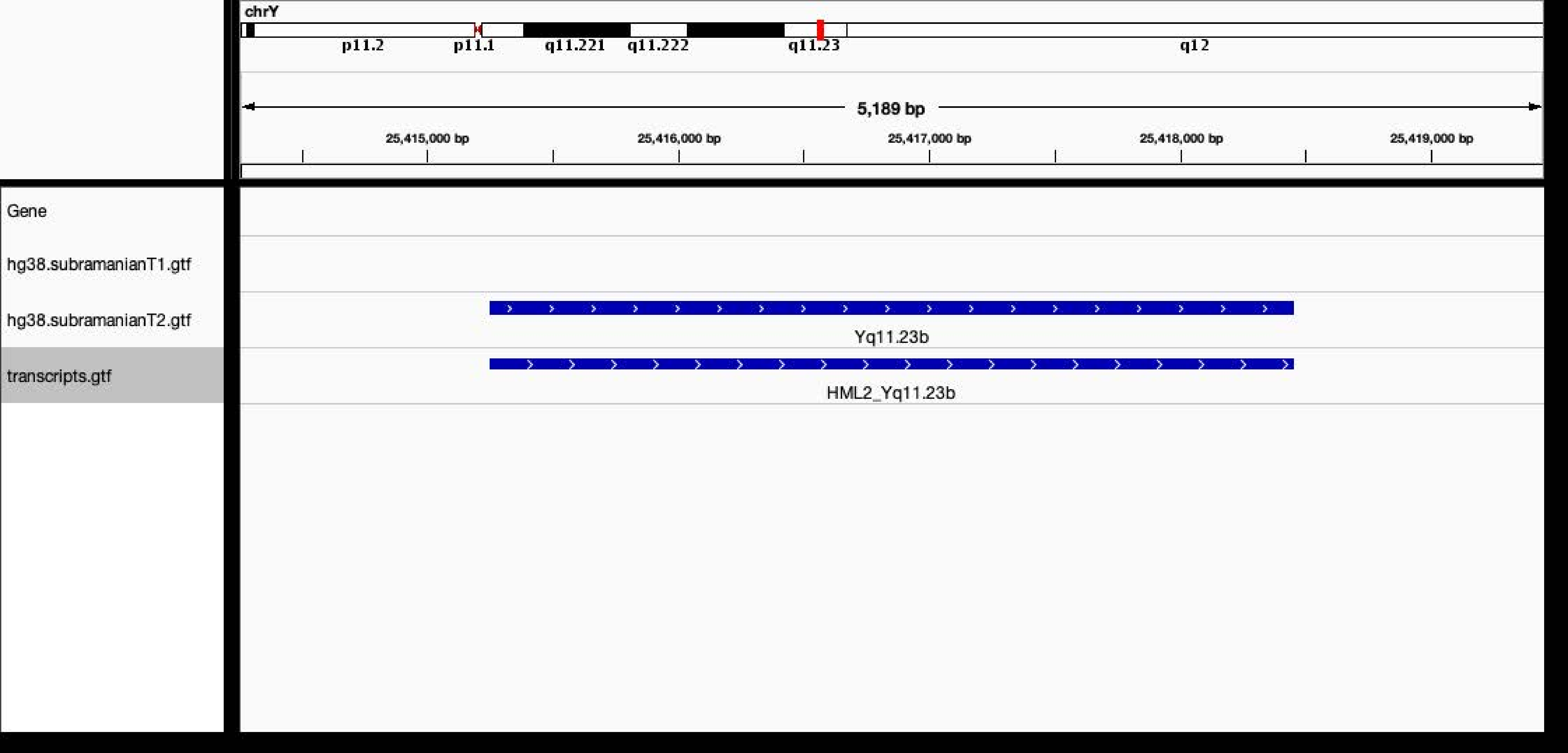

chrY
p11.2
p11.1
q11.221
q11.222
q11.23
q12
5,189 bp
25,415,000 bp
25,416,000 bp
25,417,000 bp
25,418,000 bp
25,419,000 bp
Gene
hg38.subramanianT1.gtf
hg38.subramanianT2.gtf
transcripts.gtf
Yq11.23b
HML2_Yq11.23b
